# Regulatory Mechanisms for Transcriptional Bursting Revealed by an Event-Based Model

**DOI:** 10.34133/research.0253

**Published:** 2023-10-24

**Authors:** Renjie Wu, Bangyan Zhou, Wei Wang, Feng Liu

**Affiliations:** ^1^ National Laboratory of Solid State Microstructures, Department of Physics, and Collaborative Innovation Center of Advanced Microstructures, Nanjing University, Nanjing 210093, P. R. China.; ^2^ Institute for Brain Sciences, Nanjing University, Nanjing 210093, P. R. China.

## Abstract

Gene transcription often occurs in discrete bursts, and it can be difficult to deduce the underlying regulatory mechanisms for transcriptional bursting with limited experimental data. Here, we categorize numerous states of single eukaryotic genes and identify 6 essential transcriptional events, each comprising a series of state transitions; transcriptional bursting is characterized as a sequence of 4 events, capable of being organized in various configurations, in addition to the beginning and ending events. By associating transcriptional kinetics with mean durations and recurrence probabilities of the events, we unravel how transcriptional bursting is modulated by various regulators including transcription factors. Through analytical derivation and numerical simulation, this study reveals key state transitions contributing to transcriptional sensitivity and specificity, typical characteristics of burst profiles, global constraints on intrinsic transcriptional noise, major regulatory modes in individual genes and across the genome, and requirements for fast gene induction upon stimulation. It is illustrated how biochemical reactions on different time scales are modulated to separately shape the durations and ordering of the events. Our results suggest that transcriptional patterns are essentially controlled by a shared set of transcriptional events occurring under specific promoter architectures and regulatory modes, the number of which is actually limited.

## Introduction

Gene transcription is a complicated dynamic process integrating regulatory signals with genetic information. mRNAs can be produced at constant rates or in short intervals followed by long periods of inactivity, termed transcriptional bursting [1–3]. Although burst traces are qualitatively similar across organisms, the duration of the burst cycle varies remarkably, ranging from minutes to hours [4]. Does this great variability reflect diverse molecular mechanisms or stem from a shared set of highly adjustable molecular events? Similarly, molecular processes involved in transcription typically last from milliseconds to hours [3–5]. How can they be affected by regulators to underlie transcriptional bursting? How can bursting kinetics be modulated across such a wide range of time scales? These topics are fundamental to comprehending the regulation of gene transcription.

Live-cell fluorescence measurements have yielded large amounts of single-cell data [3,6,7]. Durations of transcriptionally active and inactive states and burst size were extracted to characterize transcriptional bursting and expression noise. To theoretically interpret them, phenomenological models have been built upon independent effective processes [8–14], enabling the association between regulatory signals and experimental observables. By incorporating more transcriptional details such as multistep initiation and multistep degradation of mRNA, newly developed models gain more insights into regulatory mechanisms [15–22]. To unravel gene regulation and promoter configurations through the lens of input–output relationships, however, precise knowledge of biochemical reaction mechanisms is essential; yet, such knowledge is often unavailable and must be inferred on the basis of assumptions. This could result in discrepancy between model predictions and experimental data, ignorance of global constraint on transcriptional noise, and inaccurate mapping between rate-limiting steps and effective processes under limiting conditions when deducing the underlying regulatory mechanism [23,24]. A different modeling framework is required.

On the basis of a set of common biochemical reactions essential to mRNA synthesis, a generic model has been developed, which is compatible with those simplified models and fills in their gaps [25–27]. Nevertheless, there always exist additional gene-specific reactions; some common reactions actually comprise multiple steps, and the transition process often occurs through distinct paths, leaving a memory in the transcriptional path [9,28]. While these details could be incorporated to construct more intricate models, we may develop a simpler and more computationally efficient method using queuing theory, which is widely leveraged to understand the behavior of systems with queues. Indeed, queuing models [28–30] were proposed to analyze empirical data on mRNA copy numbers and waiting-time distributions of biochemical reactions while neglecting the underlying molecular processes. To elucidate the molecular mechanism of gene regulation, we may focus on essential transcriptional events, with each comprising multiple molecular processes, and simplify the transcription into a sequence of events.

Gene transcription is modulated by various regulatory factors, such as transcription factors (TFs) that bind DNA to activate transcription and modifiers and remodelers that induce changes to histone markers and nucleosome arrays to overcome structural barriers [31]. They may exert a wide influence on transcription by altering the duration and directionality of molecular processes [32–34]. To unravel the roles for regulators in gene expression, it is essential to differentiate their impacts on rate-limiting steps and probability flows of event transitions in transcription cycle.

Here, we first propose a network of first-order Markovian biochemical reactions. Because transcriptional bursting is composed of inactive and active phases involving 3 major stages, i.e., chromatin opening for promoter access, assembly of the preinitiation complex (PIC), and mRNA production, all gene states can be divided into 5 sets to separate the stages, with functional state transitions classified into 9 categories. A series of state transitions is combined into an event, and a 6-event model is developed for transcriptional bursting. This model is generic and requires no free parameters. Transcriptional commonality is reflected in the sequential order of the 4 events, while gene specificity is embodied in the duration distributions and ordering of the events. The kinetic features of transcription, such as the mean mRNA number, burst size, burst frequency, and duration of the (in)active phase, are interconnected and functions of 6 model parameters depending on the regulator concentration (i.e., 4 average event durations and 2 repetition probabilities).

On the basis of this event model, we clarify typical features of and intrinsic constraints on transcriptional bursting, illuminate how the regulatory mechanism can be inferred by fitting experimental data at the single-gene and genome levels, and explore the transient transcriptional dynamics upon stimulation. Both numerical and analytical results are presented to provide an integrated view of how reactions on different time scales are modulated to underpin specific burst profiles. The pivotal regulatory modes and their functional implications are elaborated in detail.

## Results

### Establishment of the event model

Although gene transcription exhibits heterogeneity, its common features allow for the development of a generic model for transcriptional dynamics. To reveal the role for regulatory factors in transcriptional modulation, we need establish a model based on transcriptional events, which is both accurate and simple enough for theoretical analysis.

**Fig. 1.**
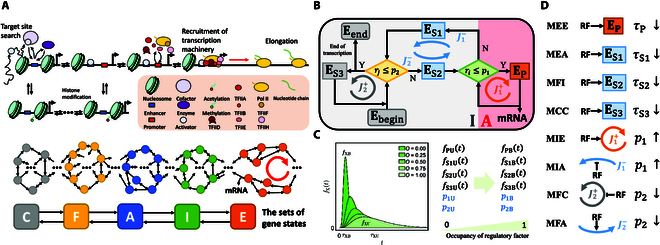
Model of the transcriptional cycle and transcriptional regulation. (A) Schematic of biochemical reactions underlying the gene states (top) and state transitions (middle; large circles denote gene states, while small circles denote omitted states). According to whether the core promoter is open or activated, all states are divided into 5 subsets (*C*, *F*, *A*, *I*, and *E*; bottom). (B) Flow-process diagram of the event-based model simplified from (A). There are 6 non-Markovian events (rectangles) and 2 checkpoints (diamonds). “Y” and “N” separately represent whether the criterion is satisfied or not; *r_i_* and *r_j_* are random numbers from the uniform distribution on the unit interval. Transcription begins with *E*_begin_, goes through *E*_S2_, *E*_P_, *E*_S1_, and *E*_S3_, and ends with *E*_end_. The ordering of the events is determined by *p*_1_ and *p*_2_, which are decided by the event flows (*J*_1_^+^, *J*_1_^−^, *J*_2_^+^, and *J*_2_^−^). mRNAs are synthesized in the active phase (*A*, red area) comprising a series of *E*_P_, while no mRNA is produced in the inactive phase (*I*, gray area) consisting of sequences of *E*_S1_, *E*_S2_, and *E*_S3_. (C) Gene transcription is simulated based on the distribution functions *f*_X_ (*X* = P, S1, S2, S3), *p*_1_ and *p*_2_, which depend on the regulator occupancy rate (*O*). “B” denotes regulators bound and “U” regulator unbound. The left panel schematically shows *f*_X_ for different *O* values. When *O* rises from 0 to 1, (*f*_XU_, *p*_1U_, *p*_2U_) turns into (*f*_XB_, *p*_1B_, *p*_2B_). (D) Eight basic regulatory modes in which regulator binding can promote transcription by decreasing the mean event duration *τ*_X_ and *p*_2_ or by increasing *p*_1_. Only 1 of the 6 bursting parameters is adjustable in each mode. The first “M” in the leftmost column stands for modulation, while “RF” stands for regulatory factor. The black arrows signify that RF binding accelerates the events or increases the event flows (*J*_1_^+^ or *J*_2_^−^), whereas the black lines with a flat head signify that RF binding decreases the event flows (*J*_1_^−^ or *J*_2_^+^).

We first proposed a network model of inducible eukaryotic transcription (Fig. 1A). The transcription begins with activators binding to the promoter/enhancer [35], which promotes the recruitment of cofactors, chromatin remodeling [34,36] and histone modifications [31,37]. General TFs (GTFs; including TFIIA, TFIIB, TFIID, TFIIE, TFIIF, and TFIIH) and RNA polymerase II (Pol II) are then recruited to the core promoter, forming the PIC. The enhancer-bound activator and the PIC are connected by the mediator complex [38,39] via enhancer-promoter proximity. The PIC turns into the open complex after the DNA template strand is positioned into the active center cleft of Pol II. With the preparations completed, Pol II gets away into elongation to synthesize nucleotide chains [40], while the remaining scaffold complex (composed of activators, TFIIA, TFIID, TFIIE, TFIIH, and mediator) on the promoter facilitates transcriptional reinitiation. During early transcript synthesis, the early elongation complex (EEC) retains a measurable tendency to undergo backtracking until Pol II pauses about 30-base-pair downstream of the transcription start site (i.e., promoter-proximal pausing) [41] and then is released for productive elongation, restricting the frequency of transcriptional initiation [42].

Each node in the network represents a gene state, and the transition between adjacent states is a Markov process (Fig. 1A). The same reactions may occur at diverse sites around the promoter, and some reactions are not exclusive with each other, leading to various transition paths between 2 states. All states are divided into 5 sets (Table 1), separately responsible for maintaining chromatin inaccessibility (*C*), chromatin remodeling and histone modification when the core promoter is a nucleosome-free region (*F*), PIC assembly (*A*), initiation of mRNA synthesis by Pol II and promoter escape (*I*), and promoter-proximal elongation and pausing of Pol II (*E*). When a Pol II enters productive elongation (*P*), the gene state returns to set *I*, and another Pol II is recruited for transcription reinitiation. Each state can transition to a state in the same or adjacent set.

**Table 1.** Gene state sets.

State set	Description of state sets
C	States in which the access of GTFs to the core promoter is inhibited by occluded canonical nucleosomes or other macromolecules.
F	States in which the core promoter is a nucleosome free region and ready for GTF recruitment.
A	States in which the core promoter is occupied by GTFs but the PIC has not yet formed completely.
I	States in which the scaffold complex is at the promoter without the EEC formed.
E	States in which Pol II undergoes promoter-proximal elongation until it enters productive elongation.

This network model is too complicated to be analyzed easily. To simplify it, we exploited queueing theory [28,43] to turn the network model into an event-based model. First, 9 types of functional transitions are specified: *C_F_*, *F_C_*, *F_A_*, *A_F_*, *A_I_*, *I_A_*, *I_E_*, *E_I_*, and *E_I_^P^*, each comprising a series of reactions with the same function (Table 2). *X_X′_* (*X*, *X′* ∈ *C*, *F*, *A*, *I*, or *E*) represents the process where the initial state stems from set *X* and all state transitions occur in *X* until the last state belongs to *X′*. *E_I_^P^* involves Pol II released into productive elongation, such that a nascent full-length transcript can be produced, whereas *E_I_* does not. The time scales of (*I_E_*, *E_I_*, *E_I_^P^*), (*F_A_*, *A_I_*, *I_A_*, *A_F_*), and (*C_F_*, *F_C_*) are in the order of seconds to minutes, minutes, and minutes to hours, respectively.

**Table 2.** Reactions in functional transitions.

Transition	Reactions during state transitions	
*C_F_*	Reactions that make the core promoter nucleosome-free to promote the recruitment of GTFs, e.g., removal of nucleosomes at the promoter, altering the accessibility of DNA on the surface of nucleosomes by remodelers, replacement with certain histone variants, or destabilization of internucleosome contacts by acetylation of lysine residues. Reactions that change the microenvironment when the promoter is occluded by canonical nucleosomes or other macromolecules different from GTFs, e.g., formation or disruption of topologically associating domain boundaries and changes in distribution modes and interactions of chromatin marks [36,75].	
*F_C_*	Reactions that change the microenvironment when the promoter is a nuclosome-free region [75].	The core promoter is wrapped around histone octamers to form canonical nucleosomes or occupied by other macromolecules [36].
*F_A_*		Binding of GTFs, especially TFIID [75–77].
*A_F_*	Reactions that change the microenvironment when the promoter is occupied by GTFs; enhancer-promoter proximity (hubs or looping) [75].	Dissociation of GTFs bound to the core promoter [75–77].
*A_I_*		Formation of the PIC scaffold [78–80].
*I_A_*	Reactions that change the microenvironment when the PIC scaffold is formed without the EEC on the DNA template; enhancer-promoter proximity [75,81].	Dissociation of the PIC scaffold components [78–80].
*I_E_*		Stabilizing the initially transcribing complex to form the EEC via promoter escape, e.g., the B-finger of TFIIB inserts into the polymerase active site to complete escape commitment [81].
*E_I_*	Reactions that change the microenvironment when the EEC begins elongation; enhancer-promoter proximity [75,81].	Transcript slippage and backtracking [81].
*E_I_^P^*		Transcript synthesis; recovery of the elongation competency of arrested Pol II via TFIIS; release of paused Pol II to enter productive elongation [81].

Second, we defined 6 events, a detailed description of which is presented in Fig. S1. *E*_P_ refers to a series of *I_E_* and *E_I_* transitions plus the ending transition *E_I_^P^*, resulting in synthesis of mRNA transcripts. *E*_S1_ comprises a series of *I_E_* and *E_I_* plus *I_P_*, responsible for disassembly of the scaffold complex. *E*_S2_ consists of a series of *A_F_* and *F_A_* plus *A_I_*, enabling assembly of the PIC. *E*_S3_ comprises a series of *A_F_*, *F_C_*, *C_F_*, and *F_A_*, maintaining the inaccessibility of GTFs to the core promoter and delaying the entry into *E*_S2_. As the beginning event of the entire transcriptional process, *E*_begin_ comprises a series of *C_F_* and *F_C_* plus *F_A_*, contributing to chromatin opening; as the ending event, *E*_end_ is composed of a series of *A_F_* and *F_A_* plus *F_C_*, resulting in termination of transcription. Notably, each event is not fixed in composition but amenable to modulation; *E*_begin_ and *E*_end_ are governed by the initial and final states in set *C*, respectively. A single transcriptional burst undergoes 2 stages: The inactive phase refers to no mRNA production, including *E*_S1_, *E*_S2_, and *E*_S3_, while the active phase corresponds to mRNA synthesis, comprising a sequence of *E*_P_.

Last, we developed an event-based model for transcription (Fig. 1B). *E*_begin_ first appears, and then *E*_S2_ follows, or *E*_S3_ occurs once or repetitively until *E*_S2_ arises. Subsequently, different paths lead to the (repetitive) occurrence of *E*_P_, with a burst of mRNAs generated. After bursting, *E*_S1_ emerges, then *E*_S2_ appears with or without *E*_S3_ preceding, and a new burst may ensue. Notably, there may exist a refractory period in *E*_S1_ where immediate reactivation of transcription is prohibited. The above process repeats until *E*_S1_ and *E*_end_ appear successively, with the gene returning to the silent state. Thus, the transcription is characterized by a sequence of ordered events. The process of mRNA splicing, nuclear export, and degradation of mature mRNA is simplified as an effective Poisson process with rate constant 1/*τ*_m_. *τ*_m_ is set to 5 min unless specified otherwise. The biochemical master equation describing mRNA production is presented in Text S1.

Given the stochasticity in transcription, we focused on the distribution function *f_i_* of the duration of event *i* (*i* = P, S1, S2, S3) in steady state with the mean *τ*_i_ (Text S2 and Figs. S2 and S3). The probability of repeated occurrence of *E*_P_ (*E*_S3_) is *p*_1_ (*p*_2_) (Fig. S4). In general, *f*_P_, *f*_S1_, *f*_S2_, and *f*_S3_ are primarily controlled by Pol II-dependent reactions, disassembly of the scaffold complex, GTF-dependent reactions, and nucleosome-associated reactions near the core promoter, respectively. *f*_i_ is an exponential or a unimodal function in most cases [11,44]. *τ*_i_ is mainly determined by rate-limiting steps, whose durations in a burst are the main constituent of the burst period. *p*_1_ is elevated via accelerating such reactions as recruitment, phosphorylation, and release of Pol II, which promote the transition of *A* → *I* → *E* → *P*, while *p*_2_ is boosted by accelerating reactions that promote *I* → *A* → *F* → *C*.

Once *f*_i_, *p*_1_ and *p*_2_ were given or derived from experimental data, numerical simulation was performed to depict mRNA production using the Gillespie algorithm (Text S3). The default setting of *f*_i_, *p*_1_, and *p*_2_ is denoted as (*f*_iU_, *p*_1U_, *p*_2U_), corresponding to the situation without regulators binding to cognate sites, and is determined by the gene specificity [45] and local environment; it changes into (*f*_iB_, *p*_1B_, *p*_2B_) with bound regulators (Fig. 1C). At various regulator concentrations, its rate of occupancy at the regulatory site varies between 0 and 1, and the resulting (*f*_i_, *p*_1_, *p*_2_) lies between (*f*_iU_, *p*_1U_, *p*_2U_) and (*f*_iB_, *p*_1B_, *p*_2B_). That is, the regulation of transcription is mapped to changes in *f*_i_, *p*_1_, and *p*_2._ We found that the main conclusions do not rely heavily on the concrete form of *f*_i_ provided that it is exponential or unimodal (Figs. S5 and S6). All the results presented here are based on the exponential distributions. Since an exponential distribution is determined by its mean value, the roles for regulators in gene transcription are classified on the basis of their influence on *τ*_i_, *p*_1_, and *p*_2_. There exist 255 (2^8^ − 1) regulatory modes, and Fig. 1D illuminates 8 basic modes, under each of which regulators affect *τ*_P_, *τ*_S1_, *τ*_S2_, *τ*_S3_, *p*_1_ (through *J*_1_^+^ or *J*_1_^−^), or *p*_2_ (through *J*_2_^+^ or *J*_2_^−^) alone.

Compared with previous models where independent quantities characterized experimental observables, the current model focuses on a sequence of ordered events and is more concerned with the impact of biochemical reactions on the event durations and transitions. Thus, the quantities such as the transcription rate constant (1/*τ*_P_), the duration of the active phase [*τ*_A_ = *τ*_P_/(1 − *p*_1_)] and of the inactive phase (*τ*_I_ = *τ*_S_/*p*_1_) are all correlated (see below), which will have important implications as shown later.

### Regulatory modes promoting transcriptional sensitivity and specificity

Gene transcription is mediated by a multitude of regulatory factors, such as TFs and cofactors. Without loss of generality, they are assumed to function by binding their cognate sites to affect biochemical reactions. For the general multibinding site scenario, the occupancy rate of regulators can be expressed as $\frac{{\left[R\right]}^{n_{H}}}{{\left[R\right]}^{n_{H}}+K_{d}^{n_{H}}}$, where [*R*] denotes the regulator concentration, *K*_d_ is a constant, and Hill coefficient *n*_H_ could be a noninteger. As shown in Texts S4 and S5, if [*R*^′^] and *K*^′^are separately substituted for ${\left[R\right]}^{n_{H}}$ and $K_{d}^{n_{H}}$, either parts of original analytical expressions are identical to those in the case of *n*_H_ = 1, or relationships between specific quantities can be preserved. Consequently, the occupancy rate can simply be written as$\frac{\left[R\right]}{K_{d}+\left[R\right]}$, corresponding to the simplest single-binding site case, for the purpose of simplifying the exploration of gene regulation.

**Fig. 2.**
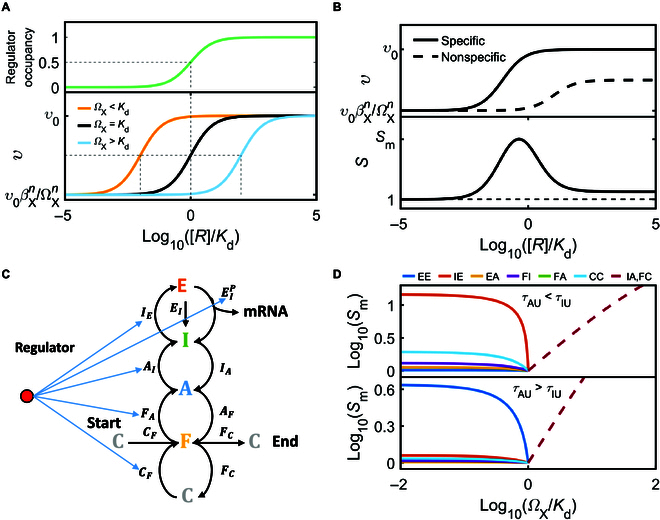
Sensitivity to changes in regulator concentration ([*R*]) and resistance against crosstalk. (A) Schematic of the mean regulator occupancy rate (top) and steady-state transcription rate (bottom) versus the normalized concentration of regulators. *K*_d_ denotes the equilibrium dissociation constant for regulator binding, and *K*_d_/*Ω*_X_ reflects the transcriptional sensitivity. (B) Schematic of the mean transcription rate (top) and specificity *S* (bottom) versus [*R*]/*K*_d_. The equilibrium dissociation constant is *K*_d_ and 100*K*_d_, respectively, for cognate and nontarget binding. (C) Regulation of *E_I_^P^*, *I_E_*, *A_I_*, *F_A_*, or *C_F_* promotes the gene’s sensitivity to the regulator when its binding facilitates state transitions. (D) *S*_m_ versus *Ω*_X_/*K*_d_ under different regulatory modes. The default parameters are *τ*_PU_ = 1 min, *τ*_S1U_ = 5 min, *τ*_S2U_ =10 min, *τ*_S3U_ = 20 min, *p*_2U_ = 0.5, and *p*_1U_ = 0.5, leading to *τ*_AU_ < *τ*_IU_ (top); *τ*_PU_ = 10 min, *τ*_S1U_ = 2 min, *τ*_S2U_ =5 min, *τ*_S3U_ = 10 min, *p*_2U_ = 0.5, and *p*_1U_ = 0.9, leading to *τ*_AU_ > *τ*_IU_ (bottom). *τ*_PB_, *τ*_S1B_, *τ*_S2B_, *τ*_S3B_, *p*_1B_, and *p*_2B_ are decided by Ω_X_/*K*_d_ and regulatory modes (see Table 3).

As mentioned above, bursting parameters can be modulated to various extents under 255 regulatory modes. In mode *X*, the mean transcription rate 𝜐 in steady state is approximated as

$$\upsilon \approx \upsilon_{0}\frac{\beta_{X}^{n}+{\left[R\right]}^{n}}{\Omega_{X}^{n}+{\left[R\right]}^{n}},$$ (1)

where Ω_X_ refers to [*R*] at which *υ* is halfway between its maximum and minimum, *β*_X_ is associated with the basal transcription without bound regulators, and *n* is a fitting number (see Text S6 for details). *K*_d_/*Ω*_X_ reflects the sensitivity of transcription to changes in [*R*] (Fig. 2A); high sensitivity allows for efficient regulation of transcription [46]. *F*_υ_ = *υ*([*R*] = ∞)/*υ*([*R*] = 0) = (*Ω*_x_/*β*_x_)*^n^* measures how adjustable the mean transcription rate is.

Regulators actually bind to both cognate and nontarget sites, and the latter may also induce mRNA production. To quantify the difference between the 2 scenarios, we introduced the specificity *S* defined as the ratio of the average expression resulting from specific binding of regulators to that resulting from nonspecific binding. *S* is a unimodal function of [*R*] for nonzero *β* (Fig. 2B); its maximum *S*_m_ approximately equals *F*_υ_, given that cognate binding has a much higher affinity than nontarget binding (see Text S6 for details). Collectively, *K*_d_/*Ω*_X_ and *S*_m_ provide crucial information about transcriptional regulation.

Among 8 basic regulatory modes, *Ω*_X_ < *K*_d_ is realizable for *X* = MEE/EA/FI/CC/FA/IE, whereas *Ω*_X_ > *K*_d_ for *X* = MIA/FC (Fig. 1D and Table 3). In the former, the transcription is activated by reducing *τ*_P_, *τ*_S1_, *τ*_S2_, *τ*_S3_, *p*_2_ (via *J*_2_^−^) and increasing *p*_1_ (via *J*_1_^+^), which are achieved mainly by accelerating *E_I_^P^* and *I_E_*, *I_A_* and *E_I_*, *A_I_* and *F_A_*, *F_A_* and *C_F_*, *F_A_* and *A_I_*, and *I_E_* and *E_I_^P^*, respectively. In the latter, transcription is activated by increasing *p*_1_ (via *J*_1_^−^) and reducing *p*_2_ (via *J*_2_^+^), which can be acquired by slowing down *I_A_* and *E_I_* and *A_F_* and *F_C_*, respectively. Therefore, with *Ω*_X_ < *K*_d_, the transcriptional rate reaches its maximum before the regulator occupancy saturates, while the regulator binding promotes transcription by speeding up some state transitions, which involve the recruitment of TFs, enzymes, and Pol II and are part of *E_I_^P^*, *I_E_*, *A_I_*, *F_A_*, or *C_F_*. By contrast, with *Ω*_X_ > *K*_d_, the regulator binding contributes to transcription by slowing down some state transitions, which are engaged in *E_I_*, *I_A_*, *A_F_*, or *F_C_*. Notably, most reactions involved in *E_I_*, *I_A_*, *A_F_*, and *F_C_* are irrespective of regulator binding, like disintegration of the enhancer-promoter looping and scaffold complex. Together, the regulation of *E_I_^P^*, *I_E_*, *A_I_*, *F_A_*, or *C_F_* may be engaged more frequently to gain high transcriptional sensitivity (Fig. 2C).

**Table 3.** Gene regulatory function.

Mode	Changed parameter	Parameter change	*υ*_max_	*β*	Ω
MEE	*τ*_P_	*τ*_PB_ = *ε*_EE_*τ*_PU_ *ε*_EE_ < 1	$\frac{1}{1-p_{1}}\frac{1}{\varepsilon_{\mathrm{EE}}\tau_{\mathrm{AU}}+\tau_{I}}$	*ε*_EE_ *K*_d_	$\frac{\tau_{\mathrm{AU}}+\tau_{I}}{\tau_{\mathrm{AU}}+\frac{\tau_{I}}{\varepsilon_{\mathrm{EE}}}}K_{d}<K_{d}$
MEA	*τ*_S1_	*τ*_S1B_ = *ε*_EA_*τ*_S1U_ *ε*_EA_ < 1	$\frac{1}{1-p_{1}}\frac{1}{\tau_{A}+\varepsilon_{\mathrm{EA}}\frac{1}{p_{1}}\left(\tau_{S1U}+\tau_{S2}\right)+\tau_{I2}}$	*ε*_EA_ *K*_d_	$\frac{\varepsilon_{\mathrm{EA}}\tau_{I1U}+\varepsilon_{\mathrm{EA}}\left(\tau_{A}+\tau_{I2}\right)}{\varepsilon_{\mathrm{EA}}\tau_{I1U}+\left(\tau_{A}+\tau_{I2}\right)}K_{d}<K_{d}$
MFI	*τ*_S2_	*τ*_S2B_ = *ε*_FI_*τ*_S2U_ *ε*_FI_ < 1	$\frac{1}{1-p_{1}}\frac{1}{\tau_{A}+\varepsilon_{\mathrm{FI}}\frac{1}{p_{1}}\left(\tau_{S1}+\tau_{S2U}\right)+\tau_{I2}}$	*ε*_FI_ *K*_d_	$\frac{\varepsilon_{\mathrm{FI}}\tau_{I1U}+\varepsilon_{\mathrm{FI}}\left(\tau_{A}+\tau_{I2}\right)}{\varepsilon_{\mathrm{FI}}\tau_{I1U}+\left(\tau_{A}+\tau_{I2}\right)}K_{d}<K_{d}$
MCC	*τ*_S3_	*τ*_S3B_ = *ε*_CC_*τ*_S3U_ *ε*_CC_ < 1	$\frac{1}{1-p_{1}}\frac{1}{\tau_{A}+\tau_{I1}+\varepsilon_{\mathrm{CC}}\tau_{I2U}}$	*ε*_CC_ *K*_d_	$\frac{\varepsilon_{\mathrm{CC}}\left(\tau_{A}+\tau_{I1}\right)+\varepsilon_{\mathrm{CC}}\tau_{I2U}}{\tau_{A}+\tau_{I1}+\varepsilon_{\mathrm{CC}}\tau_{I2U}}K_{d}<K_{d}$
MIE	*p*_1_ via *J*_1_^+^	*p*_1B_ = *ε*_IE_*p*_1U_ *ε*_IE_ > 1	$\frac{1}{1-p_{1U}}\frac{1}{\tau_{\mathrm{AU}}+\frac{1-\varepsilon_{\mathrm{IE}}p_{1U}}{\varepsilon_{\mathrm{IE}}\left(1-p_{1U}\right)}\tau_{\mathrm{IU}}}$	$\frac{1-\varepsilon_{\mathrm{IE}}p_{1U}}{\varepsilon_{\mathrm{IE}}\left(1-p_{1U}\right)}K_{d}$	$\frac{\tau_{\mathrm{AU}}+\tau_{\mathrm{IU}}}{\frac{\varepsilon_{\mathrm{IE}}\left(1-p_{1U}\right)}{1-\varepsilon_{\mathrm{IE}}p_{1U}}\tau_{\mathrm{AU}}+\tau_{\mathrm{IU}}}K_{d}<K_{d}$
MIA	*p*_1_ via *J*_1_^−^	*p*_1B_ = *ε*_IA_*p*_1U_ *ε*_IA_ > 1	$\frac{1}{1-p_{1U}}\frac{1}{\tau_{\mathrm{AU}}+\frac{1-\varepsilon_{\mathrm{IA}}p_{1U}}{\varepsilon_{\mathrm{IA}}\left(1-p_{1U}\right)}\tau_{\mathrm{IU}}}$	*K*_d_	$\frac{\tau_{\mathrm{AU}}+\tau_{\mathrm{IU}}}{\tau_{\mathrm{AU}}+\frac{1-\varepsilon_{\mathrm{IA}}p_{1U}}{\varepsilon_{\mathrm{IA}}\left(1-p_{1U}\right)}\tau_{\mathrm{IU}}}K_{d}>K_{d}$
MFC	*p*_2_ via *J*_2_^+^	*p*_2B_ = *ε*_FC_*p*_2U_ *ε*_FC_ <1	$\frac{1}{1-p_{1}}\frac{1}{\tau_{A}+\tau_{I1}+\frac{\varepsilon_{\mathrm{FC}}\left(1-p_{2U}\right)}{1-\varepsilon_{\mathrm{FC}}p_{2U}}\tau_{I2U}}$	*K*_d_	$\frac{\tau_{A}+\tau_{I1}+\tau_{I2U}}{\tau_{A}+\tau_{I1}+\frac{\varepsilon_{\mathrm{FC}}\left(1-p_{2U}\right)}{1-\varepsilon_{\mathrm{FC}}p_{2U}}\tau_{I2U}}K_{d}>K_{d}$
MFA	*p*_2_ via *J*_2_^−^	*p*_2B_ = *ε*_FA_*p*_2U_ *ε*_FA_ <1	$\frac{1}{1-p_{1}}\frac{1}{\tau_{A}+\tau_{I1}+\frac{\varepsilon_{\mathrm{FA}}\left(1-p_{2U}\right)}{1-\varepsilon_{\mathrm{FA}}p_{2U}}\tau_{I2U}}$	$\frac{\varepsilon_{\mathrm{FA}}\left(1-p_{2U}\right)}{1-\varepsilon_{\mathrm{FA}}p_{2U}}K_{d}$	$\frac{\tau_{A}+\tau_{I1}+\tau_{I2U}}{\left(\tau_{A}+\tau_{I1}\right)\frac{1-\varepsilon_{\mathrm{FA}}p_{2U}}{\varepsilon_{\mathrm{FA}}\left(1-p_{2U}\right)}+\tau_{I2U}}K_{d}<K_{d}$

Note: *υ* = *υ*_max_(*β* + [*R*])/(*Ω* + [*R*]), *τ*_A_ = *τ*_P_/(1 − *p*_1_),*τ*_I_ = *τ*_I1_ + *τ*_I2_, *τ*_I1_ = (*τ*_S1_ + *τ*_S2_)/*p*_1_, *τ*_I2_ = *p*_2_*τ*_S3_/[*p*_1_(1 − *p*_2_)]. “B” and “U” denote the cases with and without bound regulators, respectively. No “U” emerges if regulator binding has no influence. *ε*_X_ (X = EE, EA, FI, CC, IE, IA, FC, FA) is the ratio of the corresponding parameter with bound regulators to that without bound regulators.

The type of regulatory mode determines the trend in *S*_m_ changing with *Ω*_X_. For *Ω*_X_ < *K*_d_, *S*_m_ drops with increasing *Ω*_X_/*K*_d_ (Fig. 2D); *S*_m_ varies sharply around *Ω*_X_/*K*_d_ = 1 under MIE (MEE) for *τ*_AU_ < *τ*_IU_ (*τ*_AU_ > *τ*_IU_). The maximum of *S*_m_ is achieved via MIE for *τ*_AU_ < *τ*_IU_ and via MEE otherwise, suggesting that regulating *E_I_^P^* or *I_E_* is a desirable mode. For *Ω*_X_ > *K*_d_, *S*_m_ rises linearly with increasing *Ω*_X_/*K*_d_ (on a log–log scale); obtaining a large *S*_m_ through MIA/FC requires that the binding sites should always be occupied, which is biologically implausible.

According to the event model, we directly infer that the regulation of *E_I_^P^*, *I_E_*, *A_I_*, *F_C_*, or *C_F_* can enhance the transcriptional sensitivity while promoting mRNA synthesis. The mode modulating *E_I_^P^* and/or *I_E_* has a marked role in enlarging the transcriptional adjustability and reducing the crosstalk effects resulting from noncognate regulator binding.

### Major regulatory modes underlying the observed transcriptional noise

Transcription is a complex stochastic process; for simplicity, we only explored intrinsic noise in transcription, neglecting variations in parameters between cells or across time (i.e., extrinsic noise). The relationship between the mean (<*m*>) and variance (*σ*_m_^2^) of mRNA copy number or Fano factor (*F* = *σ*_m_^2^/<*m*>) is a key representation of transcriptional regulation. <*m*>, *σ*_m_^2^, and *F* in steady state take the following forms (see Text S7 for details):

$$\langle m\rangle =\frac{\tau_{m}}{\tau_{P}+\frac{1-p_{1}}{p_{1}}\tau_{S}},$$ (2)

$$\sigma_{m}^{2}\approx \langle m\rangle +{\langle m\rangle }^{2}\frac{\left(\frac{\tau_{I}}{\tau_{m}}+\frac{\tau_{A}}{\tau_{m}}\right)\frac{\tau_{S}}{\tau_{m}}-\frac{\tau_{A}\tau_{I}}{\tau_{m}^{2}}}{\frac{\tau_{I}}{\tau_{m}}+\frac{\tau_{A}}{\tau_{m}}+\frac{\tau_{A}\tau_{I}}{\tau_{m}^{2}}},$$ (3)

$$F\approx 1+\frac{b}{c+d}\frac{d\left(c+d\right)\left(b-1\right)/b-\mathrm{cd}}{c+d+\mathrm{cd}},$$ (4)

where *τ*_m_ is the lifetime of mRNA, *τ*_A_ (*τ*_I_) is the mean duration of the active (inactive) phase, and *τ*_S_ is the mean interval between *E*_S1_ and *E*_S2_: *τ*_A_ = *τ*_P_/(1 − *p*_1_), *τ*_I_ = *τ*_S_/*p*_1_, and *τ*_S_ = *τ*_S1_ + *τ*_S2_ + *p*_2_*τ*_S3_/(1 − *p*_2_). The Fano factor is governed by the mean burst size *b* [the number of mRNAs per burst; *b* = 1/(1 − *p*_1_)], *c* = *τ*_A_ /*τ*_m_, and *d* = *τ*_I_ /*τ*_m_. A small burst size, a plateau-like bursting profile, or a short inactive phase can contribute to reducing *F.*

**Fig. 3.**
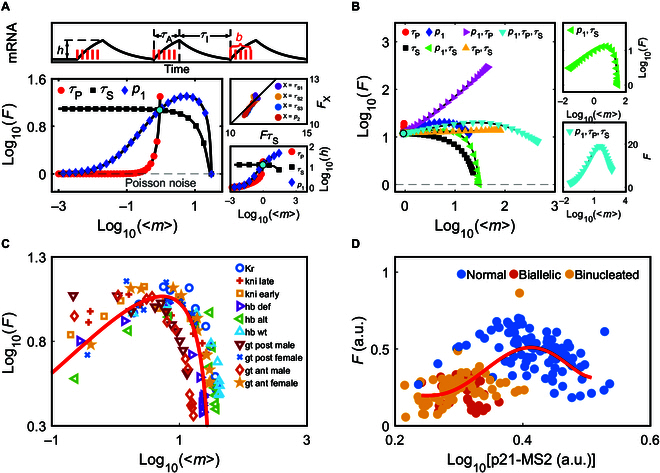
Relationships between the mean mRNA number <*m*> and Fano factor *F* under various regulatory modes*.* Time is in units of minutes. (A and B) Solid lines are from analytic expressions (Eqs. G21 to G23 in Text S7), while symbols label numerical results. (A) *F* versus <*m*> for different parameter values. The dashed line represents *F* = 1 (Poisson noise). The default parameters without bound regulators are *p*_1U_ = 0.95 and *τ*_PU_ = 0.165, and *τ*_SU_ is assumed to be exponentially distributed with an average of 100 min (cyan dot). On each curve, only one parameter (*p*_1_, *τ*_P_, or *τ*_S_) varies, while the others are fixed at default values. The top panel depicts the sketch of transcriptional bursts (red bars represent Pol II entering productive elongation). The right bottom panel shows the corresponding burst height *h* versus *m*, while the right top one shows *F*τs versus *F*_X_ (*X* = *τ*_S1_, *τ*_S2_, *τ*_S3_, *p*_2_) with *τ*_S1U_ = 10, *τ*_S2U_ = 30, *τ*_S3U_ = 60, and *p*_2U_ = 0.5. (B) *F* versus <*m*> in different regulatory modes. Regulators affect the transcription by changing the following parameters, while the others remain fixed (*p*_1U_ = 0.95, *τ*_PU_ = 0.165, *τ*_SU_ = 100): *τ*_P_ (red circle; *τ*_PB_ = 0.01), *τ*_S_ (black square; *τ*_SB_ = 1), *p*_1_ (blue diamond; *p*_1B_ = 0.999), *τ*_P_ and *p*_1_ (pink rightward triangle; *τ*_PB_ = 0.01 and *p*_1B_ = 0.999), *p*_1_ and *τ*_S_ (green leftward triangle; *p*_1B_ = 0.999 and *τ*_SB_ = 1), *τ*_P_ and *τ*_S_ (yellow upward triangle; *τ*_PB_ = 0.01and *τ*_SB_ = 1); and *τ*_P_, *p*_1_, and *τ*_S_ (cyan downward triangle; *τ*_PB_ = 0.01, *p*_1B_ = 0.999, and *τ*_SB_ = 1). Each data point represents an average over 10 thousand trajectories. Here, the regulator binding promotes transcription. Two right panels show the cases where both *p*_1_ and *τ*_S_ are regulated with *τ*_P_ fixed (*p*_1U_ = 0.7, *τ*_SU_ = 500, *p*_1B_ = 0.999, *τ*_SB_ = 1, *τ*_P_ = 0.165; top), or *p*_1_, *τ*_S_, and *τ*_P_ are regulated (*τ*_PB_ = 0.01, *p*_1B_ = 0.999, *τ*_SB_ = 1, *τ*_PU_ = 0.2, *p*_1U_ = 0.5, τ_SU_ = 100; bottom). (C and D) Experimental data on gap genes in early *Drosophila* embryos [47] (C) and on *CDKN1A* (measured by p21-MS2 signals) in MCF7 cells [49] (D). The red curve is a fit to all data with the event model. a.u., arbitrary units.

Figure 3A displays how *F* varies with <*m*> over a wide range of parameter values. For each curve, only one of the 6 parameters is altered, while the others are fixed at default values; we also performed simulations by directly setting *τ*_S_ rather than *τ*_S1_, *τ*_S2_, *τ*_S3_, and *p*_2_ and assumed *τ*_S_ to obey an exponential distribution. When *y* is varied, the corresponding *F* is denoted as *F*_y_. As *Fτ*_s_ differs slightly from *F*_x_ (*x* = *τ*_S1_, *τ*_S2_, *τ*_S3_, or *p*_2_) (Fig. 3A, inset), only the curve for *Fτ*_s_ is presented. With *τ*_P_ regulated alone, the mean height of burst profiles {*h* ≈ *τ*_m_(1 − e*^−c^*)(1 − e*^−d^*)/[*τ*_P_(1 − e^*−*c−d^)] for *τ*_P_ < *τ*_m_ and *h* ≈ 1 otherwise}, rather than burst size, varies prominently. At small *τ*_P_, *F* rises sharply with increasing <*m*> due to little degradation of mRNA within a short active period. At large *τ*_P_, the transcription rate is small such that a nascent mRNA has a high probability of being degraded before new mRNA production, and the active phase is much longer than the inactive phase, i.e., the transcription is nearly a Poisson process (*F* = 1). With *τ*_S_ modulated alone, *b* and *τ*_A_ show little change, whereas *τ*_I_ varies markedly, such that *F* drops toward one with increasing <*m*>*. F* varies slightly at small <*m*> but drops markedly at large <*m*> due to a continuous reduction in

$$\frac{\mathrm{dF}}{d\langle m\rangle }\approx \frac{{\left(1-p_{1}\right)}^{2}\frac{\tau_{m}^{3}}{\tau_{P}^{3}}\left[1+\frac{\tau_{m}}{\tau_{P}}\right]}{{\left[m-\left(1-p_{1}\right)\frac{\tau_{m}^{2}}{\tau_{P}^{2}}-\frac{\tau_{m}}{\tau_{P}}\right]}^{2}}-1.$$ (5)

With *p*_1_ altered alone, *b* rises with increasing <*m*>, and *h* rises toward saturation; thus, *F* first rises because of an increase in *b* and then drops because of saturation in *h* and plateau-like bursting. Strikingly, all numerical results agree perfectly with the analytical expressions.

The dependence of *F* on <*m*> is more complicated when 2 or more parameters vary concurrently. Notably, the curves for *F*τ_P_ and *F*τ_S_ constitute the boundaries, and the others lie in between and exhibit unique features (Fig. 3B and Fig. S7). Providing that the regulator binding promotes transcription, *F* rises markedly at small <*m*> together with a notable change in *h* or varies slightly when *h* changes little. If *F* falls at intermediate or large <*m*>, then at least *τ*_S_ or *p*_1_ is regulated, and <*m*> tends toward *τ*_m_/*τ*_P_ when *F* approaches 1; specifically, *F* drops rapidly when *τ*_P_ is sufficiently large or changes little*.* Together, the regulation of *τ*_P_ or *p*_1_ is indispensable for a rise in *F*, and its rapid rise is accompanied by sharp bursting; the regulation of *τ*_S_ or *p*_1_ is essential to a drop in *F*, and its fast fall is associated with plateau-like bursting. Similarly, the numerical and analytic results match exactly.

Indeed, the fitting curves to experimental data resemble the typical curves above. Activated by the Bicoid (Bcd) protein, major gap genes in early *Drosophila* embryo are expressed at distinct levels along the anterior–posterior axis of its body due to the Bcd concentration gradient [47], sharing a similar trend in *F* versus <*m*> (i.e., *F* first rises and then drops toward 1 with increasing <*m*>) (Fig. 3C; see also the right top panel in Fig. 3B). All the data points can be fitted by a single curve with the event model (see parameter inference in Text S4). For each gene, *F* is greater than 3.8 at small <*m*> and falls rapidly after the maximum, indicating that *τ*_P_ is nearly consistent across body locations. With *τ*_P_ fixed, there should exist the upper bounds, <*m*> < *τ*_m_/*τ*_P_ and *F <* 1 + *τ*_m_/*τ*_P_ (see Text S7 for details). As these genes display similar limits in <*m*> and *F* and have similar *τ*_m_ values [48], *τ*_P_ (and the frequency of transcription initiation) should also be comparable among them, which implies that variations in Bcd concentration have a minor impact on the major rate-limiting steps in Pol II recruitment and promoter-proximal pausing. The increase in Bcd concentration may primarily facilitate the activation of Pol II and its entry into productive elongation.

Another example involves the expression of *CDKN1A* activated by the tumor suppressor p53 in MCF7 cell lines [49]. *F* varies slightly at small <*m*>, rises to a peak, and then drops gradually (Fig. 3D); to fit the data with the event model, *p*_1_, *τ*_S_, and *τ*_P_ vary concurrently with small *p*_1U_ and *τ*_P_ (see also the right bottom panel in Fig. 3B). At small *p*_1U_, the gene state tends to leave set *E* via *E_I_* rather than *E_I_^P^* or leave set *I* via *I_A_* rather than *I_E_*, associated with slow reactions in recruiting, stabilizing, or releasing Pol II. At small *τ*_P_, fast reactions are required before Pol II enters productive elongation. Thus, p53 may prefer to regulate *CDKN1A* expression via speeding up *E_I_^P^* through cellular signaling.

Further analysis of other experimental data reveals the diversity in transcriptional regulation. When the *ctgf* expression is mediated by transforming growth factor-β (TGF-β), the transcription rate rises with increasing the TGF-β concentration, and *τ*_A_ is nearly fixed [50], which requires that *τ*_P_ is inversely proportional to *b.* This further implies that the likelihood of Pol II entering productive elongation is prominently higher than its probability of escaping from the core promoter, i.e., the modulation lies in *E_I_^P^* rather than *I_E_*. When glucagon-like peptide 1 (GLP-1)/Notch induces *sygl-*1 expression in *Caenorhabditis elegans*, the burst size is nearly irrelevant to the concentration of GLP-1/Notch ligand [51], i.e., *τ*_P_ varies markedly but *b* (*p*_1_) is fixed, implying that the early elongation of Pol II until promoter-proximal pausing can be modulated. Obviously, different regulations of *τ*_P_ and *p*_1_ are gene-specific, reflecting which steps are rate-limiting and which reactions are being adjusted in *E*_P_.

Together, the event model enables the deduction of transcriptional modulatory mechanisms. The results above suggest that the changing trend of burst profiles is influenced by the adjustable range of Pol II-dependent reaction rates (e.g., recruitment and promoter-proximal elongation of Pol II). In the 3 limiting cases where the transcription occurs with fixed *τ*_A_, *b*, or *τ*_P_, the burst height, burst duration, and area under the curve are modulated, respectively.

### Global constraints on transcriptional bursting

**Fig. 4.**
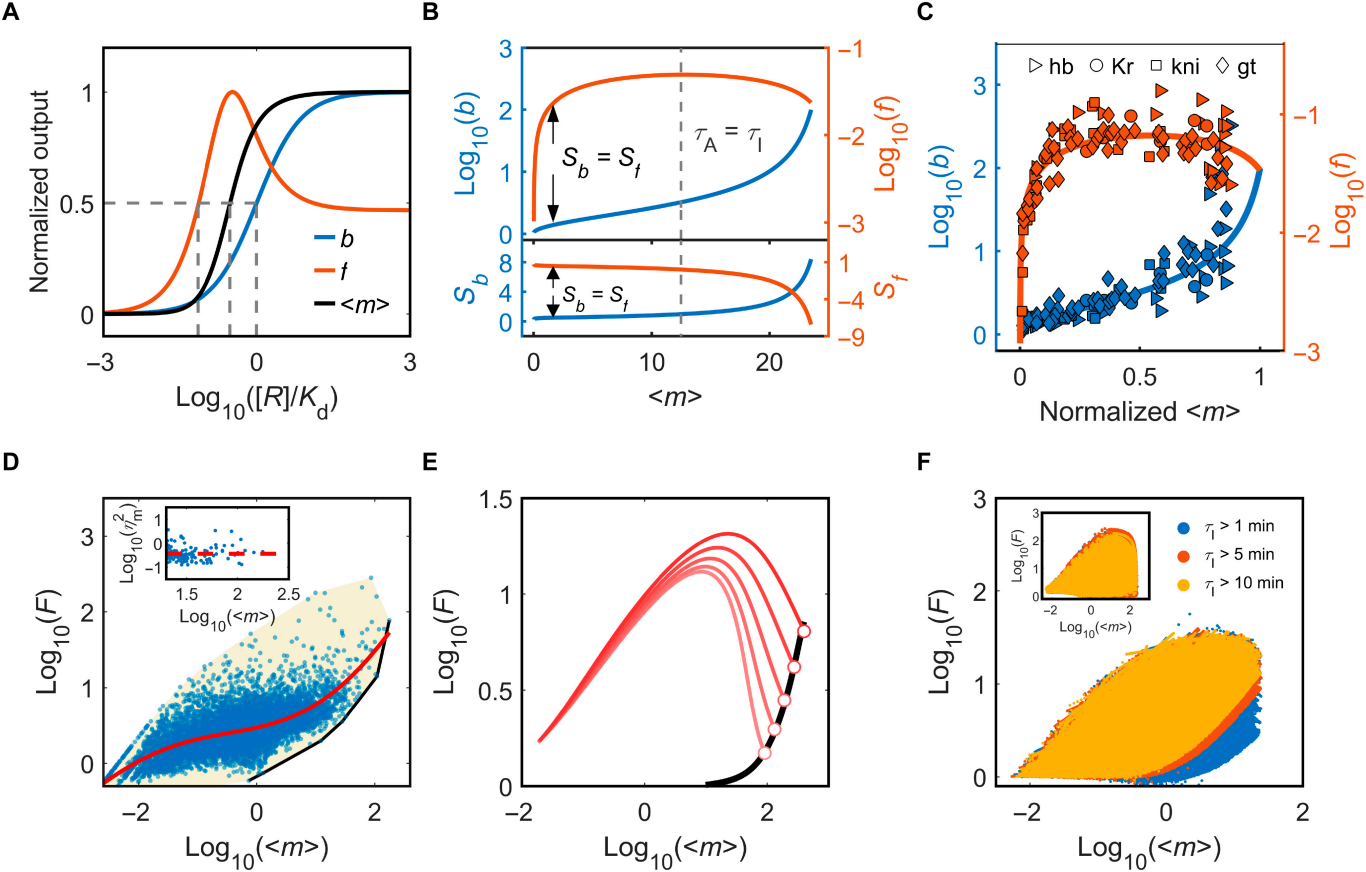
Modulation of transcriptional bursting. Time is in units of minutes. (A) Mean mRNA count <*m*>, burst size *b*, and burst frequency *f*, normalized by their respective maximum values, versus the normalized regulator concentration [*R*]. The regulator binding affects *p*_1_ and *τ*_S_; *τ*_P_ = 0.2, *p*_1B_ = 0.995, *p*_1U_ = 0.5, *τ*_SU_ = 250, *τ*_SB_ = 5 and *n* = 1. (B) Burst size and frequency versus the mean mRNA number (top); *S*_b_ and *S*_f_ versus <*m*> (bottom). The dashed line marks the case of *τ*_I_ = *τ*_A_. The black arrows point to <*m*> with *S*_f_ = *S*_b_. The parameters are the same as in (A). The curves in (A) and (B) are plotted on the basis of analytic expressions. (C) A fit to the experimental data (symbols) in [47] using the event model with *τ*_P_ *=* 0.139, *p*_1U_ = 0.401, *p*_1B_ = 0.995, *τ*_SU_ = 498, and *τ*_SB_ = 0.694 (solid line). (D) Experimental data on the mean expression level and Fano factor in [52]. Each point is from an individual gene with the shadow showing the range. The red fitting curve is obtained by Gaussian regression. The black curve shows the lower bound on Fano factor at large <*m*>. The inset shows $\eta_{m}^{2}=\sigma_{m}^{2}/$<*m*>^2^ versus <*m*>, with the red dashed line representing the mean. (E) The red lines show Fano factor versus <*m*>, and the black line shows the lower limit of Fano factor at large <*m*> with *τ*_S_ ≥ 1. The default parameters are the same as in (A). (F) Fano factor versus <*m*> with the event model or telegraph model (inset). Ten thousand sets of bursting parameters are used in each panel, where the duration of a burst varies between 1 and 1,000 min, the transcription rate is from 0.1 to 100/min and *τ*_A_ < *τ*_I_. The refractory period is set to 1 (blue), 5 (red), or 10 min (yellow).

The results above unravel an important regulatory mode involving the concurrent modulation of *p*_1_ and *τ*_S_. Under this mode, the mean mRNA number (<*m*> *= υτ*_m_) and burst size *b* rise toward saturation with increasing [*R*], while the burst frequency *f* first rises to a peak and then drops toward saturation, corresponding to *τ*_A_ < *τ*_I_ and *τ*_A_ > *τ*_I_, respectively (Fig. 4A). *Ω*_m_, *Ω*_b_, and *Ω*_f_ refer to the operating points where *m*, *b*, and *f* reach half of their maximum values, respectively. Because *Ω*_m_ < *K*_d_, *Ω*_b_ ≈ *K*_d_ when *E_I_^P^*, *I_E_*, *A_I_*, *F_A_*, or *C_F_* is modulated, and <*m*> = *bfτ*_m_, *Ω*_f_ *< Ω*_m_ *< Ω*_b_ generally holds true, and thus *f*, <*m*>, and *b* reach their respective maximum values successively (see Text S4 and Fig. S8 for details). Furthermore, *f* is sensitive to changes in <*m*> at low expression, while *b* is insensitive, and vice versa at high expression (Fig. 4B, top)*_._*

We also used $S_{b}=\frac{d\ln b}{d\ln m}$ and $S_{f}=\frac{d\ln f}{d\ln m}$ (*S*_b_ + *S*_f_ = 1; *S*_b_ and *S*_f_ > 0 for *τ*_A_ < *τ*_I_), i.e., their sensitivity to changes in mRNA number, to dissect the contribution of size and frequency modulation to mRNA production. *S*_f_ and *S*_b_ drop and rise respectively with increasing <*m*> (Fig. 4B, bottom), indicating that the modulation of burst frequency and size predominates in distinct regimes and a combination of both mechanisms emerges over intermediate ranges. A similar relationship between *f*(*b*) and <*m*> was observed across gap genes in early *Drosophila* embryos [47] (Fig. 4C), where their expression levels are graded along the anterior–posterior axis, responsible for body segmentation.

With high-throughput experimental data available, we calculated the mean mRNA numbers and Fano factors for 9196 genes in fibroblasts [52]; to exclude the influence of extrinsic noise, we preprocessed the data using the method in [53]. Each mRNA count is normalized by dividing it by the total count in each sample and scaled by the median count across all samples. Each data point in Fig. 4D denotes <*m*> and *F* for one gene, and the red curve is a fitted trendline for all data. There exist lower bounds on Fano factor and burst size at each expression level; meanwhile, $\eta_{m}^{2}=\sigma_{m}^{2}/$<*m*>^2^ fluctuates around the mean for large <*m*> (Fig. 4D, inset), while the total noise intensity in the original data from [52] remains nearly constant at high expression levels [54]. The lower bound on *F* is nearly irrelevant to <*m*> at low expression but rises prominently with increasing <*m*> at high expression. That is, higher expression is accompanied by increased burstiness. This phenomenon has been widely observed in mammalian cells [44,55–58], fly embryos [59–61], and even *Escherichia coli* [62,63], suggesting that transcriptional regulation may obey a universal principle across the genome, i.e., high-level expression is associated with the bursting with large size.

This global constraint results from the correlations among bursting parameters. Given that $\;\langle m\rangle =\frac{\tau_{m}}{\tau_{P}+\frac{1-p_{1}}{p_{1}}\tau_{S}}$, *τ*_p_ is expressed as a function of *p*_1_, *τ*_S_, and <*m*>, and, thus, *F* = 1 + $\frac{\left(1-p_{1}\right)\langle m\rangle \left(\langle m\rangle \tau_{S}-p_{1}\tau_{m}\right)\tau_{S}}{p_{1}^{2}\tau_{m}^{2}+\left(1-p_{1}\right)\langle m\rangle \tau_{S}^{2}}$. For fixed <*m*>, the minimum of *F*, *F*_min_, is determined by the ranges of *τ*_S_ and *p*_1_; if <*m*> > *τ*_m_/(2*τ*_S_), *F*_min_ equals 1 + $\frac{\left(1-p_{10}\right)\langle m\rangle \left(\langle m\rangle \tau_{S0}-p_{10}\tau_{m}\right)\tau_{S0}}{p_{10}^{2}\tau_{m}^{2}+\left(1-p_{10}\right)\langle m\rangle \tau_{S0}^{2}}$, where *τ*_S0_ is the minimum of *τ*_S_ and *p*_10_ is the maximum of *p*_1_. *F*_min_ is greater than one and rises with increasing <*m*> at high expression, as illustrated by the black curve in Fig. 4E, which is similar in trend to the black one in Fig. 4D (to justify that this global restriction is intrinsic to the transcriptional machinery, few constraints are imposed on parameter values; otherwise, if *τ*_I_ > *τ*_A_ were guaranteed, the 2 curves would match). A cluster of red curves is plotted in Fig. 4E, each beginning with the same parameters and ending with a point on the black line. Each curve represents the *F-*<*m*> curve for some gene, similar to that shown in Fig. 3B.

To recapitulate the lower bound on *F* at high expression levels in Fig. 4D, we systematically altered the values of *p*_1_, *τ*_S_, and *τ*_P_ and calculated the mean mRNA number and Fano factor in the case of *τ*_A_ < *τ*_I_. The scatter plot of *F* versus *m* has similarities to that in Fig. 4D (Fig. 4F). The global constraint becomes more striking when there exists a longer refractory period in the inactive phase or a higher transcription rate, such as in the case of large *τ*_S_ and small *τ*_P_, leading to sharp bursting peaks and enhancing the discernibility of burst profiles. By contrast, high-level expression is not necessarily associated with large Fano factor in the telegraph model and other phenomenological models (inset of Fig. 4F and Fig. S9), which arises from the independence of bursting parameters. Collectively, the global constraint reflects an inherent feature of transcriptional bursting.

### Transient dynamics reflect the underlying regulatory mode

**Fig. 5.**
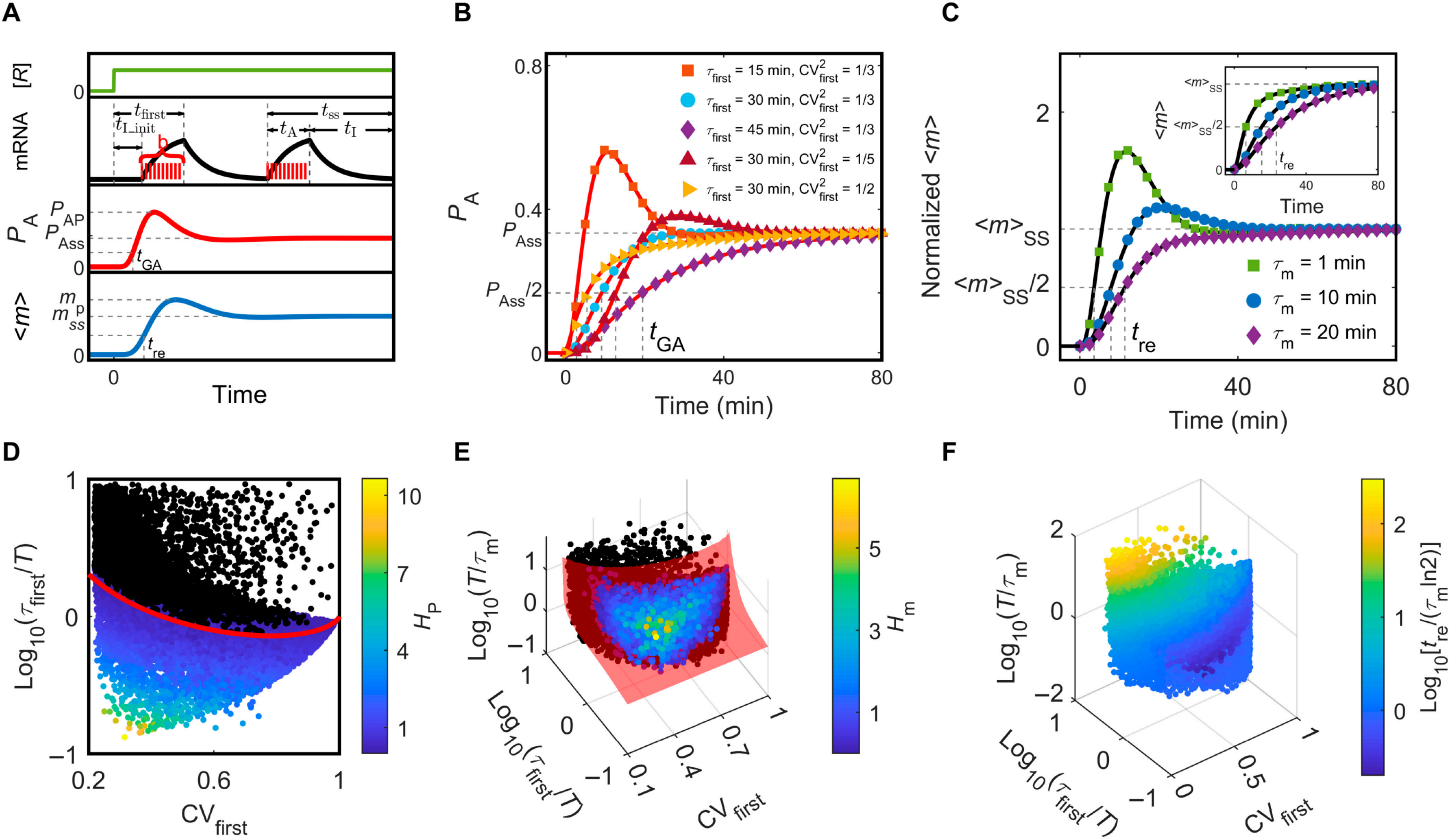
Response patterns upon stimulation. Time is in units of minutes. (A) Schematic of time courses of [*R*], the mRNA number (black; red bars represent Pol II entering productive elongation), the probability of gene activation, and the mean mRNA count (from top to bottom). The panel in the second row also marks the durations of the active phase (*t*_A_), inactive phase (*t*_I_), first inactive phase (*t*_I_init_), first burst (*t*_first_), and a burst in steady state (*t*_ss_). (B) Time courses of *P*_A_ with *τ*_A_ = 10, α_I_ = 2, and *θ*_I_ = 10. The *α* and *θ* of *f*_first_ are 3 and 5 (τ_first_ = 15, CV_first_^2^ = 1/3), 3 and 10 (τ_first_ = 30, CV_first_^2^ = 1/3), 3 and 15 (*τ*_first_ = 45, CV_first_^2^ = 1/3), 5 and 6 (τ_first_ = 30, CV_first_^2^ = 1/5), and 2 and 15 (τ_first_ = 30, CV_first_^2^ = 1/2), respectively. The data points are from simulation, while the solid curves are from analytical expression (Eq. H2 in Text S8). (C) Time courses of the mean mRNA number. *f*_A_ and *f*_I_ are the same as in (B); the *α* and *θ* of *f*_first_ are 3 and 5 (τ_first_ = 15, CV_first_^2^ = 1/3) for *τ*_m_ = 1, 10, or 20, and *P*_A_ overshoots. In the inset, the *α* and *θ* of *f*_first_ are 2 and 15 (τ_first_ = 30, CV_first_^2^ = 1/2) for *τ*_m_ = 1, 10, or 20, and *P*_A_ does not overshoot. <*m*>(*t*) is normalized by <*m*>_ss_. The data points are from simulation, while the solid curves are from Eq. H5. (D) Color-coded *H*_P_ controlled by *τ*_first_/*T* and CV_first_. The black points signify that *P*_A_ does not overshoot with *h*_P_*T* < 1, while the red curve corresponds to *h*_P_*T* = 1. Small values of *τ*_first_/*T* and CV_first_ lead to large *H*_P_. (E) Color-coded *H*_m_ controlled by *τ*_first_/*T*, CV_first_, and *T*/*τ*_m_. The black points denote that *m* does not overshoot with *h*_m_*T* < 1, while the red surface represents *h*_m_*T* = 1. (F) Color-coded *t*_re_ controlled by *τ*_first_/*T*, CV_first_, and *T*/*τ*_m_. In (D) to (F), the initial state is completely silent, and *τ*_A_ ∈ (0.1,10), *τ*_I_ ∈ (0.1,100), CV_P_ = 1, CV_i_ ∈ (0.1,1) (*i* = S1, S2, S3), and *τ*_I_init_ = *xτ*_I_ with *x* ∈ (0.1, 10).

After showing the typical features of transcriptional bursting in steady state, we turned to explore the transcriptional response to a step rise in regulator concentration. A new steady state will be reached in diverse manners because of different regulatory modes and composition of the inactive phase. With the initial state completely silent (i.e., *p*_1_ → 0, *p*_2_ → 1, or *τ*_i_ → ∞ and no mRNA production), the mean probability *P*_A_(*t*) of gene activation and mean mRNA number are determined as follows (see Text S8 for details):

$$\begin{array}{c} P_{A}\left(t\right)\approx f_{I_{\text{init}}}\left(t\right)*\delta \left(t\right)\sum_{i=0}^{\infty }{\left[*f_{A}\left(t\right)*f_{I}\left(t\right)\right]}^{i}*e^{-\frac{t}{\tau_{A}}}\\ <m>\left(t\right)\approx \frac{1}{\tau_{\text{P}}}e^{-\frac{t}{\tau_{\text{m}}}}*f_{I\_\text{init}}\left(t\right)*\delta \left(t\right)\sum_{i=0}^{\infty }{\left[*f_{A}\left(t\right)*f_{I}\left(t\right)\right]}^{i}*e^{-\frac{t}{\tau_{A}}} \end{array}$$ (6)

where *f*_A_(*t*_A_), *f*_I_(*t*_I_), and *f*_I_init_(*t*_I_init_) separately denote the distribution functions of the duration of the active (inactive) phase in the new steady state and the time taken to enter the active phase (Fig. 5A, top), with $f_{A}\left(t_{A}\right)=\frac{1}{\tau_{A}}e^{-\frac{t_{A}}{\tau_{A}}}$ and *f*_I_(*t*_I_) ≈ Γ(*t*_I_, *α*_I_, *θ*_I_) (Gamma distribution), while * represents convolution. Depending on the initial condition, *f*_I_init_ can assume different forms (Fig. S10). *f*_first_ = *f*_A_ * f_I_init_ represents the distribution function of the first burst duration *t*_first_, which is the time from stimulus onset to completion of mRNA synthesis in the first active phase, and can be expressed as Γ(*t*_first_, *α*_f_, *θ*_f_). *f*_T_ *= f*_A_ * f_I_ denotes the distribution function of burst duration in steady state (*t*_ss_). The mean and coefficient of variability (CV) of *t*_first_ are *τ*_first_ and CV_first_, respectively. The peak and steady-state values of *P*_A_ are separately *P*_AP_ and *P*_Ass_ = *τ*_A_/*T* (*T* is the mean burst period) (Fig. 5A, middle); <*m*>_p_ and <*m*>_SS_ = *bτ*_m_/*T* denote the mean peak and steady-state values of mRNA count, respectively (Fig. 5A, bottom). CV_Z_ (*Z* = P, S1, S2, S3, S) refers to the CV for *τ*_Z_ in steady state.

Figure 5B shows the typical dynamics of *P*_A_(*t*): *P*_A_ either overshoots *P*_Ass_ or monotonically rises toward *P*_Ass_. Without *P*_A_ overshooting, <*m*>_p_ cannot exceed <*m*>_ss_, and its dynamics also markedly depend on *τ*_m_ (Fig. 5C). To determine the condition for overshoot, we introduced *H*_P_ = (*P*_AP_ *− P*_Ass_)/*P*_Ass_ and *H*_m_ = (<*m*>_p_ − <*m*>_ss_)/<*m*>_ss_. *P*_A_ (<*m*>) must overshoot for *h*_P_*T* > 1 (*h*_m_*T* > 1), where $h_{x}=\frac{\alpha_{x}}{\delta_{x}+\tau_{\text{first}}}\frac{{\left(\alpha_{x}-1\right)}^{\alpha_{x}-1}}{\Gamma \left(\alpha_{x}\right)}e^{-\left(\alpha_{x}-1\right)}$ (*x* = P or m) with $\alpha_{P}=\frac{1}{{\mathrm{CV}}_{\text{first}}^{2}}$, $\alpha_{m}=\frac{{\left(\tau_{m}+\tau_{\text{first}}\right)}^{2}}{\tau_{m}^{2}+\tau_{\text{first}}^{2}{\mathrm{CV}}_{\text{first}}^{2}}$, *δ*_P_ = 0, and *δ*_m_ = *τ*_m_. *H*_P_ is governed by *τ*_first_/*T* and CV_first_; the area below the red curve of *h*_p_ = 1/*T* corresponds to *H*_p_ > 0 (Fig. 5D). *H*_m_ also relies on *τ*_m_/*T*, and the phase plane of *h*_m_ = 1/*T* separates *H*_m_ = 0 from *H*_m_ > 0 (Fig. 5E). Strikingly, overshoot appears at small values of *τ*_first_/*T*, CV_first_, and *τ*_m_/*T*.

On the other hand, the time taken to reach half of *P*_Ass_ (<*m*>_ss_) is denoted as *t*_GA_(*t*_re_); *t*_re_ is affected by mRNA production and degradation. If the rising phase of <*m*>(*t*) is governed by mRNA production over the time scale of *τ*_m_ due to strong correlation of bursts among individual simulations, *t*_re_ can be less than *τ*_m_ln2; otherwise, the dynamics of <*m*> are controlled by mRNA degradation, leading to *t*_re_ > *τ*_m_ln2. Thus, decreasing *τ*_first_/*T* and CV_first_ contributes to reducing *t*_re_; changing *T*/*τ*_m_ has a dual effect, and exclusively increasing it leads to a first drop and then rise in *t*_re_ (Fig. 5F).

In general, *τ*_first_/*T* and CV_first_ are modulated by the initial state distribution and constrained by the promoter architecture. When the gene state immediately enters the active phase upon stimulus onset due to regulation of Pol II-dependent reactions, *τ*_first_/*T* and CV_first_ are separately close to *τ*_A_/*T* and the CV of the active phase duration (*C*_A_, which is often ~1), whereas *τ*_first_/*T* and CV_first_ separately approach 1 and the CV of *t*_ss_ (i.e., CV_ss_) when the state just leaves the active phase. If there are multiple rate-limiting steps in burst cycle before stimulus onset [11,44], the initial state is mostly distributed around those states, usually leading to *τ*_A_/*T* < *τ*_first_/*T <* 1 and small CV_first_, whereas *τ*_first_/*T* ~ 1 and CV_first_ ~ 1 only if there is one rate-limiting step. The number of rate-limiting steps is governed by the promoter architecture [11,64,65] and is associated with the magnitude of *p*_2_. For large *p*_2_, the sum of all durations of *E*_S3_ within a burst cycle often accounts for the majority of the burst period, and its distribution is approximately exponential, implying that large *p*_2_ is often associated with only one rate-limiting step (i.e., nucleosome clearance) (see Text S2). By contrast, formation of the scaffold complex and pause of Pol II are the major rate-limiting steps for small *p*_2_. For instance, the nucleosome occupancy rate is rather low at target genes of white collar complex in *Neurospora crassa*, which is linked with small *p*_2_ and relatively large *τ*_S1_ + *τ*_S2_, leading to the observable overshoot [46]. Collectively, analyzing the transient response also provides insight into the modulatory mechanism and promoter architecture.

**Fig. 6.**
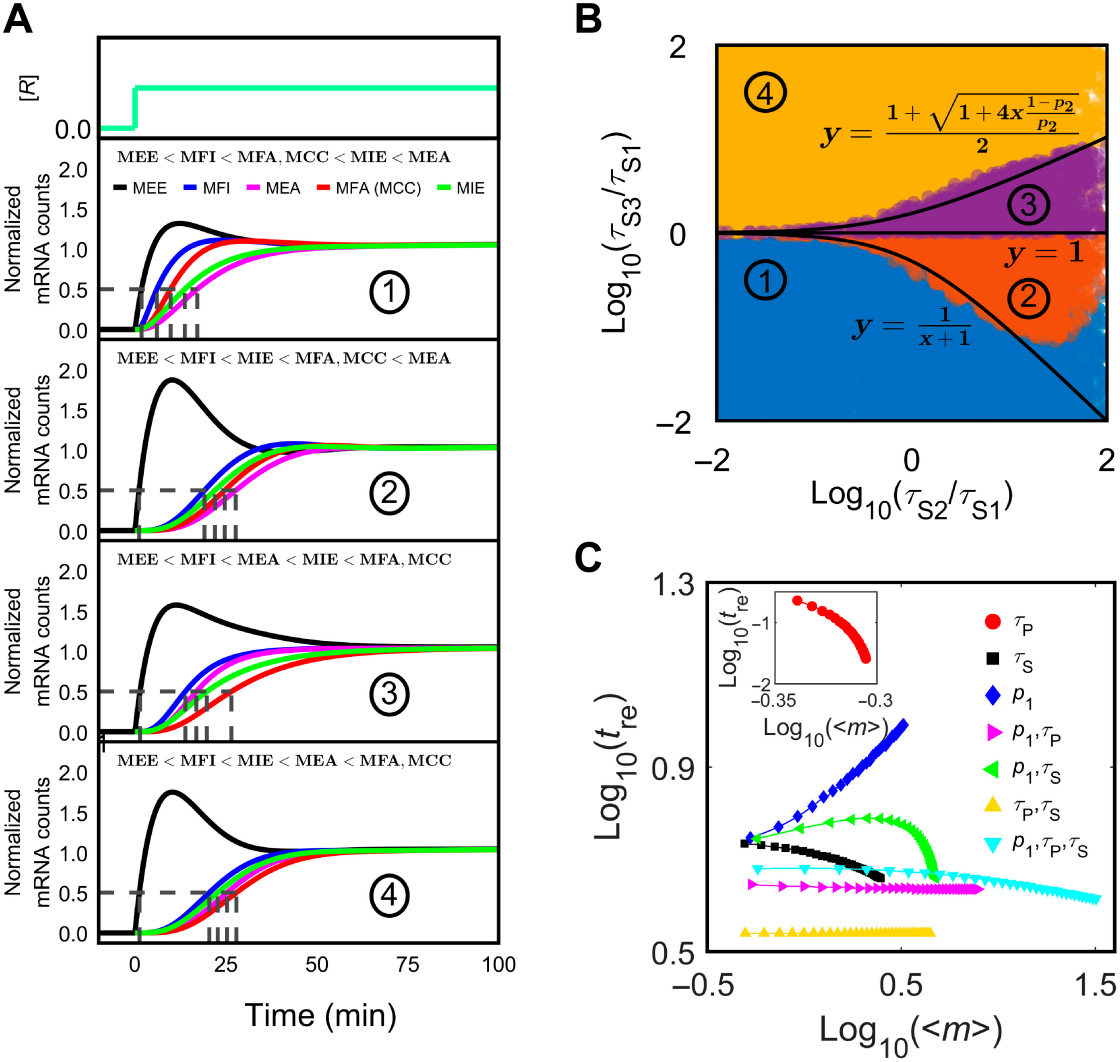
Transcriptional responses in different regulatory modes. Time is in units of minutes. (A) Transcriptional responses under different regulatory modes and initial conditions. There emerge 4 scenarios in terms of *t*_re_: MEE < MFI < MFA, MCC < MIE < MEA for *τ*_S1_ = 15, *τ*_S2_ = 2, and *τ*_S3_ = 4 after the stimulation; MEE < MFI < MIE < MFA, MCC < MEA for *τ*_S1_ = 10, *τ*_S2_ = 20, and *τ*_S3_ = 6; MEE < MFI < MEA < MIE < MFA, MCC for *τ*_S1_ = 3, *τ*_S2_ = 10, and *τ*_S3_ = 15; and MEE < MFI < MIE < MEA < MFA, MCC for *τ*_S1_ = 5, *τ*_S2_ = 20, and *τ*_S3_ = 8. The other parameters are fixed: *τ*_P_ = 1, *τ*_m_ = 10, *p*_1_ = 0.9, and *p*_2_ = 0.5. <*m*> is normalized by its steady-state value. (B) Two-parameter phase diagram of *x* = *τ*_S2_/*τ*_S1_ and *y* = *τ*_S3_/*τ*_S1_ at *p*_2_ = 0.5. The black curves constitute the boundaries. The blue region with *y <* 1/(*x*+1), red region with 1/(*x*+1) < *y* <1, purple region with $1<y<\left(1+\sqrt{1+4x\left(1-p_{2}\right)/p_{2}}\right)/2$, and yellow region with $y>\left(1+\sqrt{1+4x\left(1-p_{2}\right)/p_{2}}\right)/2\;$ correspond to the 4 scenarios in (A). (C) <*m*> and *t*_re_ for different regulatory modes and bursting parameters. The color represents the bursting parameters regulated. Default parameters are *τ*_PU_ = 1, *τ*_SU_ = 10, *p*_1U_ = 0.5, *τ*_m_ = 5, CV_P_^2^ = 1, and CV_S_^2^ = 1. Under a regulatory mode, the regulated parameters are decided by the regulator concentration, default parameters, and the corresponding parameters with bound regulators (*τ*_PB_ = 0.1, *τ*_SB_ = 1, and *p*_1B_ = 0.95).

Notably, only a finite number of scenarios emerge from the comparison of transient transcriptional responses across different regulatory modes. When the gene is activated from the silent state to the same steady state (with identical *f*_A_ and *f*_I_), for example, 4 scenarios appear upon examining the ordering of *t*_re_ values across 6 basic regulatory modes (Fig. 6A). These relationships primarily depend on the composition of the inactive phase, i.e., the values of *p*_2_, *τ*_S2_/*τ*_S1_, and *τ*_S3_/*τ*_S1_ (Fig. 6B). Nevertheless, the fastest response always occurs via MEE, as paused Pol II is poised to enter productive elongation upon an excitatory signal [66]; the second fastest response takes place via MFI, as the transcription machinery is ready for assembly on the promoter (Figs. S11 and S12). Under combinatory modes, a faster response can be induced to reach the same <*m*>_ss_ when *τ*_P_ is regulated (Fig. 6C). Therefore, accelerating the reactions involving Pol II can elicit fast transcription from a completely silent state.

Moreover, it may be beneficial for an inducible gene to respond fast to stimuli and generate a broad range of outputs. The rapid response largely results from the overshoot caused by regulating *τ*_P_ via MEE, while regulating *τ*_P_ in the case of *τ*_A_ > *τ*_I_ or regulating *p*_1_ and *τ*_S_ in the case of *τ*_A_ < *τ*_I_ elicits a broad range in <*m*>. Thus, integrating MEE with MX (X = IE, EA, FI, CC, or FA) allows for a fast response and highly tunable expression (Fig. 6C). In this sense, regulating *E_I_^P^* and *I_E_* is conducive to inducible genes [67].

## Discussion

The event model showcases several strengths when compared with previous phenomenological models. First, biochemical reactions involved in transcription are accurately mapped to the events in our model, and parameter values can be directly inferred from experimental data. Second, the event model retains the correlations among the bursting variables [transcription rate constant, burst size, and duration of the (in)active phase]. This naturally imposes constraints on burst profiles, guaranteeing bursty transcription in highly expressed genes [52,68,69] and enabling the modulation of burst frequency and burst size to separately predominate at low and high expression levels [47,57,70]. Third, 9 kinds of functional state transitions are distinctly separated in time scales from seconds to hours, and the durations and occurrence probabilities of events are independently modulated. These enable transcriptional bursting to manifest across a broad spectrum of temporal scales, exhibiting diverse dynamic patterns. With these features, the event model could have wide applications.

In principle, we can deduce the changes in (*τ*_P_, *τ*_S_, *p*_1_) through the correlations among observables and further reveal the promoter architecture and molecular regulatory mechanisms. For example, *E*_P_ consists of sequential stages (initiation, promoter clearance, elongation, and promoter-proximal pausing). The tendency of Pol II to continually synthesize mRNAs and the duration of promoter-proximal pause determine *p*_1_ and *τ*_P_, respectively. For a group of gap genes in early *Drosophila* embryos [47], *τ*_P_ is nearly fixed, but *p*_1_ is regulated, suggesting that regulators strongly affect the stability of the transcription complex and weakly affect the motion of Pol II along DNA templates, e.g., modulating the phosphorylation of Pol II C-terminal domain. In the regulation of *ctgf* expression by TGF-β1 [50], the duration of the active phase varies only slightly, while the transcription rate constant rises with increasing the TGF-β1 concentration, suggesting that TGF-β1 affects the ratio of forward to backward rates of Pol II movement along DNA templates during the stalling and reverting of the EEC. In the regulation of *sygl-*1 expression in *C. elegans*, the burst size remains relatively constant, while *τ*_A_ experiences changes [51], suggesting that GLP-1/Notch influences the duration of a highly irreversible stage, e.g., early elongation of Pol II until promoter-proximal pausing. Together, although the major stages in the event model are similar across genes, the reversibility and rate limits of those stages govern the regulatory modes, reflected in the changes to the durations and ordering of the events.

Cells are exposed to various fluctuations, and some have to rapidly respond and adapt to new environments via transcription [67]. For inducible genes with rapid transcriptional activation [66], it is desirable to trigger highly tunable expression and allow transcription overshoot to lift the limitation of mRNA lifetime on response speed. This can be achieved when *τ*_P_ and/or *p*_1_ (with *τ*_A_ > *τ*_I_) or *τ*_S_ and/or *p*_1_ (with *τ*_A_ < *τ*_I_) are regulated. Meanwhile, transcriptional overshoot can be evoked when Pol II is poised for transcription, or there exist multiple rate-limiting steps, e.g., a refractory period and PIC recruitment during the inactive phase. The genes are indeed prone to overshoot and respond fast in the presence of a refractory period [46], helpful for the rapid and accurate transmission of information. On the contrary, eukaryotic genes with TATA box often burst with a large size and seldom overshoot [11], conducive to filtering environmental fluctuations, and the TATA box seems to be correlated with lack of a refractory period.

The event model engages various types of regulatory factors; nevertheless, only the concentration of one regulator was altered, while the concentrations of others were kept constant in simulations. When transcriptional modulation involves coordinating multiple regulators and the relationships between their concentrations are known, it is possible to elucidate the combinatorial control of bursting parameters. We could determine the dependence of bursting parameters on the concentration of one of the involved regulators and generate <*m*>-*F* curves under diverse conditions. Note that the conclusions drawn in Figs. 3A, 4D to F, and 5 rely solely on the values of *τ*_P_, *τ*_S1_, *τ*_S2_, *τ*_S3_, *p*_1_, and *p*_2_ and are not influenced by the single-regulator assumption. In Figs. 3C and D and 4A to C, the experimental data were collected when only one kind of regulator was monitored. If the concentrations of multiple regulators change simultaneously, the trends depicted in Fig. 3B may undergo changes, although the <*m*>-*F*_τP_ and <*m*>-*F*_τS_ curves still constitute the boundaries.

Transcriptional noise can be divided into intrinsic and extrinsic noise [71]. Here, the intrinsic noise is mainly manifested in the stochasticity in event orders, event durations, and mRNA degradation. Treating mRNA degradation as a one-step process is a simplification; however, it was reported that, in a broad range of parameter space, models assuming either multistep or one-step degradation of mRNA yield indistinguishable mRNA count distributions [16]. The current work did not explore extrinsic noise, which is influenced by various factors including cell volume, gene copies, DNA replication, and cell cycle progression [72,73]. For example, it has been shown that the presence of 2 uncorrelated alleles halves the noise intensity [56]. It was reported that extrinsic noise typically leads to a more dispersed mRNA count distribution, such as an increased probability of low numbers or a longer tail, and that extrinsic noise contributes substantially to the total noise at high expression levels, leading to a higher noise plateau [11]. It was also demonstrated that parameter variability among cells should be taken into account to fully interpret the experimental data [54]. Furthermore, when analyzing data obtained through single-cell measurements, technological noise is also unavoidable [53]. All these suggest that the event model has to be extended to incorporate more sources of noise, and it would be interesting to dissect the contributions of intrinsic and extrinsic noise.

The current work explored the generic case where the dwell times of regulators are much shorter than the time taken to switch between the inactive and active phases, regulator binding promotes transcription with monotonic gene-regulatory functions, and only one gene locus is involved [5,34,35,74]. More advanced models could be developed to probe the gene-specific transcriptional activity. If the concentration of regulators varies periodically (e.g., the pulsing of p53 and nuclear factor κB levels), how transcriptional bursting is modulated to reliably code signals could be the focus of further study.

In summary, the event model, built upon a small set of adjustable functional events, offers a clear and straightforward description of transcriptional progression. This approach facilitates the investigation of both shared and specific mechanisms for gene regulation. Modulation can occur at any stage of a burst cycle, but the underlying regulatory modes are limited in number because of intrinsic correlations among transcriptional events. Our results suggest that large Fano factor is required for transcriptional bursting at high expression levels and the transitions *E_I_^P^ and I_E_*, involved in the recruitment, pause and release, or elongation of Pol II, are pivotal regulatory targets for enhancing the transcriptional sensitivity, anti-crosstalk, adaptability, and responsiveness. Combining experimental data with the event model allows for deducing the molecular processes that predominantly govern transcriptional activity.

## Methods

The development of the event model is an important part of this work, and the model is the basis of the whole study. Thus, we provided the details on building the model in the “Establishment of the event model” section. Texts S1 to S8 further present the derivation of formula and explanations. We made a detailed reference to the Supplementary Materials in the text.

## Data Availability

All data needed to evaluate the conclusions in the paper are present in the paper. Custom codes will be available upon request to F.L.

## Supplementary Materials

Supplementary 1 Text S1. Establishment of the event model. Text S2. Structure of the event model. Text S3. Simulation of the event model and phenomenological models. Text S4. Regulation of transcriptional bursting. Text S5. Comparison between the single-binding and multibinding sites cases. Text S6. Gene regulatory function. Text S7. Mean and variance of mRNA numbers. Text S8. Transient response to stimulation. Fig. S1. Events in transcriptional bursting. Fig. S2. Duration distribution of the process that consists of 2 sequential processes. Fig. S3. Duration distribution of a mixed process. Fig. S4. Probability of repeating events *E*_P_ and *E*_S3_. Fig. S5. Relationships between the mean (<*m>*) and variance (σ_m_^2^) of mRNA numbers for different distributions of *f*_P_ and *f*_S_. Fig. S6. Relationships between the mean (*<*m*>*) and relative noise strength (𝜂_m_^2^ = 𝜎_m_^2^/<*m>*^2^) of mRNA numbers for different *f*_P_ and *f*_S_. Fig. S7. Relationships between the mean (<*m>*) and variance (σ_m_^2^) of mRNA numbers under different regulatory modes. Fig. S8. Transcriptional kinetics under different combination modes. Fig. S9. Difference between the telegraph and event models under different regulatory modes. Fig. S10. Response curves for different distribution functions of the time taken to activate the gene from the initial state. Fig. S11. Regulatory range (*υ*_max_) and response speed for basic regulatory modes. Fig. S12. Transcriptional sensitivity under different regulatory modes.

## References

[1] Neuert G, Munsky B, Tan RZ, Teytelman L, Khammash M, van Oudenaarden A. Systematic identification of signal-activated stochastic gene regulation. Science. 2013;339(6119):584–587. 23372015 10.1126/science.1231456 PMC3751578

[2] Ezer D, Moignard V, Göttgens B, Adryan B. Determining physical mechanisms of gene expression regulation from single cell gene expression data. PLOS Comput Biol. 2016;12(8): e1005072. 27551778 10.1371/journal.pcbi.1005072 PMC4995004

[3] Lis JT. A 50 year history of technologies that drove discovery in eukaryotic transcription regulation. Nat Struct Mol Biol. 2019;26(9):777–782. 31439942 10.1038/s41594-019-0288-9 PMC7106917

[4] Coulon A, Chow CC, Singer RH, Larson DR. Eukaryotic transcriptional dynamics: From single molecules to cell populations. Nat Rev Genet. 2013;14(8):572–584. 23835438 10.1038/nrg3484 PMC3807637

[5] Lu F, Lionnet T. Transcription factor dynamics. Cold Spring Harb Perspect Biol. 2021;13(11): a040949. 34001530 10.1101/cshperspect.a040949 PMC8559544

[6] Wissink EM, Vihervaara A, Tippens ND, Lis JT. Nascent RNA analyses: Tracking transcription and its regulation. Nat Rev Genet. 2019;20(12):705–723. 31399713 10.1038/s41576-019-0159-6 PMC6858503

[7] Chen X, Zhang D, Su N, Bao B, Xie X, Zuo F, Yang L, Wang H, Jiang L, Lin Q, et al. Visualizing RNA dynamics in live cells with bright and stable fluorescent RNAs. Nat Biotechnol. 2019;37(11):1287–1293. 31548726 10.1038/s41587-019-0249-1

[8] Peccoud J, Ycart B. Markovian modeling of gene-product synthesis. Theor Popul Biol. 1995;48(2):222–234.

[9] Pedraza JM, Paulsson J. Effects of molecular memory and bursting on fluctuations in gene expression. Science. 2008;319(5861):339–343. 18202292 10.1126/science.1144331

[10] Zhang J, Chen L, Zhou T. Analytical distribution and tunability of noise in a model of promoter progress. Biophys J. 2012;102(6):1247–1257. 22455907 10.1016/j.bpj.2012.02.001 PMC3309289

[11] Zoller B, Nicolas D, Molina N, Naef F. Structure of silent transcription intervals and noise characteristics of mammalian genes. Mol Syst Biol. 2015;11(7):823. 26215071 10.15252/msb.20156257 PMC4547851

[12] Corrigan AM, Tunnacliffe E, Cannon D, Chubb JR. A continuum model of transcriptional bursting. eLife. 2016;5: e13501. 10.7554/eLife.13051 PMC4850746 26896676

[13] Tantale K, Mueller F, Kozulic-Pirher A, Lesne A, Victor J-M, Robert M-C, Capozi S, Chouaib R, Bäcker V, Mateos-Langerak J, et al. A single-molecule view of transcription reveals convoys of RNA polymerases and multi-scale bursting. Nat Commun. 2016;7:12248. 27461529 10.1038/ncomms12248 PMC4974459

[14] Zhang J, Zhou T. Promoter-mediated transcriptional dynamics. Biophys J. 2014;106(2):479–488. 24461023 10.1016/j.bpj.2013.12.011 PMC3907263

[15] Szavits-Nossan J, Grima R. Steady-state distributions of nascent RNA for general initiation mechanisms. Phys Rev Res. 2023;5(1): 013064.

[16] Braichenko S, Holehouse J, Grima R. Distinguishing between models of mammalian gene expression: Telegraph-like models versus mechanistic models. J R Soc Interface. 2021;18(183):20210510. 34610262 10.1098/rsif.2021.0510 PMC8492181

[17] Karmakar R. Control of noise in gene expression by transcriptional reinitiation. J Stat Mech. 2020;2020: 063402.

[18] Karmakar R, Das AK. Effect of transcription reinitiation in stochastic gene expression. J Stat Mech. 2021;2021: 033502.

[19] Cao Z, Filatova T, Oyarzún DA, Grima R. A stochastic model of gene expression with polymerase recruitment and pause release. Biophys J. 2020;119(5):1002–1014. 32814062 10.1016/j.bpj.2020.07.020 PMC7474183

[20] Muthukrishnan AB, Kandhavelu M, Lloyd-Price J, Kudasov F, Chowdhury S, Yli-Harja O, Ribeiro AS. Dynamics of transcription driven by the *tetA* promoter, one event at a time, in live *Escherichia coli* cells. Nucleic Acids Res. 2012;40(17):8472–8483. 22730294 10.1093/nar/gks583 PMC3458540

[21] Lloyd-Price J, Startceva S, Kandavalli V, Chandraseelan JG, Goncalves N, Oliveira SMD, Häkkinen A, Ribeiro AS. Dissecting the stochastic transcription initiation process in live *Escherichia coli*. DNA Res. 2016;23(3):203–214. 27026687 10.1093/dnares/dsw009 PMC4909308

[22] Weidemann DE, Holehouse J, Singh A, Grima R, Hauf S. The minimal intrinsic stochasticity of constitutively expressed eukaryotic genes is sub-Poissonian. Sci Adv. 2023;9(32):eadh5138. 37556551 10.1126/sciadv.adh5138 PMC10411910

[23] Hansen AS, Zechner C. Promoters adopt distinct dynamic manifestations depending on transcription factor context. Mol Syst Biol. 2021;17(2): e9821. 33595925 10.15252/msb.20209821 PMC7888307

[24] Scholes C, DePace AH, Sánchez Á. Combinatorial gene regulation through kinetic control of the transcription cycle. Cell Syst. 2017;4(1):97–108.e9. 28041762 10.1016/j.cels.2016.11.012 PMC5469051

[25] Wang Y, Liu F, Wang W. Dynamic mechanism for the transcription apparatus orchestrating reliable responses to activators. Sci Rep. 2012;2:422. 22639730 10.1038/srep00422 PMC3360325

[26] Wang Y, Ni T, Wang W, Liu F. Gene transcription in bursting: A unified model for realizing accuracy and stochasticity. Biol Rev. 2019;94(1):248–258. 30024089 10.1111/brv.12452 PMC7379551

[27] Wang Y, Liu F, Wang W. Kinetics of transcription initiation directed by multiple *cis*-regulatory elements on the *glnAp2* promoter. Nucleic Acids Res. 2016;44(22):10530–10538. 27899598 10.1093/nar/gkw1150 PMC5159524

[28] Jia T, Kulkarni RV. Intrinsic noise in stochastic models of gene expression with molecular memory and bursting. Phys Rev Lett. 2011;106(5): 058102. 21405439 10.1103/PhysRevLett.106.058102

[29] Zhang Z, Deng Q, Wang Z, Chen Y, Zhou T. Exact results for queuing models of stochastic transcription with memory and crosstalk. Phys Rev E. 2021;103(6): 062414. 34271765 10.1103/PhysRevE.103.062414

[30] Schwabe A, Rybakova KN, Bruggeman FJ. Transcription stochasticity of complex gene regulation models. Biophys J. 2012;103(6):1152–1161. 22995487 10.1016/j.bpj.2012.07.011 PMC3446772

[31] Nicolas D, Zoller B, Suter DM, Naef F. Modulation of transcriptional burst frequency by histone acetylation. Proc Natl Acad Sci USA. 2018;115(27):7153–7158. 29915087 10.1073/pnas.1722330115 PMC6142243

[32] Donovan BT, Huynh A, Ball DA, Patel HP, Poirier MG, Larson DR, Ferguson ML, Lenstra TL. Live-cell imaging reveals the interplay between transcription factors, nucleosomes, and bursting. EMBO J. 2019;38(12): e100809. 31101674 10.15252/embj.2018100809 PMC6576174

[33] Zabidi MA, Stark A. Regulatory enhancer-core-promoter communication via transcription factors and cofactors. Trends Genet. 2016;32(12):801–814. 27816209 10.1016/j.tig.2016.10.003 PMC6795546

[34] Voss TC, Hager GL. Dynamic regulation of transcriptional states by chromatin and transcription factors. Nat Rev Genet. 2014;15(2):69–81. 24342920 10.1038/nrg3623 PMC6322398

[35] Suter DM. Transcription factors and DNA play hide and seek. Trends Cell Biol. 2020;30(6):491–500. 32413318 10.1016/j.tcb.2020.03.003

[36] Brown CR, Mao CH, Falkovskaia E, Jurica MS, Boeger H. Linking stochastic fluctuations in chromatin structure and gene expression. PLOS Biol. 2013;11(8): e1001621. 23940458 10.1371/journal.pbio.1001621 PMC3735467

[37] Muramoto T, Müller I, Thomas G, Melvin A, Chubb JR. Methylation of H3K4 is required for inheritance of active transcriptional states. Curr Biol. 2010;20(5):397–406. 20188556 10.1016/j.cub.2010.01.017

[38] Casamassimi A, Napoli C. Mediator complexes and eukaryotic transcription regulation: An overview. Biochimie. 2007;89(12):1439–1446. 17870225 10.1016/j.biochi.2007.08.002

[39] Malik S, Roeder RG. Dynamic regulation of pol II transcription by the mammalian mediator complex. Trends Biochem Sci. 2005;30(5):256–263. 15896744 10.1016/j.tibs.2005.03.009

[40] Jonkers I, Kwak H, Lis JT. Genome-wide dynamics of pol II elongation and its interplay with promoter proximal pausing, chromatin, and exons. eLife. 2014;3: e02407. 24843027 10.7554/eLife.02407 PMC4001325

[41] Tome JM, Tippens ND, Lis JT. Single-molecule nascent RNA sequencing identifies regulatory domain architecture at promoters and enhancers. Nat Genet. 2018;50(11):1533–1541. 30349116 10.1038/s41588-018-0234-5 PMC6422046

[42] Shao W, Zeitlinger J. Paused RNA polymerase II inhibits new transcriptional initiation. Nat Genet. 2017;49(7):1045–1051. 28504701 10.1038/ng.3867

[43] Kumar N, Singh A, Kulkarni RV. Transcriptional bursting in gene expression: Analytical results for general stochastic models. PLOS Comput Biol. 2015;11(10): e1004292. 26474290 10.1371/journal.pcbi.1004292 PMC4608583

[44] Suter DM, Molina N, Gatfield D, Schneider K, Schibler U, Naef F. Mammalian genes are transcribed with widely different bursting kinetics. Science. 2011;332(6028):472–474. 21415320 10.1126/science.1198817

[45] Hendy O, Campbell J Jr, Weissman JD, Larson DR, Singer DS. Differential context-specific impact of individual core promoter elements on transcriptional dynamics. Mol Biol Cell. 2017;28(23):3360–3370. 28931597 10.1091/mbc.E17-06-0408 PMC5687036

[46] Li C, Cesbron F, Oehler M, Brunner M, Hӧfer T. Frequency modulation of transcriptional bursting enables sensitive and rapid gene regulation. Cell Syst. 2018;6(4):409–423.e11. 29454937 10.1016/j.cels.2018.01.012

[47] Zoller B, Little SC, Gregor T. Diverse spatial expression patterns emerge from unified kinetics of transcriptional bursting. Cell. 2018;175(3):835–847. 30340044 10.1016/j.cell.2018.09.056 PMC6779125

[48] Garcia HG, Tikhonov M, Lin A, Gregor T. Quantitative imaging of transcription in living *Drosophila* embryos links polymerase activity to patterning. Curr Biol. 2013;23(21):2140–2145. 24139738 10.1016/j.cub.2013.08.054 PMC3828032

[49] Hafner A, Reyes J, Stewart-Ornstein J, Tsabar M, Jambhekar A, Lahav G. Quantifying the central dogma in the p53 pathway in live single cells. Cell Syst. 2020;10(6):495–505. 32533938 10.1016/j.cels.2020.05.001 PMC7413213

[50] Molina N, Suter DM, Cannavo R, Zoller B, Gotic I, Naef F. Stimulus-induced modulation of transcriptional bursting in a single mammalian gene. Proc Natl Acad Sci USA. 2013;110(51):20563–20568. 24297917 10.1073/pnas.1312310110 PMC3870742

[51] Lee CH, Shin H, Kimble J. Dynamics of Notch-dependent transcriptional bursting in its native context. Dev Cell. 2019;50(4):426–435.e4. 31378588 10.1016/j.devcel.2019.07.001 PMC6724715

[52] Larsson AJM, Johnsson P, Hagemann-Jensen M, Hartmanis L, Faridani OR, Reinius B, Segerstolpe Å, Rivera CM, Ren B, Sandberg R. Genomic encoding of transcriptional burst kinetics. Nature. 2019;565(7738):251–254. 30602787 10.1038/s41586-018-0836-1 PMC7610481

[53] Grün D, Kester L, van Oudenaarden A. Validation of noise models for single-cell transcriptomics. Nat Methods. 2014;11(6):637–640. 24747814 10.1038/nmeth.2930

[54] Grima R, Esmenjaud P-M. Systematic biases in transcriptional parameters inferred from single-cell snapshot data. bioRxiv. 2023;2023.06.19.545536.

[55] Yunger S, Rosenfeld L, Garini Y, Shav-Tal Y. Single-allele analysis of transcription kinetics in living mammalian cells. Nat Methods. 2010;7(8):631–633. 20639867 10.1038/nmeth.1482

[56] Raj A, Peskin CS, Tranchina D, Vargas DY, Tyagi S. Stochastic mRNA synthesis in mammalian cells. PLOS Biol. 2006;4(10): e309. 17048983 10.1371/journal.pbio.0040309 PMC1563489

[57] Dar RD, Razooky BS, Singh A, Trimeloni TV, McCollum JM, Cox CD, Simpson ML, Weinberger LS. Transcriptional burst frequency and burst size are equally modulated across the human genome. Proc Natl Acad Sci USA. 2012;109(43):17454–17459. 23064634 10.1073/pnas.1213530109 PMC3491463

[58] Itzkovitz S, Lyubimova A, Blat IC, Maynard M, van Es J, Lees J, Jacks T, Clevers H, van Oudenaarden A. Single-molecule transcript counting of stem-cell markers in the mouse intestine. Nat Cell Biol. 2012;14(1):106–114. 10.1038/ncb2384 PMC3292866 22119784

[59] Little SC, Tikhonov M, Gregor T. Precise developmental gene expression arises from globally stochastic transcriptional activity. Cell. 2013;154(4):789–800. 23953111 10.1016/j.cell.2013.07.025 PMC3778922

[60] Pare A, Lemons D, Kosman D, Beaver W, Freund Y, McGinnis W. Visualization of individual *Scr* mRNAs during *Drosophila* embryogenesis yields evidence for transcriptional bursting. Curr Biol. 2009;19(23):2037–2042. 19931455 10.1016/j.cub.2009.10.028 PMC2805773

[61] Boettiger AN, Levine M. Rapid transcription fosters coordinate snail expression in the *Drosophila* embryo. Cell Rep. 2013;3(1):8–15. 23352665 10.1016/j.celrep.2012.12.015 PMC4257496

[62] Engl C, Jovanovic G, Brackston RD, Kotta-Loizou I, Buck M. The route to transcription initiation determines the mode of transcriptional bursting in *E. coli*. Nat Commun. 2020;11(1):2422. 32415118 10.1038/s41467-020-16367-6 PMC7229158

[63] Zaslaver A, Bren A, Ronen M, Itzkovitz S, Kikoin I, Shavit S, Liebermeister W, Surette MG, Alon U. A comprehensive library of fluorescent transcriptional reporters for *Escherichia coli*. Nat Methods. 2006;3(8):623–628. 16862137 10.1038/nmeth895

[64] Valen E, Sandelin A. Genomic and chromatin signals underlying transcription start-site selection. Trends Genet. 2011;27(11):475–485. 21924514 10.1016/j.tig.2011.08.001

[65] Lenhard B, Sandelin A, Carninci P. Metazoan promoters: Emerging characteristics and insights into transcriptional regulation. Nat Rev Genet. 2012;13(4):233–245. 22392219 10.1038/nrg3163

[66] Muse GW, Gilchrist DA, Nechaev S, Shah R, Parker JS, Grissom SF, Zeitlinger J, Adelman K. RNA polymerase is poised for activation across the genome. Nat Genet. 2007;39(12):1507–1511. 17994021 10.1038/ng.2007.21 PMC2365887

[67] Weake VM, Workman JL. Inducible gene expression: Diverse regulatory mechanisms. Nat Rev Genet. 2010;11(6):426–437. 20421872 10.1038/nrg2781

[68] Sanchez A, Golding I. Genetic determinants and cellular constraints in noisy gene expression. Science. 2013;342(6163):1188–1193. 24311680 10.1126/science.1242975 PMC4045091

[69] Battich N, Stoeger T, Pelkmans L. Control of transcript variability in single mammalian cells. Cell. 2015;163(7):1596–1610. 26687353 10.1016/j.cell.2015.11.018

[70] Lammers NC, Galstyan V, Reimer A, Medin SA, Wiggins CH, Garcia HG. Multimodal transcriptional control of pattern formation in embryonic development. Proc Natl Acad Sci USA. 2020;117(2):836–847. 31882445 10.1073/pnas.1912500117 PMC6969519

[71] Swain PS, Elowitz MB, Siggia ED. Intrinsic and extrinsic contributions to stochasticity in gene expression. Proc Natl Acad Sci USA. 2002;99(20):12795–12800. 12237400 10.1073/pnas.162041399 PMC130539

[72] Cao Z, Grima R. Analytical distributions for detailed models of stochastic gene expression in eukaryotic cells. Proc Natl Acad Sci USA. 2020;117(9):4682–4692. 32071224 10.1073/pnas.1910888117 PMC7060679

[73] Fu X, Patel HP, Coppola S, Xu L, Cao Z, Lenstra TL, Grima R. Quantifying how post-transcriptional noise and gene copy number variation bias transcriptional parameter inference from mRNA distributions. eLife. 2022;11: e82493. 36250630 10.7554/eLife.82493 PMC9648968

[74] Zhang Z, English BP, Grimm JB, Kazane SA, Hu W, Tsai A, Inouye C, You C, Piehler J, Schultz PG, et al. Rapid dynamics of general transcription factor TFIIB binding during preinitiation complex assembly revealed by single-molecule analysis. Genes Dev. 2016;30(18):2106–2118. 27798851 10.1101/gad.285395.116 PMC5066616

[75] Roeder RG. 50+ years of eukaryotic transcription: An expanding universe of factors and mechanisms. Nat Struct Mol Biol. 2019;26(9):783–791. 31439941 10.1038/s41594-019-0287-x PMC6867066

[76] Yakovchuk P, Gilman B, Goodrich JA, Kugel JF. RNA polymerase II and TAFs undergo a slow isomerization after the polymerase is recruited to promoter-bound TFIID. J Mol Biol. 2010;397(1):57–68. 20083121 10.1016/j.jmb.2010.01.025

[77] Warfield L, Ramachandran S, Baptista T, Devys D, Tora L, Hahn S. Transcription of nearly all yeast RNA polymerase II-transcribed genes is dependent on transcription factor TFIID. Mol Cell. 2017;68(1):118–129.e5. 28918900 10.1016/j.molcel.2017.08.014 PMC5679267

[78] Blair RH, Goodrich JA, Kugel JF. Single-molecule fluorescence resonance energy transfer shows uniformity in TATA binding protein-induced DNA bending and heterogeneity in bending kinetics. Biochemistry. 2012;51(38):7444–7455. 22934924 10.1021/bi300491j PMC3551999

[79] Bartman CR, Hsu SC, Hsiung CCS, Raj A, Blobel GA. Enhancer regulation of transcriptional bursting parameters revealed by forced chromatin looping. Mol Cell. 2016;62(2):237–247. 27067601 10.1016/j.molcel.2016.03.007 PMC4842148

[80] Chen H, Levo M, Barinov L, Fujioka M, Jaynes JB, Gregor T. Dynamic interplay between enhancer-promoter topology and gene activity. Nat Genet. 2018;50(9):1296–1303. 30038397 10.1038/s41588-018-0175-z PMC6119122

[81] Saunders A, Core LJ, Lis JT. Breaking barriers to transcription elongation. Nat Rev Mol Cell Biol. 2006;7(8):557–567. 16936696 10.1038/nrm1981

