# Supplementary material for: Regulatory Mechanisms for Transcriptional Bursting Revealed by an Event-Based Model
Source: Research (Wash D C). 2023 Oct 24;6:0253. doi: 10.34133/research.0253 (PMC11407585; doi:10.34133/research.0253)
Supplement: Supplementary 1 — Text S1. Establishment of the event model. Text S2. Structure of the event model. Text S3. Simulation of the event model and phenomenological models. Text S4. Regulation of transcriptional bursting. Text S5. Comparison between the single-binding and multibinding sites cases. Text S6. Gene regulatory function. Text S7. Mean and variance of mRNA numbers. Text S8. Transient response to stimulation. Fig. S1. Events in transcriptional bursting. Fig. S2. Duration distribution of the process that consists of 2 sequential processes. Fig. S3. Duration distribution of a mixed process. Fig. S4. Probability of repeating events EP and ES3. Fig. S5. Relationships between the mean () and variance (σm2) of mRNA numbers for different distributions of fP and fS. Fig. S6. Relationships between the mean () and relative noise strength (𝜂m2 = 𝜎m2/2) of mRNA numbers for different fP and fS. Fig. S7. Relationships between the mean () and variance (σm2) of mRNA numbers under different regulatory modes. Fig. S8. Transcriptional kinetics under different combination modes. Fig. S9. Difference between the telegraph and event models under different regulatory modes. Fig. S10. Response curves for different distribution functions of the time taken to activate the gene from the initial state. Fig. S11. Regulatory range (υmax) and response speed for basic regulatory modes. Fig. S12. Transcriptional sensitivity under different regulatory modes. [file research.0253.f1.docx]

**Supplementary Material**

**Regulatory mechanisms for transcriptional bursting revealed by an event-based model**

# This document comprises Supplemental Texts (Sections 1-8) and 12 Supplemental figures.

**Section 1: Establishment of the event model**

Assume that there are $n_{X}$ states in set X (X = E, I, A, F, C). *P*_i_ (*m*; *t*) ($i=1, 2,\cdots,l$;$l=\sum_{X} n_{X}$) is the probability of state $i$ with *m* mRNA transcripts at time *t*. $\mathbf{P}_{X}$ = ($P_{X,1}$, $P_{X,2}$,$\cdots$, $P_{X,n_{X}}$)^T^ and **P** = (**P**_E_^T^, **P**_I_^T^, **P**_A_^T^, **P**_F_^T^, **P**_C_^T^)^T^ (or **P =** (*P*_1_, *P*_2_, $\cdots$, *P*_l_)^T^). ***K***_XX'_ is an *n*_X_ × *n*_X'_ matrix, describing the state transitions between sets *X* and *X'*. ***K***_XX'_ = (*λ*_XX', ij_), where *λ*_XX', ij_ (*X* ≠ *X'* or *X* = *X'* with *i* ≠ *j*) is the transition rate from state *i* in *X* to state *j* in *X'* and $\lambda_{\mathrm{XX},ii}=-\sum\lambda_{XX^{'},ij}$is the opposite of the transition rate from state *i* in *X* to other states (*λ* = 0 if there is no transition). The biochemical master equation describing mRNA dynamics is as follows:

|  | $\frac{d\mathbf{P}\left( m;t \right)}{dt}=\mathbf{K}^{\mathbf{S}}\mathbf{P}\left( m;t \right)+\delta_{m}\left( \mathbf{E}-\mathbf{I} \right)\left[ m\mathbf{P}\left( m;t \right) \right]+\mathbf{K}^{P}\mathbf{P}\left( m-1;t \right)$, | (A1) |
| --- | --- | --- |

with

|  | $\mathbf{K}^{P}=\left( \begin{matrix} \mathbf{0} & \mathbf{0} & \mathbf{0} & \mathbf{0} & \mathbf{0} \\ \mathbf{K}_{\mathrm{EI}}^{P} & \mathbf{0} & \mathbf{0} & \mathbf{0} & \mathbf{0} \\ \mathbf{0} & \mathbf{0} & \mathbf{0} & \mathbf{0} & \mathbf{0} \\ \mathbf{0} & \mathbf{0} & \mathbf{0} & \mathbf{0} & \mathbf{0} \\ \mathbf{0} & \mathbf{0} & \mathbf{0} & \mathbf{0} & \mathbf{0} \end{matrix} \right)$, $\mathbf{K}^{\mathbf{S}}=\left( \begin{matrix} \mathbf{K}_{\mathrm{EE}} & \mathbf{K}_{\mathrm{IE}} & \mathbf{0} & \mathbf{0} & \mathbf{0} \\ \mathbf{K}_{\mathbf{EI}}^{S} & \mathbf{K}_{\mathrm{II}} & \mathbf{K}_{\mathbf{AI}} & \mathbf{0} & \mathbf{0} \\ \mathbf{0} & \mathbf{K}_{\mathrm{IA}} & \mathbf{K}_{\mathrm{AA}} & \mathbf{K}_{\mathrm{FA}} & \mathbf{0} \\ \mathbf{0} & \mathbf{0} & \mathbf{K}_{\mathrm{AF}} & \mathbf{K}_{\mathrm{FF}} & \mathbf{K}_{\mathrm{CF}} \\ \mathbf{0} & \mathbf{0} & \mathbf{0} & \mathbf{K}_{\mathrm{FC}} & \mathbf{K}_{\mathrm{CC}} \end{matrix} \right)$. |  |
| --- | --- | --- |

$\delta_{m}$ is the degradation rate constant. **E** is a shift operator, i.e., **E**[*f* (*m*)] = *f* (*m*+1), and **I** is the identity operator. $\mathbf{K}_{\mathrm{EI}}^{P}$ *+* $\mathbf{K}_{\mathrm{EI}}^{S}$ *=* $\mathbf{K}_{\mathrm{EI}}$, where $\mathbf{K}_{\mathrm{EI}}^{P}$ and $\mathbf{K}_{\mathrm{EI}}^{S}$ separately describe the transitions with and without mRNA production.

Assume ***Q***_X_(*t*) = (*Q*_X,1_(*t*), *Q*_X,2_(*t*), $\cdots$, $Q_{X,n_{X}}\left( t \right)$)^T^ and ***Q****_X_*(0) = ***Q***_init_, where ***Q***_init_ is the probability of the initial state and *Q*_X_*_,_*_i_(*t*) is the survival probability of *i-*th state in set *X* at time *t*. For the process *X_X’_* ($X,X^{'}\in$ {C, F, A, I, E}; the transitions are *C_F_*, *F_C_*, *F_A_*, *A_F_*, *A_I_*, *I_A_*, *I_E_*, *E_I_* and *E_I_^P^*), ***Q***_X_(*t*) is the probability of retaining the states in *X* after *t*, satisfying

|  | $\frac{d\boldsymbol{Q}_{X}\left( t \right)}{dt}=\boldsymbol{K}_{\mathrm{XX}}\boldsymbol{Q}_{X}\left( t \right)$, | (A2) |
| --- | --- | --- |

which leads to

|  | $\boldsymbol{Q}_{X}\left( t \right)=e^{\boldsymbol{K}_{\mathrm{XX}}t}\boldsymbol{Q}_{X}\left( 0 \right)$. | (A3) |
| --- | --- | --- |

The probability density that the transition *X_X'_* is completed at time *t* equals the change rate of *Q*_X_ at time *t*. Thus, the duration distribution of *X_X’_* with the initial probability ***Q***_X_(0) takes the following form:

|  | $f_{\mathrm{XX}^{'}}\left( t \right)=\boldsymbol{u}_{n_{X'}}\boldsymbol{K}_{\mathrm{XX}^{'}}e^{\boldsymbol{K}_{\mathrm{XX}}t}\boldsymbol{Q}_{X}\left( 0 \right)$, | (A4) |
| --- | --- | --- |

and the average duration of *X_X’_* is

|  | $\left\langle t_{\mathrm{XX}^{'}} \right\rangle=\boldsymbol{u}_{n_{X^{'}}}\boldsymbol{K}_{\mathrm{XX}^{'}}\boldsymbol{K}_{\mathrm{XX}}^{\boldsymbol{-2}}\boldsymbol{Q}_{X}\left( 0 \right)$, | (A5) |
| --- | --- | --- |

where ***u****_n_* is an 1$\times$*n* matrix with all elements being 1 and ***Q***_X_(0) = ***Q***_init_ is the probability of the states in *X* at the beginning. For *X_X'_*, the probabilities of the initial states are ***Q***_init_ = (*q_i_*); *q*_i_ *=* (*∑_j_k*_ji_*Q*_j_*^SS^*/*∑_s_k*_js_)/(∑*_i,j_k*_ji_*Q*_j_*^SS^*/*∑_s_k*_js_) if the state in set *X* comes directly from other sets, and *q*_i_ = 0 otherwise. Considering the whole transcription process, ***Q***(*t*) is the probability of all states at time *t* (***u****_l_****Q***(*t*) = 1), and the master equation for ***Q***(*t*) is

|  | $\frac{d\boldsymbol{Q}\left( t \right)}{dt}=\left( \boldsymbol{K}^{S}+\boldsymbol{K}^{P} \right)\boldsymbol{Q}\left( t \right)$. | (A6) |
| --- | --- | --- |

In steady state,

|  | $\left( \boldsymbol{K}^{S}+\boldsymbol{K}^{P} \right)\boldsymbol{Q}^{\mathrm{SS}}\boldsymbol{=0}$, | (A7) |
| --- | --- | --- |

which is a homogeneous linear equation, with ***Q***^SS^ **=** ($Q_{1}^{\mathrm{SS}}$, $Q_{2}^{\mathrm{SS}}$, $\cdots$, $Q_{l}^{\mathrm{SS}}$)^T^. Based on ***Q***^SS^ and Eqs. (A2-A5), the event model can be built.

First, for the process that begins with a state in set *I* and ends with $E_{I}^{P}$ or $I_{P}$, the probability ***Q*_1_**(*t*) that the states are still in the process after *t* satisfies

|  | $\frac{d\boldsymbol{Q}_{1}\left( t \right)}{dt}=\boldsymbol{A}_{\mathbf{1}}\boldsymbol{Q}_{\mathbf{1}}\left( t \right)$. | (A8) |
| --- | --- | --- |

Therefore,

|  | $\boldsymbol{Q}_{\mathbf{1}}\left( t \right)=e^{\boldsymbol{A}_{\mathbf{1}}t}\boldsymbol{Q}_{\mathbf{1}}\left( 0 \right)$, | (A9) |
| --- | --- | --- |

with $\boldsymbol{Q}_{\mathbf{1}}\left( 0 \right)=\left( \begin{matrix} \boldsymbol{0} & \boldsymbol{q}_{\mathbf{I}} \end{matrix} \right)^{T}$and $\boldsymbol{A}_{\mathbf{1}}=\left( \begin{matrix} \boldsymbol{K}_{\mathrm{EE}} & \boldsymbol{K}_{\mathrm{IE}} \\ \boldsymbol{K}_{\mathrm{EI}}^{\mathbf{S}} & \boldsymbol{K}_{\mathrm{II}} \end{matrix} \right)$. The duration distributions and average durations of *E_P_* and *E_S1_* are separately

|  | $f_{P}\left( t \right)=-\frac{\mathbf{u}\mathbf{A}_{\mathrm{EP}}e^{\mathbf{A}_{\mathbf{1}}t}\mathbf{Q}_{\mathbf{1}}\left( 0 \right)}{\mathbf{u}\mathbf{A}_{\mathrm{EP}}\mathbf{A}_{1}^{-1}\mathbf{Q}_{1}\left( 0 \right)}$ and $\tau_{P}=-\frac{\mathbf{u}\mathbf{A}_{\mathrm{EP}}\mathbf{A}_{1}^{-2}\mathbf{Q}_{\mathbf{1}}\left( 0 \right)}{\mathbf{u}\mathbf{A}_{\mathrm{EP}}\mathbf{A}_{1}^{-1}\mathbf{Q}_{1}\left( 0 \right)}$, | (A10) |
| --- | --- | --- |
|  | $f_{S1}\left( t \right)=-\frac{\mathbf{u}\mathbf{A}_{ES1}e^{\mathbf{A}_{\mathbf{1}}t}\mathbf{Q}_{\mathbf{1}}\left( 0 \right)}{\mathbf{u}\mathbf{A}_{ES1}\mathbf{A}_{1}^{-1}\mathbf{Q}_{1}\left( 0 \right)}$ and $\tau_{S1}=-\frac{\mathbf{u}\mathbf{A}_{ES1}\mathbf{A}_{1}^{-2}\mathbf{Q}_{\mathbf{1}}\left( 0 \right)}{\mathbf{u}\mathbf{A}_{ES1}\mathbf{A}_{1}^{-1}\mathbf{Q}_{1}\left( 0 \right)}$, | (A11) |

with ***A***_EP_ = ($\boldsymbol{K}_{\mathrm{EI}}^{P}$ **0**) and ***A***_ES1_ = (**0** ***K***_IA_). The probability of ending with $E_{I}^{P}$ is

|  | $p_{1}\mathbf{=}\int_{0}^{\boldsymbol{\infty}} J_{1}^{+}\left( t \right)\mathrm{dt}\mathbf{=-u}\mathbf{A}_{\mathrm{EP}}\mathbf{A}_{1}^{-1}\mathbf{Q}_{1}\left( 0 \right)=1-\int_{0}^{\infty} J_{1}^{-}\left( t \right)dt=1+\mathbf{u}\mathbf{A}_{ES1}\mathbf{A}_{1}^{-1}\mathbf{Q}_{1}\left( 0 \right)$, | (A12) |
| --- | --- | --- |

where $J_{1}^{+}$ and $J_{1}^{-}$ are the probability flows through $E_{I}^{P}$ and $A_{I}$, respectively, and are expressed as

|  | $J_{1}^{+}=\mathbf{u}\mathbf{A}_{\mathrm{EP}}\mathbf{A}_{\mathbf{1}}\mathbf{Q}_{\mathbf{1}}\left( t \right)$ and $J_{1}^{-}=\mathbf{u}\mathbf{A}_{ES1}\mathbf{A}_{\mathbf{1}}\mathbf{Q}_{\mathbf{1}}\left( t \right)$. | (A13) |
| --- | --- | --- |

For the process that begins with a state in set *A* and ends with *F_C_* or *A_I_*, the probability **Q_2_**(*t*) that the states are still in the process after *t* satisfies

|  | $\frac{d\mathbf{Q}_{2}\left( t \right)}{dt}=\mathbf{A}_{2}\mathbf{Q}_{2}\left( t \right)$, | (A14) |
| --- | --- | --- |

with $\mathbf{A}_{2}=\left( \begin{matrix} \mathbf{K}_{\mathrm{AA}} & \mathbf{K}_{\mathrm{FA}} & \mathbf{0} \\ \mathbf{K}_{\mathrm{AF}} & \mathbf{K}_{\mathrm{FF}} & \mathbf{K}_{\mathrm{CF}} \\ \mathbf{0} & \mathbf{0} & \mathbf{K}_{\mathrm{CC}} \end{matrix} \right)$ and **Q_2_**(0) = (***q***_A_ **0** **0**)^T^. Thus,

|  | $\mathbf{Q}_{2}\left( t \right)=e^{\mathbf{A}_{2}t}\mathbf{Q}_{2}\left( 0 \right)$. | (A15) |
| --- | --- | --- |

The duration distributions and average durations of *E_S2_* and *E*_end_ are separately

|  | $f_{S2}\left( t \right)=-\frac{\mathbf{u}\mathbf{A}_{ES2}e^{\mathbf{A}_{2}t}\mathbf{Q}_{2}\left( 0 \right)}{\mathbf{u}\mathbf{A}_{ES2}\mathbf{A}_{2}^{-1}\mathbf{Q}_{2}\left( 0 \right)}$ and $\tau_{S2}=-\frac{\mathbf{u}\mathbf{A}_{ES2}\mathbf{A}_{2}^{-2}\mathbf{Q}_{2}\left( 0 \right)}{\mathbf{u}\mathbf{A}_{ES2}\mathbf{A}_{2}^{-1}\mathbf{Q}_{2}\left( 0 \right)}$, | (A16) |
| --- | --- | --- |
|  | $f_{\mathrm{end}}\left( t \right)=-\frac{\mathbf{u}\mathbf{A}_{\mathrm{end}}e^{\mathbf{A}_{2}t}\mathbf{Q}_{2}\left( 0 \right)}{\mathbf{u}\mathbf{A}_{\mathrm{end}}\mathbf{A}_{2}^{-1}\mathbf{Q}_{2}\left( 0 \right)}$ and $\tau_{\mathrm{end}}=-\frac{\mathbf{u}\mathbf{A}_{\mathrm{end}}\mathbf{A}_{2}^{-2}\mathbf{Q}_{2}\left( 0 \right)}{\mathbf{u}\mathbf{A}_{\mathrm{end}}\mathbf{A}_{2}^{-1}\mathbf{Q}_{2}\left( 0 \right)}$, | (A17) |

where **A**_ES2_ = (**K**_AI_ **0** **0**) and **A**_end_ = (**0** **K**_FC_ **0**). The probability of ending with *F*_C_ is

|  | $p_{2}=\int_{0}^{\infty} J_{2}^{+}\left( t \right)dt=-\mathbf{u}\mathbf{A}_{\mathrm{end}}\mathbf{A}_{2}^{-1}\mathbf{Q}_{2}\left( 0 \right)=1-\int_{0}^{\infty} J_{2}^{-}\left( t \right)dt=1+\mathbf{u}\mathbf{A}_{ES2}\mathbf{A}_{2}^{-1}\mathbf{Q}_{2}\left( 0 \right)$, | (A18) |
| --- | --- | --- |

where $J_{2}^{+}$ and $J_{2}^{-}$ are the probability flows through *F*_C_ and *A_I_*, respectively, and are expressed as

|  | $J_{2}^{+}=\mathbf{u}\mathbf{A}_{\mathrm{end}}\mathbf{A}_{\mathbf{2}}\mathbf{Q}_{\mathbf{2}}\left( t \right)$ and $J_{2}^{-}=\mathbf{u}\mathbf{A}_{ES2}\mathbf{A}_{\mathbf{2}}\mathbf{Q}_{\mathbf{2}}\left( t \right)$. | (A19) |
| --- | --- | --- |

For the process that begins with a state in set *C* and ends with *F*_A_, the probability **Q_3_**(*t*) that the states are still in the process after *t* satisfies

|  | $\frac{d\mathbf{Q}_{3}\left( t \right)}{dt}=\mathbf{A}_{3}\mathbf{Q}_{3}\left( t \right)$, | (A20) |
| --- | --- | --- |

with $\mathbf{A}_{3}\boldsymbol{=}\left( \begin{matrix} \mathbf{K}_{\mathrm{FF}} & \mathbf{K}_{\mathrm{CF}} \\ \mathbf{K}_{\mathrm{FC}} & \mathbf{K}_{\mathrm{CC}} \end{matrix} \right)$ and $\mathbf{Q}_{3}\left( 0 \right)=\left( \begin{matrix} \boldsymbol{0} & \boldsymbol{q}_{C} \end{matrix} \right)^{T}$. Thus,

|  | $\mathbf{Q}_{3}\left( t \right)=e^{\mathbf{A}_{3}t}\mathbf{Q}_{3}\left( 0 \right)$. | (A21) |
| --- | --- | --- |

The duration distribution and average duration of *E*_begin_ are

|  | $f_{\mathrm{begin}}\left( t \right)=\mathbf{u}\mathbf{A}_{\mathrm{begin}}e^{\mathbf{A}_{3}t}\mathbf{Q}_{3}\left( 0 \right)$, $\tau_{\mathrm{begin}}=\mathbf{u}\mathbf{A}_{\mathrm{begin}}\mathbf{A}_{3}^{-2}\mathbf{Q}_{3}\left( 0 \right)$, | (A22) |
| --- | --- | --- |

with **A**_begin_ = (**K**_FI_ **0**). Thus, the duration distribution and average duration of *E*_S3_ take the following forms:

|  | $f_{ES3}\left( t \right)=\frac{\mathbf{u}\mathbf{A}_{\mathrm{end}}e^{\mathbf{A}_{2}t}\mathbf{Q}_{2}\left( 0 \right)}{\mathbf{u}\mathbf{A}_{\mathrm{end}}\mathbf{A}_{2}^{-1}\mathbf{Q}_{2}\left( 0 \right)}*\mathbf{u}\mathbf{A}_{\mathrm{begin}}e^{\mathbf{A}_{3}t}\mathbf{Q}_{3}\left( 0 \right)$, | (A23) |
| --- | --- | --- |
|  | $\tau_{S3}=-\frac{\mathbf{u}\mathbf{A}_{\mathrm{end}}\mathbf{A}_{2}^{-2}\mathbf{Q}_{2}\left( 0 \right)}{\mathbf{u}\mathbf{A}_{\mathrm{end}}\mathbf{A}_{2}^{-1}\mathbf{Q}_{2}\left( 0 \right)}+\mathbf{u}\mathbf{A}_{\mathrm{begin}}\mathbf{A}_{3}^{-2}\mathbf{Q}_{3}\left( 0 \right)$, | (A24) |

where “$*$” refers to convolution.

In addition, the transcription states consist of five state sets, implying that the process is a combination of subprocesses. We first consider the active process including mRNA production. $\mathbf{Q}_{A}\left( n;t \right)=\mathbf{Q}_{E\cup I}\left( n;t \right)$, i.e., the probability of states in set *E* and *I* at time *t* after $E_{I}^{P}$has occurred *n* times. **Q**_A_(*n*;0) = 0 for *n* ­> 0 and **Q**_A_(*n*; *t*) satisfies

|  | $\frac{d\mathbf{Q}_{A}\left( n;t \right)}{dt}=\mathbf{A}_{1}\mathbf{Q}_{A}\left( n;t \right)\boldsymbol{+}\mathbf{A}_{\mathrm{EP}}\mathbf{Q}_{A}\left( n-1;t \right)\boldsymbol{.}$ | (A25) |
| --- | --- | --- |

We calculate **Q**_A_(*n*; *t*) by introducing probability-generating functions of the vector form **G**(*z*; *t*) = (G_1_(*z*; *t*), G_2_(*z*; *t*), $\cdots$, $G_{n_{E}+n_{I}}\left( z;t \right)$)^T^ with G*_k_* (*z*; *t*) = $\sum_{n=0}^{\infty} z^{n}Q_{k}\left( n;t \right)$. Thus, we have the following partial differential equations:

|  | $\frac{\partial\mathbf{G}\left( z;t \right)}{\partial t}=\left( \mathbf{A}_{1}+z\mathbf{A}_{\mathrm{EP}} \right)\mathbf{G}\left( z;t \right)$, | (A26) |
| --- | --- | --- |
|  | $\mathbf{G}\left( z;t \right)\boldsymbol{=}e^{\left( \mathbf{A}_{1}+z\mathbf{A}_{\mathrm{EP}} \right)t}\mathbf{G}\left( z;0 \right)$. | (A27) |

We can solve for **Q**_A_(*n*;*t*)

|  | $\mathbf{Q}_{A}\left( n;t \right)\boldsymbol{=}\frac{1}{n!}\left. \left[ \frac{d^{n}}{dz^{n}}\mathbf{G}\left( z;t \right) \right] \right\vert_{z=0}\boldsymbol{=}\frac{1}{n!}\left. \left[ \frac{d^{n}}{dz^{n}}\sum_{i=0}^{\infty} \frac{1}{i!}\left( \mathbf{A}_{1}+z\mathbf{A}_{\mathrm{EP}} \right)^{i}t^{i} \right] \right\vert_{z=0}\mathbf{q}_{A}$, | (A28) |
| --- | --- | --- |

with **q**_A_ = (**0** **q**_I_)^T^ and **q**_I_ = (*q_i_*) (*q_i_* = $(\sum_{j\in A} k_{ji}Q_{\boldsymbol{j}}^{\mathbf{SS}}\boldsymbol{/}\sum_{s\in I} k_{js}) /(\sum_{i\in I} \sum_{j\in A} k_{ji}Q_{\boldsymbol{j}}^{\mathbf{SS}}/\sum_{s\in I} k_{js})$). By Laplace transform,

|  | $\mathcal{L}\left( \mathbf{Q}_{A}\left( n;t \right) \right)=\left( s\mathbf{I}-\mathbf{A}_{1} \right)^{-1}\left[ {\mathbf{A}_{\mathrm{EP}}\left( s\mathbf{I}-\mathbf{A}_{1} \right)}^{-1} \right]^{n}\mathbf{q}_{A}$. | (A29) |
| --- | --- | --- |

The duration distribution of the active process with *E*_S1_ followed is

|  | $f_{A+ES1}\left( t \right)=\frac{\mathbf{u}_{n_{A}}\mathbf{A}_{\mathrm{EP}}\sum_{n=1}^{\infty} \mathbf{Q}_{A}\left( n;t \right)}{p_{1}}.$ | (A30) |
| --- | --- | --- |

It can be rewritten as

|  | $f_{A+ES1}\left( t \right)=\frac{\mathcal{L}^{-1}\left( \mathbf{u}_{n_{A}}\mathbf{T}_{ES1}\sum_{n=1}^{\infty} \mathbf{T}_{\mathrm{EP}}^{n}\mathbf{q}_{A} \right)}{p_{1}}$, | (A31) |
| --- | --- | --- |

with **T**_ES1_ = **A**_ES1_(*s***I**-**A**_1_)^-1^ and **T**_EP_ = **A**_EP_(*s***I**-**A**_1_)^-1^. When a state dominates in set *I* or the states in set *I* are similar,

|  | $f_{A+ES1}\left( t \right)\approx f_{S1}\left( t \right)\sum_{n=1}^{\infty} \left( 1-p_{1} \right)p_{1}^{n-1}\left[ *f_{P}\left( t \right) \right]^{n}$. | (A32) |
| --- | --- | --- |

Thus,

|  | $f_{A}\left( t \right)\approx\sum_{n=1}^{\infty} \left( 1-p_{1} \right)p_{1}^{n-1}\left[ *f_{P}\left( t \right) \right]^{n}$. | (A33) |
| --- | --- | --- |

Similarly, the process that begins with a state in set *A* and ends with *A*_I_ consists of *E_S2_* and *E_S3_*. To obtain the relation between the process and the events, we make an equivalent transformation of the event model. After the transformation, **A** = (*A_i_*), **F** = (*F_i_*), **C** = (*C_i_*), where **A**, **F** and **C** are state sets of equivalent states. State transitions occur between *A*_2_*_j-_*_1_ and *F*_2_*_j-_*_1_, *F*_2_*_j_* and *C*_2_*_j_* (*j* = 1, 2, $\cdots$). The state in *C*_2_*_j-_*_1_ comes from *F*_2_*_j-_*_1_, and the state in *C*_2_*_j-_*_1_ only enters *F*_2_*_j_*. The state in *A*_2_*_j_* comes from *F*_2_*_j_*, and the state in *A*_2_*_j_* only enters *F*_2_*_j_*_+1_ or set *I*. The state in *A*_2_*_j-_*_1_ can go to *F*_2_*_j_*_-1_ or set *I*. **Q**_ES2+ES3_(*t*) = ($\boldsymbol{q}_{A_{1}}$, $\boldsymbol{q}_{F_{1}}$, $\boldsymbol{q}_{C}$, $\boldsymbol{q}_{A_{2}}$, $\boldsymbol{q}_{F_{2}}$, $\boldsymbol{q}_{C_{2}}$, $\cdots$)^T^ ($\boldsymbol{q}_{X_{i}}\left( 0 \right)$ = **0**; *X* = A, F and C; *i* = 1, 2, $\cdots$, except for *Xi* = *A*_1_) satisfies

|  | $\frac{d\mathbf{Q}_{ES2+ES3}\left( t \right)}{dt}=\mathbf{A}_{4}\mathbf{Q}_{ES2+ES3}\left( t \right)$, | (A34) |
| --- | --- | --- |

with

$\mathbf{A}_{4}\boldsymbol{=}\left( \begin{matrix} \boldsymbol{c} & \boldsymbol{0} & \boldsymbol{0} & \cdots\\ \boldsymbol{d} & \boldsymbol{c} & \boldsymbol{0} & \cdots\\ \boldsymbol{0} & \boldsymbol{d} & \boldsymbol{c} & \cdots\\ \boldsymbol{\vdots} & \boldsymbol{\vdots} & \boldsymbol{\vdots} & \ddots\end{matrix} \right)$**.**

Here, $\boldsymbol{c=}\left( \begin{matrix} \boldsymbol{a}_{1} & \boldsymbol{0} \\ \boldsymbol{b}_{1} & \boldsymbol{a}_{2} \end{matrix} \right)$ and $\boldsymbol{d=}\left( \begin{matrix} \boldsymbol{0} & \boldsymbol{b}_{2} \\ \boldsymbol{0} & \boldsymbol{0} \end{matrix} \right)$, with

| $\boldsymbol{a}_{1}\boldsymbol{=}\left( \begin{matrix} \mathbf{K}_{\mathrm{AA}} & \mathbf{K}_{\mathrm{FA}} & \boldsymbol{0} \\ \mathbf{K}_{\mathrm{AF}} & \mathbf{K}_{\mathrm{FF}} & \boldsymbol{0} \\ \boldsymbol{0} & \mathbf{K}_{\mathrm{FC}} & \mathbf{K}_{\mathrm{CC}} \end{matrix} \right)$, | $\boldsymbol{a}_{2}\boldsymbol{=}\left( \begin{matrix} \mathbf{K}_{\mathrm{AA}} & \mathbf{K}_{\mathrm{FA}} & \boldsymbol{0} \\ \mathbf{0} & \mathbf{K}_{\mathrm{FF}} & \mathbf{K}_{\mathrm{CF}} \\ \boldsymbol{0} & \mathbf{K}_{\mathrm{FC}} & \mathbf{K}_{\mathrm{CC}} \end{matrix} \right)$, |
| --- | --- |
| $\boldsymbol{b}_{1}=\left( \begin{matrix} \boldsymbol{0} & \boldsymbol{0} & \boldsymbol{0} \\ \boldsymbol{0} & \boldsymbol{0} & \mathbf{K}_{\mathrm{CF}} \\ \boldsymbol{0} & \boldsymbol{0} & \boldsymbol{0} \end{matrix} \right)$, | $\boldsymbol{b}_{2}=\left( \begin{matrix} \boldsymbol{0} & \boldsymbol{0} & \boldsymbol{0} \\ \mathbf{K}_{\mathrm{IF}} & \boldsymbol{0} & \mathbf{0} \\ \boldsymbol{0} & \boldsymbol{0} & \boldsymbol{0} \end{matrix} \right)$. |

The solution to Eq. (A34) is

|  | $\mathbf{Q}_{ES2+ES3}\left( t \right)\boldsymbol{=}e^{\boldsymbol{A}_{\boldsymbol{4}}\boldsymbol{t}}\mathbf{Q}_{ES2+ES3}\left( 0 \right)\boldsymbol{.}$ | (A35) |
| --- | --- | --- |

We obtain

|  | $f_{ES2+ES3}\left( t \right)=\mathbf{u}\mathbf{A}_{\mathrm{out}}e^{\mathbf{A}_{4}t}\mathbf{Q}_{ES2+ES3}\left( 0 \right)$, | (A36) |
| --- | --- | --- |
|  | $f_{S3}\left( t \right)\mathbf{=}\frac{\mathbf{u}\boldsymbol{d}e^{\boldsymbol{ct}}\boldsymbol{q}}{p_{2}}$, | (A37) |
|  | $f_{S2}\left( t \right)=\frac{\mathbf{u}\boldsymbol{a}_{out}e^{\boldsymbol{ct}}\boldsymbol{q}}{1-p_{2}}$, | (A38) |
|  | $p_{2}=-\mathbf{u}\boldsymbol{d}\boldsymbol{c}^{-1}\boldsymbol{q}$, | (A39) |

with $\mathbf{A}_{\mathrm{out}}$= ($\boldsymbol{a}_{\mathrm{out}}$ $\boldsymbol{a}_{\mathrm{out}}$ $\cdots$), $\boldsymbol{a}_{\mathrm{out}}$= ($\mathbf{K}_{\mathrm{AI}}$ $\boldsymbol{0}$ $\boldsymbol{0}$ $\mathbf{K}_{\mathrm{AI}}$ $\boldsymbol{0}$ $\boldsymbol{0}$) and $\boldsymbol{q}$ **=** ($\boldsymbol{q}_{A_{1}}$ $\boldsymbol{q}_{F_{1}}$ $\boldsymbol{q}_{C_{1}}$ $\boldsymbol{q}_{A_{2}}$ $\boldsymbol{q}_{F_{2}}$ $\boldsymbol{q}_{C_{2}}$)^T^. Thus,

|  | $f_{ES2+ES3}\left( t \right)\approx f_{S2}\left( t \right)\sum_{n=0}^{\infty} \left( 1-p_{2} \right)p_{2}^{n}\left[ *f_{S3}\left( t \right) \right]^{n}$. | (A40) |
| --- | --- | --- |

On the whole,

|  | $f_{S}\left( t \right)=f_{S1}\left( t \right)*f_{S2}\left( t \right)\sum_{n=0}^{\infty} \left( 1-p_{2} \right)p_{2}^{n}\left[ *f_{S3}\left( t \right) \right]^{n}$, | (A41) |
| --- | --- | --- |
|  | $f_{I}\left( t \right)=\delta\left( t \right)\sum_{n=1}^{\infty} {p_{1}\left( 1-p_{1} \right)}^{n-1}\left[ *f_{S}\left( t \right) \right]^{n}$, | (A42) |
|  | $f_{A}\left( t \right)=\delta\left( t \right)\sum_{n=1}^{\infty} \left( 1-p_{1} \right)p_{1}^{n-1}\left[ *f_{P}\left( t \right) \right]^{n}$, | (A43) |
|  | $f_{T}\left( t \right)=f_{A}\left( t \right)*f_{I}\left( t \right)$, | (A44) |

where *f*_S_ is the duration distribution of the process from *E*_S1_ to *E*_S2,_ *f*_A_ (*f*_I_) is that of the active (inactive) phase, and *f*_T_ is that of a burst.

**Section 2: Structure of the event model**

The entire process of transcription can be understood analytically by breaking it down into basic Poisson events. $f(t)$ is the duration distribution of any process, which takes the following form:

$f\left( t \right)=\sum_{i=1}^{n} P_{i}f_{i}\left( t \right),$ (B1)

with $\sum_{i=1}^{n} P_{i}=1$, $f_{i}\left( t \right)=\sum_{j=1}^{n_{i}} \frac{\prod_{s=1}^{n_{i}} k_{s}}{\prod_{s\neq j} \left( k_{s}-k_{j} \right)}e^{-k_{j}t}$, $\tau_{s}=\frac{1}{k_{s}}$, $\left\langle t_{i} \right\rangle=\sum_{j=1}^{n_{i}} \tau_{j}$ and $\sigma_{i}^{2}=\sum_{j=1}^{n_{i}} \tau_{j}^{2}$, where $0<P_{i}<1$ and $k_{s}$ is the reaction rate. For two sequential processes, the duration distribution of the total process, $f_{t}(t)$, can be expressed as

$f_{t}\left( t \right)=f_{a}\left( t \right)*f_{b}\left( t \right)=\sum_{i=1}^{n_{a}} \sum_{j=1}^{n_{b}} P_{i}P_{j}f_{i}\left( t \right)*f_{j}\left( t \right)$, (B2)

with $f_{a}\left( t \right)=\sum_{i=1}^{n_{a}} P_{i}f_{i}\left( t \right)$ and $f_{b}\left( t \right)=\sum_{j=1}^{n_{b}} P_{j}f_{j}\left( t \right).$ $f_{t}\left( t \right)=f_{a}\left( t \right)*f_{b}\left( t \right)=\int_{0}^{t} f_{a}\left( \tau\right)f_{b}\left( t-\tau\right)d\tau$. Their sub-processes can determine the characteristics of the total process.

$$\left\langle t \right\rangle=\sum_{i=1}^{n_{a}} \sum_{j=1}^{n_{b}} P_{i}P_{j}(\left\langle t_{i} \right\rangle+\left\langle t_{j} \right\rangle)=\left\langle t_{a} \right\rangle+\left\langle t_{b} \right\rangle$$

$$\left\langle t^{2} \right\rangle=\sum_{i=1}^{n_{a}} \sum_{j=1}^{n_{b}} P_{i}P_{j}\left[ \sum_{\alpha=1}^{n_{i}} \tau_{\alpha}^{2}+\sum_{\beta=1}^{n_{j}} \tau_{\beta}^{2}+\left( \sum_{\alpha=1}^{n_{i}} \tau_{\alpha}+\sum_{\beta=1}^{n_{j}} \tau_{\beta} \right)^{2} \right]=\left\langle t_{a} \right\rangle^{2}+\left\langle t_{b} \right\rangle^{2}+2\langle t_{a}\rangle\langle t_{b}\rangle$$

$\sigma_{t}^{2}=\sigma_{a}^{2}+\sigma_{b}^{2}$*.*

Thus,

$$\alpha_{t}=\frac{\left( \left\langle t_{a} \right\rangle+\left\langle t_{b} \right\rangle\right)^{2}}{\sigma_{a}^{2}+\sigma_{b}^{2}}=\frac{1}{\frac{1}{\alpha_{a}}\left( \frac{r}{r+1} \right)^{2}+\frac{1}{\alpha_{b}}\left( \frac{1}{r+1} \right)^{2}}, r=\frac{\left\langle t_{a} \right\rangle}{\left\langle t_{b} \right\rangle}$$

$\mathrm{with} \min\left( \alpha_{a},\alpha_{b} \right)\leq\alpha_{t}\leq\alpha_{a}+\alpha_{b}$. When $r$ is small (large), $\alpha_{t}$ tends to be $\alpha_{a}$ ($\alpha_{b}$). Only for$r=\frac{\alpha_{a}}{\alpha_{b}}$, $\alpha_{t}$ takes the maximum of $\alpha_{a}+\alpha_{b}$ [Fig. S2].

In fact, there are many parallel processes besides sequential processes. We consider a composite process around the promoter, whose duration distribution is

$f\left( t \right)=\left( 1-p \right)u\left( t \right)+\sum_{i=1}^{\infty} \left( 1-p \right)p^{i}u\left( t \right)\left[ *w\left( t \right) \right]^{i}=\left( 1-p \right)u\left( t \right)+pu\left( t \right)*L\left( t \right).$ (B3)

Here$u\left( t \right)\left[ *w\left( t \right) \right]^{i}$ = $u\left( t \right)*w\left( t \right)*\cdots*w\left( t \right)$ (convoluting $w\left( t \right)$ *i* times) and $L\left( t \right)$= $\frac{1-p}{p}\sum_{i=1}^{\infty} p^{i}\delta\left( t \right)\left[ *w\left( t \right) \right]^{i}$. Thus, $\left\langle t_{L} \right\rangle=\frac{1}{1-p}\left\langle t_{w} \right\rangle$, $\alpha_{L}=\frac{1}{p+\frac{1}{\alpha_{w}}\left( 1-p \right)}$, $\left\langle t \right\rangle=\left\langle t_{u} \right\rangle+\frac{p}{1-p}\left\langle t_{w} \right\rangle$ and $\alpha_{t}=\left[ \frac{1}{\alpha_{u}}\left( \frac{r}{r+\frac{p}{1-p}} \right)^{2}+{\left( \frac{1-p}{p}\frac{1}{\alpha_{w}}+\frac{1}{p} \right)\left( \frac{\frac{p}{1-p}}{r+\frac{p}{1-p}} \right)}^{2} \right]^{-1}.$ It is easy to know $1\leq\alpha_{L}<\alpha_{u}$ and $\alpha_{t}<\alpha_{u}+1$, meaning that the upper limit of $\alpha_{t}$ is decided by $\alpha_{u}$ ($r=\frac{\left\langle t_{u} \right\rangle}{\langle t_{w}\rangle}$). $L\left( t \right)$ tends to be an exponential distribution when $p$ is large (close to 1). Given large $p$, $\alpha_{t}$ of $f\left( t \right)$ tends to be $\alpha_{u}+1$ for large $\frac{\tau_{u}}{\tau_{L}}$, whereas $\alpha_{t}$ tends to be 1 for small $\frac{\tau_{u}}{p\tau_{L}}$ ($\tau_{u}$ and $\tau_{L}$ are separately the average durations of $u\left( t \right)$ and $L\left( t \right)$) [Fig. S3].

In the event model, the whole transcription process can be divided into active and inactive phases according to the bursting kinetics [1]. The distribution of the active phase duration is $f_{A}\left( t \right)$:

$f_{A}\left( t \right)=\sum_{i=0}^{\infty} \left( 1-p_{1} \right)p_{1}^{i}\delta\left( t \right)\left[ *f_{P}\left( t \right) \right]^{i+1}$ (B4)

with $\left\langle t_{A} \right\rangle=\frac{1}{1-p_{1}}\langle t_{P}\rangle$ and $\alpha_{A}=\frac{1}{p_{1}+\left( 1-p_{1} \right)\frac{1}{\alpha_{P}}}$. $f_{P}\left( t \right)$ is the distribution of interval between two adjacent Pol II entering elongation. $p_{1}$is the probability that Pol II is recruited to produce a transcript. For large burst size, $p_{1}$ is large and $\alpha_{A}$ of$f_{A}\left( t \right)$ is close to 1, which means $f_{A}\left( t \right)$ is close to an exponential distribution. Different from$f_{A}\left( t \right)$, the duration distribution of inactive interval,$f_{I}\left( t \right)$, is expressed as

$f_{I}\left( t \right)=\sum_{i=0}^{\infty} p_{1}\left( 1-p_{1} \right)^{i}\delta\left( t \right)\left[ *f_{S}\left( t \right) \right]^{i+1},$ (B5)

where

$f_{S}\left( t \right)=f_{S1}\left( t \right)*f_{S1}\left( t \right)\sum_{i=0}^{\infty} \left( 1-p_{2} \right)p_{2}^{i}\left[ *f_{S3}\left( t \right) \right]^{i}$ (B6)

and $p_{2}$ is the probability that the gene state enters set *C* from set *F*. When $p_{1}$ is close to one, $f_{I}\left( t \right)$ is similar to$f_{S}\left( t \right)$. The average duration of $f_{I}\left( t \right)$ is $\left\langle t_{I} \right\rangle=\frac{1}{p_{1}}\langle t_{S}\rangle$, with $\left\langle t_{S} \right\rangle=\left\langle t_{S1} \right\rangle+\left\langle t_{S2} \right\rangle+\frac{p_{2}}{1-p_{2}}\langle t_{S3}\rangle$. Accordingly, $\alpha_{I}=\frac{1}{\left( 1-p_{1} \right)+p_{1}\frac{1}{\alpha_{S}}}$, with

$\alpha_{S}=\left[ \left( \frac{r}{r+\frac{p_{2}}{1-p_{2}}} \right)^{2}\frac{1}{\alpha_{S12}}+\left( \frac{\frac{p_{2}}{1-p_{2}}}{r+\frac{p_{2}}{1-p_{2}}} \right)^{2}\left( \frac{1-p_{2}}{p_{2}}\frac{1}{\alpha_{S3}}+\frac{1}{p_{2}} \right) \right]^{-1}$, $r=\frac{\left\langle t_{S1} \right\rangle+\langle t_{S2}\rangle}{\langle t_{S3}\rangle}$.

We put the conclusions above into our model and make clear that $\alpha_{I}$ is close to 1 when $\tau_{I1}<\tau_{I2}$ and *α*_I_ is large when $\tau_{I1}>\tau_{I2}$. $\tau_{I1}$ is the average total duration of *E*_S1_ and *E*_S2_ in a burst and equals $\frac{1}{p_{1}}\left( \tau_{S1}+\tau_{S2} \right)$. $\tau_{I2}$ is the average total duration of *E*_S3_ in a burst and equals $\frac{1}{p_{1}}\frac{p_{2}}{1-p_{2}}\tau_{S3}$.

**Section 3: Simulation of the event model and phenomenological models**

To simulate the transcription with the event model, we first assume the gene is poised to undergo *E*_S3_. After *E*_S3_, *E*_S3_ recurs when *r_i_* ≤ *p*_2_ until *E*_S2_ occurs with *r_i_* > *p*_2_. *r_i_* is a random number between 0 and 1 generated after each event ($i$ is the number of events that have occurred). After *E*_S2_, *E*_P_ occurs when *r_i_* ≤ *p*_1_; otherwise *E*_S1_ occurs. After *E*_P_, *E*_P_ recurs when *r_i_* ≤ *p*_1_ until *E*_S1_ occurs with *r_i_* > *p*_1_. After *E*_S1_, *E*_S3_ occurs when *r_i_* ≤ *p*_2_; otherwise *E*_S2_ appears. The time after the *i*th event is *t_i_*, equaling *t_i-_*_1_+*τ*_i_. *τ*_i_ is the time taken to undergo the *i*th event. The time taken to complete *E*_X_ is *F*_X_^-1^(*r*_i_) (X = P, S1, S2, S3), where *F*_X_^-1^ is the inverse function of $F_{X}\left( t \right)=\int_{0}^{t} f_{X}(x)dx$ and *f*_X_(*x*) is the duration function of *E*_X_. *t*_pj_ is the time at which the *j*th mRNA is produced after *E*_P_, and *t*_pj_+*t*_dj_ is the time at which the *j*th mRNA degrades ($t_{\mathrm{dj}}=\frac{1}{\tau_{m}}\ln\frac{1}{r_{j}}$; $r_{j}$ is a random number between 0 and 1). The number of mRNA transcripts is $m\left( t \right)=\sum_{t_{\mathrm{pj}}\leq t} \varepsilon\left( t-t_{\mathrm{pj}} \right)-\sum_{t_{\mathrm{pj}}+t_{\mathrm{dj}}\leq t} \varepsilon\left( t-t_{\mathrm{pj}}-t_{\mathrm{dj}} \right)$, where *ε*(*t*) is a step function.

There are many phenomenological models of transcription. Here, we take four examples (the telegraph model, multi-ON model, multi-OFF model and multi-scale model) to illustrate how they are described within the current framework. The biochemical master equation describing the synthesis of mRNA is as follows:

$\frac{d\mathbf{P}\left( m;t \right)}{dt}=\mathbf{AP}\left( m;t \right)+\delta_{m}\left( \mathbf{E}-\mathbf{I} \right)\left[ m\mathbf{P}\left( m;t \right) \right]+\boldsymbol{\Lambda}\left( \mathbf{E}^{\mathbf{-1}}-\mathbf{I} \right)\mathbf{P}\left( m-1;t \right)$, (C1)

where $\mathbf{P}\left( m;t \right)\mathbf{=}\left( P_{1}\left( m;t \right),P_{2}\left( m;t \right),\cdots,P_{n}(m,t) \right)^{\mathbf{T}}$ is the probability of gene state with $m$ transcripts at time $t$ ($n$ is the number of gene states). $\mathbf{A}\boldsymbol{=(}a_{\mathrm{ij}}\boldsymbol{)}$ is an $n\times n$ matrix and $a_{\mathrm{ij}}$ is the transformation rate from $P_{j}(m,t)$ to $P_{i}(m,t)$. $\boldsymbol{\Lambda}\boldsymbol{=(}b_{\mathrm{ij}}\boldsymbol{)}$ is an $n\times n$ matrix, where $b_{ii}$ is the transcription rate and *b_ij_* = 0 ($i\neq j$). $\boldsymbol{E}$ is a shift operator and $\mathbf{I}$ is the identity operator.$\delta_{m}$ is the degradation rate constant of mRNA.

For the telegraph model,

$\mathbf{A}\boldsymbol{=}\left( \begin{matrix} -k_{\mathrm{off}} & k_{\mathrm{on}} \\ k_{\mathrm{off}} & -k_{\mathrm{on}} \end{matrix} \right)$**,** $\boldsymbol{\Lambda=}\left( \begin{matrix} k_{m} & 0 \\ 0 & 0 \end{matrix} \right)\boldsymbol{.}$

The model can be described using $p_{1}=\frac{k_{m}}{k_{\mathrm{off}}+k_{m}}, f_{P}\left( t \right)=\left( k_{\mathrm{off}}+k_{m} \right)e^{-\left( k_{\mathrm{off}}+k_{m} \right)t},{\mathrm{and} f}_{I}\left( t \right)=k_{\mathrm{on}}e^{-k_{\mathrm{on}}t}$, because the duration distribution of the inactive state is independent of the transcription rate.

For the multi-on model,

$\mathbf{A}\boldsymbol{=}\left( \begin{matrix} -k_{1,2} & 0 & \ldots& 0 & k_{n,1} \\ k_{1,2} & -k_{2,3} & \ldots& 0 & 0 \\ \vdots& \vdots& \ddots& \vdots& \vdots\\ 0 & 0 & \ldots& -k_{n-1,n} & 0 \\ 0 & 0 & \ldots& k_{n-1,n} & -k_{n,1} \end{matrix} \right)$**,** $\boldsymbol{\Lambda=}\left( \begin{matrix} k_{m,1} & 0 & \ldots& 0 & 0 \\ 0 & k_{m,2} & \ldots& 0 & 0 \\ \vdots& \vdots& \ddots& \vdots& \vdots\\ 0 & 0 & \ldots& k_{m,1} & 0 \\ 0 & 0 & \ldots& 0 & 0 \end{matrix} \right)$**.**

The model can be described using $p_{1,i}=\frac{k_{m,i}}{k_{i,i+1}+k_{m,i}}, f_{Pi}\left( t \right)=\left( k_{i,i+1}+k_{m} \right)e^{-\left( k_{i,i+1}+k_{m} \right)t} \left( i=1, 2,\cdots,n-1 \right),{\mathrm{and} f}_{I}\left( t \right)=k_{n1}e^{-k_{n1}t}$.

For the multi-off model,

$\mathbf{A}\boldsymbol{=}\left( \begin{matrix} -k_{1,2} & 0 & \ldots& 0 & k_{n,1} \\ k_{1,2} & -k_{2,3} & \ldots& 0 & 0 \\ \vdots& \vdots& \ddots& \vdots& \vdots\\ 0 & 0 & \ldots& -k_{n-1,n} & 0 \\ 0 & 0 & \ldots& k_{n-1,n} & -k_{n,1} \end{matrix} \right)$**,** $\boldsymbol{\Lambda=}\left( \begin{matrix} k_{m} & 0 & \ldots& 0 & 0 \\ 0 & 0 & \ldots& 0 & 0 \\ \vdots& \vdots& \ddots& \vdots& \vdots\\ 0 & 0 & \ldots& 0 & 0 \\ 0 & 0 & \ldots& 0 & 0 \end{matrix} \right)$**.**

The model can be described using $p_{1}=\frac{k_{m}}{k_{m}+k_{1,2}}, f_{P}\left( t \right)=\left( k_{1,2}+k_{m} \right)e^{-\left( k_{12}+k_{m} \right)t},$and $f_{I}\left( t \right)=\sum_{i=2}^{n} \frac{\prod_{j=2}^{n} k_{j,j+1}}{\prod_{j\neq i}^{n} \left( k_{j,j+1}-k_{i,i+1} \right)}e^{-k_{i,i+1}t}\left( k_{n,n+1}=k_{n,1} \right)$.

For the multi-scale model,

$\mathbf{A}\boldsymbol{=}\left( \begin{matrix} {-k}_{12} & k_{21} & 0 & 0 \\ k_{12} & -(k_{21}+k_{23}+k_{24}) & k_{32} & k_{42} \\ 0 & k_{23} & -k_{32} & 0 \\ 0 & k_{24} & 0 & -k_{43} \end{matrix} \right)$**,** $\boldsymbol{\Lambda=}\left( \begin{matrix} k_{m} & 0 & 0 & 0 \\ 0 & 0 & 0 & 0 \\ 0 & 0 & 0 & 0 \\ 0 & 0 & 0 & 0 \end{matrix} \right)$**.**

The model can be described using $p_{1}=\frac{k_{m}}{k_{m}+k_{1,2}},f_{P}\left( t \right)=\left( k_{1,2}+k_{m} \right)e^{-\left( k_{12}+k_{m} \right)t},$and $f_{I}\left( t \right)=\mathbf{A}\left( 1,2:4 \right)e^{\boldsymbol{A}\left( 2:4,2:4 \right)t}\left( \begin{aligned} 1 \\ 0 \\ 0 \end{aligned} \right)$.

We simulate the telegraph model using the Gillespie algorithm [2,3]. We use *E*_P_, *E*_S_ (including *E*_S1_, *E*_S2_ and *E*_S3_) and $p_{1}$ to simulate the transcription. *E*_P_ occurs after *E*_S_. After *E*_P_, *E*_P_ recurs when *r* ≤ *p*_1_ until *E*_S_ occurs with *r* > *p*_1_ (*r* is a random number between 0 and 1). The phenomenological models and the event model show the same average and similar dynamics. However, there exists a marked difference because of different definitions of the active phase. At least one mRNA is produced during the active phase in the event model, whereas there may be no mRNA in the others. Therefore, the variance in mRNA count is markedly different when *p*_1_=1-*k*_off_/*k*_m_ is small or $\tau_{A}\sim\tau_{I}$ [Fig. S9]. When the bursting parameters (average duration of the active (inactive) phase (i.e., 1/*k*_off_ and 1/*k*_on_) and transcription rate constant (*k*_m_)) are regulated, the difference between the models is small when $m$ is small or large, whereas it is obvious for intermediate $m$ [Fig. [S9A-](#FIGS8)C)]. Experimentally, the active phase begins with mRNA synthesis (a fluorescence signal is generated), which is close to the definition in the event model.

**Section 4:** **Regulation of transcriptional bursting**

Regulators modulate gene transcription in diverse manners. It is necessary to unravel how the concentration of regulators regulates the duration of active and inactive phases. The binding of regulators alters the reaction rate and the probability of selecting the reaction. Assume that the reaction is affected by *N* regulators (*R*_1_, *R*_2_, $\cdots$, *R_N_*) and the number of binding sites for *R*_i_ is *n_i_*, the probability of gene state is written as ***P =*** (***P_b_***), where ***b*** is a vector representing the regulator binding state and equals $\left( \begin{matrix} b_{11} & b_{12} & \cdots& b_{1n_{1}} & \cdots& b_{ij} & \cdots& b_{N1} & \cdots& b_{Nn_{N}} \end{matrix} \right)$. *b_ij_* = 1 represents the case where *R_i_* is bound to the *j*th binding site, whereas *b_ij_* = 0 represents the case where *R_i_* is not. For any reaction, we have a matrix ***K***, with $\boldsymbol{K}_{\boldsymbol{i}_{\boldsymbol{b}}\boldsymbol{i}_{\boldsymbol{b}}}\boldsymbol{=}\boldsymbol{K}_{\boldsymbol{b}}\boldsymbol{-}\sum_{\boldsymbol{b}^{\boldsymbol{'}}} \left( k_{\boldsymbol{b}\boldsymbol{b}^{\boldsymbol{'}}}\boldsymbol{+}k_{\boldsymbol{b}^{\boldsymbol{'}}\boldsymbol{b}} \right)\boldsymbol{I}$*,* $\boldsymbol{K}_{\boldsymbol{i}_{\boldsymbol{b}}\boldsymbol{i}_{\boldsymbol{b}^{\boldsymbol{'}}}}\boldsymbol{=}k_{\boldsymbol{b}\boldsymbol{b}^{\boldsymbol{'}}}\boldsymbol{I}$ and $\boldsymbol{K}_{\boldsymbol{i}_{\boldsymbol{b}^{\boldsymbol{'}}}\boldsymbol{i}_{\boldsymbol{b}}}\boldsymbol{=}k_{\boldsymbol{b}^{\boldsymbol{'}}\boldsymbol{b}}\boldsymbol{I}$. $\boldsymbol{K}_{\boldsymbol{b}}$ is the reaction rate matrix associated with binding state ***b***. $k_{\boldsymbol{b}^{\boldsymbol{'}}\boldsymbol{b}}$ is the reaction rate for the change from $\boldsymbol{b}^{\boldsymbol{'}}$to ***b*,** and $k_{\boldsymbol{b}\boldsymbol{b}^{\boldsymbol{'}}}$ is from ***b*** to $\boldsymbol{b}^{\boldsymbol{'}}$. Therefore,

|  | $\boldsymbol{uKP}\mathbf{=}\sum_{\boldsymbol{b}} \boldsymbol{K}_{\boldsymbol{b}}\boldsymbol{P}_{\boldsymbol{b}}\boldsymbol{=}\left( \sum_{\boldsymbol{b}} \boldsymbol{K}_{\boldsymbol{b}}\boldsymbol{\omega}_{\boldsymbol{b}} \right)\boldsymbol{p=kp}$, | (D1) |
| --- | --- | --- |

with $\boldsymbol{p=}\sum_{\boldsymbol{b}} \boldsymbol{P}_{\boldsymbol{b}}$, $\boldsymbol{\omega}_{\boldsymbol{b}}\boldsymbol{p=}\boldsymbol{P}_{\boldsymbol{b}}$ ($\boldsymbol{\omega}_{\boldsymbol{b}}$ is a diagonal matrix) and $\boldsymbol{k=}\sum_{\boldsymbol{b}} \boldsymbol{K}_{\boldsymbol{b}}\boldsymbol{\omega}_{\boldsymbol{b}}$. Given the dwell time of regulators bound at specific binding sites is short (1-100 sec), we have

|  | $\boldsymbol{\omega}_{\boldsymbol{b}}\approx\frac{\frac{1}{c_{\boldsymbol{b}}}\prod_{i=1}^{N} \prod_{j=1}^{n_{i}} \left( \frac{\left[ R_{i} \right]}{K_{di}} \right)^{b_{ij}}}{\sum_{\boldsymbol{b}} \frac{1}{c_{\boldsymbol{b}}}\prod_{i=1}^{N} \prod_{j=1}^{n_{i}} \left( \frac{\left[ R_{i} \right]}{K_{di}} \right)^{b_{ij}}}\boldsymbol{I,}$ | (D2) |
| --- | --- | --- |

where *c****_b_*_­_** is the binding cooperativity that confers additional stability.

Eq. ([A1](file:///G:\work1\投稿\ReviewComments\v1\Wu-SI%20-%20v1.docx#EqA1)) can be rewritten as

|  | $\frac{d\boldsymbol{P}\left( m;t \right)}{dt}=\mathbf{K}^{S}\mathbf{P}\left( m;t \right)+\delta_{m}\left( \mathbf{E}-\mathbf{I} \right)\left[ m\mathbf{P}\left( m;t \right) \right]+\mathbf{K}^{P}\mathbf{P}\left( m-1;t \right)$, | (D3) |
| --- | --- | --- |

where $\mathbf{P}\left( m;t \right)$ = $\sum_{\boldsymbol{b}} \boldsymbol{\omega}_{\boldsymbol{b}}P_{\boldsymbol{b}}\left( m;t \right)$, $\mathbf{K}^{S}$= $\sum_{\boldsymbol{b}} \boldsymbol{\omega}_{\boldsymbol{b}}\mathbf{K}_{\boldsymbol{b}}^{S}$, and $\mathbf{K}^{P}=\sum_{\boldsymbol{b}} \boldsymbol{\omega}_{\boldsymbol{b}}\mathbf{K}_{\boldsymbol{b}}^{P}$. When a signal (*s*) affects multiple regulators, the concentration of *R_i_* in steady state is a function of *s*, denoted as [*R*_i_](*s*). [*R*_i_](*s*) can be written in polynomial form, leading to

|  | $\boldsymbol{k=}\sum_{\boldsymbol{b}} \boldsymbol{K}_{\boldsymbol{b}}\boldsymbol{\omega}_{\boldsymbol{b}}\boldsymbol{=}\frac{\sum_{i=0} {\boldsymbol{k}_{\boldsymbol{i}}\left( \frac{s}{K_{i}} \right)}^{i}}{\sum_{i=0} \left( \frac{s}{K_{i}} \right)^{i}}$. | (D4) |
| --- | --- | --- |

When matrix elements of $\boldsymbol{k}$ exhibit saturation with increasing *s*, *k*_ij_ can be written as

|  | $k_{ij}\boldsymbol{\approx}p_{o,ij}k_{0,ij}+\left( 1-p_{o,ij} \right)k_{1,ij}$, | (D5) |
| --- | --- | --- |

where *p*_o_*_,ij_* is the effective regulator occupancy rate ($\frac{s^{n_{ij}}}{K_{dij}^{n_{ij}}+s^{n_{ij}}}$), while *k*_0_*_,ij_* and *k*_1_*_,ij_* are the reaction rates for *p*_o_*_,ij_* = 0 and *p*_o_*_,ij_* = 1, respectively. When only the concentration of one regulator (*R*) changes with the others remaining constant, $p_{o}\approx\frac{\left[ R \right]^{n_{H}}}{K_{H}^{n_{H}}+\left[ R \right]^{n_{H}}}$. With the single-binding site assumption, we have $p_{o}=\frac{\left[ R \right]}{K_{d}+\left[ R \right]}$, *k*_0_*_,ij_* = *k*_U_ and *k*_1_*_,ij_* = *k*_B_**,** where *k*_B_ and *k*_U_ are the reaction rate matrixes with or without regulators bound.

According to Eqs. ([A12](#EqA12)) and ([A18](#EqA18)), *p*_i_ ($i=1, 2$) can be expressed as

|  | $p_{i}=-\mathbf{u}\mathbf{A}_{out,1}\mathbf{A}^{-\mathbf{1}}\mathbf{Q}=1+\mathbf{u}\mathbf{A}_{out,2}\mathbf{A}^{-\mathbf{1}}\mathbf{Q}$. | (D6) |
| --- | --- | --- |

When regulators act on **A**_out,1_,

|  | $1-p_{i}=-\mathbf{u}\mathbf{A}_{out,2}\left[ p_{o}\mathbf{A}_{B}+\left( 1-p_{o} \right)\mathbf{A}_{U} \right]^{-\mathbf{1}}\mathbf{Q}$, | (D7) |
| --- | --- | --- |

where *p*_o_ is the effective occupancy rate of regulators. Assuming **A**_B_ = **TA**_U_, we have

|  | $1-p_{i}=-\frac{1}{1-p_{o}}\mathbf{u}\mathbf{A}_{out,2}\mathbf{A}_{U}^{-\mathbf{1}}\left[ \mathbf{I}+\frac{p_{o}}{1-p_{o}}\mathbf{T} \right]^{-\mathbf{1}}\mathbf{Q}$, | (D8) |
| --- | --- | --- |
|  | $\frac{1}{1-p_{i}}=\left( 1-p_{o} \right)\frac{1}{-\mathbf{u}\mathbf{A}_{out,2}\mathbf{A}_{U}^{-\mathbf{1}}\left[ \mathbf{I}+\frac{p_{o}}{1-p_{o}}\mathbf{T} \right]^{-\mathbf{1}}\mathbf{Q}}$. | (D9) |

By using Taylor expansion and discarding higher order terms, Eq. (D9) can be approximated as

|  | $\frac{1}{1-p_{i}}\approx\left( 1-p_{o} \right)\frac{1}{-\mathbf{u}\mathbf{A}_{out,2}\mathbf{A}_{U}^{\mathbf{-1}}\mathbf{Q}}+p_{o}\frac{1}{-\mathbf{u}\mathbf{A}_{out,2}\mathbf{A}_{U}^{-\mathbf{1}}\mathbf{T}^{-\mathbf{1}}\mathbf{Q}}$, | (D10) |
| --- | --- | --- |
|  | $\frac{1}{1-p_{i}}\approx\left( 1-p_{o} \right)\frac{1}{1-p_{\mathrm{iU}}}+p_{o}\frac{1}{1-p_{\mathrm{iB}}}$. | (D11) |

When regulators act on **A**_out,2_,

|  | $p_{i}=-\mathbf{u}\mathbf{A}_{out,1}\left[ p_{o}\mathbf{A}_{B}+\left( 1-p_{o} \right)\mathbf{A}_{U} \right]^{\mathbf{-1}}\mathbf{Q}$. | (D12) |
| --- | --- | --- |

Similarly,

|  | $\frac{1}{p_{i}}\approx\left( 1-p_{o} \right)\frac{1}{p_{\mathrm{iU}}}+p_{o}\frac{1}{p_{\mathrm{iB}}}$. | (D13) |
| --- | --- | --- |

According to Eqs. ([A10](#EqA10), [A11](#EqA11), [A16](#EqA16), [A17](#EqA17), [A22](#EqA22) and [A24](#EqA24)), *τ_j_* (*j* = P, S1, S2, S) can be expressed as

|  | $\tau_{j}=-\frac{\mathbf{u}\mathbf{A}_{\mathrm{out}}\mathbf{A}^{-\mathbf{2}}\mathbf{Q}}{\mathbf{u}\mathbf{A}_{\mathrm{out}}\mathbf{A}^{-\mathbf{1}}\mathbf{Q}}$, | (D14) |
| --- | --- | --- |
|  | $\tau_{j}=-\frac{\mathbf{u}\left[ p_{o}\mathbf{T}_{1}\mathbf{A}_{\mathrm{outU}}+\left( 1-p_{o} \right)\mathbf{A}_{\mathrm{outU}} \right]\left[ p_{o}\mathbf{T}_{\mathbf{2}}\mathbf{A}_{U}+\left( 1-p_{o} \right)\mathbf{A}_{U} \right]^{-\mathbf{2}}\mathbf{Q}}{\mathbf{u}\left[ p_{o}\mathbf{T}_{1}\mathbf{A}_{\mathrm{outU}}+\left( 1-p_{o} \right)\mathbf{A}_{\mathrm{outU}} \right]\left[ p_{o}\mathbf{T}_{\mathbf{2}}\mathbf{A}_{U}+\left( 1-p_{o} \right)\mathbf{A}_{U} \right]^{-\mathbf{1}}\mathbf{Q}}$, | (D15) |

with **A**_outB_ = **T**_1_**A**_outU_ and **A**_B_ = **T**_2_**A**_U_. Considering some ideal situations where **A**_U_ and **A**_B_ (**A**_outB_ and **A**_outU_) are very different or similar with **T**_1_ ≈ α_1_**I** (**T**_2_ ≈ α_2_**I**),

|  | $\frac{1}{\tau_{j}}\approx\left( 1-p_{o} \right)\frac{1}{-\frac{\boldsymbol{u}\boldsymbol{A}_{\boldsymbol{o}\mathbf{utU}}\boldsymbol{A}_{\mathbf{U}}^{-\mathbf{2}}\boldsymbol{Q}}{\boldsymbol{u}\boldsymbol{A}_{\mathbf{outU}}\boldsymbol{A}_{\mathbf{U}}^{-\mathbf{1}}\boldsymbol{Q}}}+p_{0}\frac{1}{-\frac{\boldsymbol{u}\boldsymbol{A}_{\boldsymbol{o}\mathbf{utB}}\boldsymbol{A}_{\mathbf{B}}^{-\mathbf{2}}\boldsymbol{Q}}{\boldsymbol{u}\boldsymbol{A}_{\mathbf{outB}}\boldsymbol{A}_{\mathbf{B}}^{-\mathbf{1}}\boldsymbol{Q}}}=\left( 1-p_{o} \right)\frac{1}{\tau_{iU}}+p_{o}\frac{1}{\tau_{iB}}$. | (D16) |
| --- | --- | --- |

We consider the case of $p_{o}=\frac{\left[ R \right]^{n_{H}}}{K_{H}^{n_{H}}+\left[ R \right]^{n_{H}}}$. In the ideal situation, the bursting variables take the following forms:

|  | $\frac{1}{\tau_{P}}=\frac{\left[ R \right]^{n_{H}}}{K_{H}^{n_{H}}+\left[ R \right]^{n_{H}}}\frac{1}{\tau_{\mathrm{PB}}}+\frac{K_{h}^{n_{H}}}{K_{H}^{n_{H}}+\left[ R \right]^{n_{H}}}\frac{1}{\tau_{\mathrm{PU}}}$, | (D17) |
| --- | --- | --- |
|  | $\frac{1}{\tau_{S1}}=\frac{\left[ R \right]^{n_{H}}}{K_{H}^{n_{H}}+\left[ R \right]^{n_{H}}}\frac{1}{\tau_{S1B}}+\frac{K_{h}^{n_{H}}}{K_{H}^{n_{H}}+\left[ R \right]^{n_{H}}}\frac{1}{\tau_{S1U}}$, | (D18) |
|  | $\frac{1}{\tau_{S2}}=\frac{\left[ R \right]^{n_{H}}}{K_{H}^{n_{H}}+\left[ R \right]^{n_{H}}}\frac{1}{\tau_{S2B}}+\frac{K_{h}^{n_{H}}}{K_{H}^{n_{H}}+\left[ R \right]^{n_{H}}}\frac{1}{\tau_{S2U}}$, | (D19) |
|  | $\frac{1}{\tau_{S3}}=\frac{\left[ R \right]^{n_{H}}}{K_{H}^{n_{H}}+\left[ R \right]^{n_{H}}}\frac{1}{\tau_{S3B}}+\frac{K_{h}^{n_{H}}}{K_{H}^{n_{H}}+\left[ R \right]^{n_{H}}}\frac{1}{\tau_{S3U}}$. | (D20) |

When regulatory factors affect *p*_1_ by changing $J_{1}^{+}$ and affect *p*_2_ by changing $J_{2}^{-}$,

|  | $\frac{1}{1-p_{1}}=\frac{\left[ R \right]^{n_{H}}}{K_{H}^{n_{H}}+\left[ R \right]^{n_{H}}}\frac{1}{1-p_{1B}}+\frac{K_{h}^{n_{H}}}{K_{H}^{n_{H}}+\left[ R \right]^{n_{H}}}\frac{1}{1-p_{1U}}$, | (D21) |
| --- | --- | --- |
|  | $\frac{1}{1-p_{2}}=\frac{\left[ R \right]^{n_{H}}}{K_{H}^{n_{H}}+\left[ R \right]^{n_{H}}}\frac{1}{1-p_{2B}}+\frac{K_{h}^{n_{H}}}{K_{H}^{n_{H}}+\left[ R \right]^{n_{H}}}\frac{1}{1-p_{2U}}$. | (D22) |

When regulatory factors affect *p*_1_ by changing $J_{1}^{-}$ and affect *p*_2_ by changing $J_{2}^{+}$,

|  | $\frac{1}{p_{1}}=\frac{\left[ R \right]^{n_{H}}}{K_{H}^{n_{H}}+\left[ R \right]^{n_{H}}}\frac{1}{p_{1B}}+\frac{K_{h}^{n_{H}}}{K_{H}^{n_{H}}+\left[ R \right]^{n_{H}}}\frac{1}{p_{1U}}$, | (D23) |
| --- | --- | --- |
|  | $\frac{1}{p_{2}}=\frac{\left[ R \right]^{n_{H}}}{K_{H}^{n_{H}}+\left[ R \right]^{n_{H}}}\frac{1}{p_{2B}}+\frac{K_{h}^{n_{H}}}{K_{H}^{n_{H}}+\left[ R \right]^{n_{H}}}\frac{1}{p_{2U}}$. | (D24) |

*τ*_PB_, *τ*_S1B_, *τ*_S2B_, *τ*_S3B_, *p*_1B_ and *p*_2B_ correspond to the case with bound regulators, while *τ*_PU_, *τ*_S1U_, *τ*_S2U_, *τ*_S3U_, *p*_1U_ and *p*_2U_ without bound regulators. According to Eqs. (D18-20, 23), *τ*_S_ can be written as

|  | $\frac{a}{\tau_{S}-\tau_{0}}=\frac{p_{o}}{x_{B}}+\frac{\left( 1-p_{o} \right)}{x_{U}}$, | (D25) |
| --- | --- | --- |

where *x* can be *τ*_S1_, *τ*_S2_, *τ*_S3_ or *p*_2_/(1-*p*_2_). *τ*_0_ and *a* are coefficients, which can be derived from *τ*_S_ = *τ*_S1_+*τ*_S2_*+τ*_S3_ *p*_2_/(1-*p*_2_).

|  | $\frac{1}{\tau_{S}}=\frac{\left[ R \right]^{n_{H}}}{K_{h}^{n_{H}}+\left[ R \right]^{n_{H}}}\frac{1}{\tau_{\mathrm{SB}}}+\frac{K_{h}^{n_{H}}}{K_{h}^{n_{H}}+\left[ R \right]^{n_{H}}}\frac{1}{\tau_{\mathrm{SU}}}$, | (D26) |
| --- | --- | --- |

with $K_{h}=\left( \frac{a+\frac{\tau_{0}}{x_{U}}}{a+\frac{\tau_{0}}{x_{B}}} \right)^{\frac{1}{n_{H}}}K_{H}$.

**Parameter inference**

With methods such as the maximum likelihood method, higher-order moments or distribution functions are required to infer the bursting variables (*τ, p*) *=* (*τ*_P_, *τ*_S1_, *τ*_S2,_ *τ*_S3_, *p*_1_, *p*_2_) at a given regulator concentration [*R*]. That is, only the mean and variance of mRNA copy numbers are insufficient to infer (*τ, p*). Considering that (*τ, p*) are functions of [*R*], we can directly use minimum distance (MD) method to acquire the estimates of (*τ*_PU_, *τ*_PB,_ *τ*_SU_, *τ*_SB_, *p*_1U_, *p*_1B_) with experimental data sets (*m_i_*, *F_i_*). Then, we can get (*τ, p*) through the functions.

For any set of parameters, param = (*τ*_PU_, *τ*_PB,_ *τ*_SU_, *τ*_SB_, *p*_1U_, *p*_1B_), we obtain the expressions for <*m*> and *F* according to Eqs. (2, 4, F17-24) as well as the <*m*>-*F* curve function *L*(<*m>*, *F*) = 0. The Euclidean distance from a datapoint (*m_i_*, *F_i_*) to the curve *L* can be expressed as

| $d_{i}=\min_{L\left( \left\langle m \right\rangle_{L}, F_{L} \right)=0} \sqrt{\left( m_{i}-\left\langle m \right\rangle_{L} \right)^{2}+\left( F_{i}-F_{L} \right)^{2}}$. | (D27) |
| --- | --- |

The total distance is $D=\sum_{i}^{N} d_{i}$ (*N* is the number of datapoints). The estimated parameter values (param_est_) satisfy the following condition:

| $D\left( \mathrm{param}_{\mathrm{est}} \right)=\min_{\mathrm{param}} D$. | (D28) |
| --- | --- |

The stochastic gradient descent (SGD) method was used to find the minimum value of *D*. If *N* > 6 (i.e., the number of parameters), then this estimation is unique; generally, this condition is easily satisfied. With (param_est_), the regulator occupancy rate can be deduced from the value of <*m>*, and we further get the values of (*τ, p*) at a specific <*m>.* If *N <* 6*,* a scheme called approximate Bayesian computation [4] may be used to infer parameter values.

The MD method is widely applicable to various data sets, robust to outliers and extreme values, and easy to implement. Compared with the nonlinear least squares method, the MD method performs better in certain extreme cases (e.g., when *F* rapidly decreases at large *m*).

**Section 5: Comparison between the single- and multi-binding sites cases**

If$\left[ R^{'} \right]$ and$K^{'}$are separately substituted for $\left[ R \right]^{n_{H}}$and $K_{d}^{n_{H}}$ in the preceding section, some expressions are identical in form to those in the case of *n*_H_ = 1, corresponding to the single-binding site case. For eight basic regulatory modes, $\Omega_{X}$/*K*_d_>1(<1) is always maintained in both cases, and *S*_m_ remains the same. The relative relationships between *Ω*_f_, *Ω*_υ_ and *Ω*_b_ remain unchanged. The <*m*>-*F* curves under all conditions remain the same. Therefore, the conclusions drawn in the text hold true.

Table S1 presents the expressions for quantities for both the single- and multi-binding sites cases.

| Table S1: Quantities in both cases | | |
| --- | --- | --- |
| Variable | With one binding site | With multiple binding sites |
| occupancy rate | $\frac{\left[ R \right]}{\left[ R \right]+K_{d}}$ | $\frac{\left[ R \right]^{n_{H}}}{\left[ R \right]^{n_{H}}+K_{d}^{n_{H}}}$ |
| *τ*_P_ ([*R*]) | $\frac{1}{\tau_{P}}=\frac{\left[ R \right]}{\left[ R \right]+K_{d}}\frac{1}{\tau_{\mathrm{PB}}}+\frac{K_{d}}{\left[ R \right]+K_{d}}\frac{1}{\tau_{\mathrm{PU}}}$ | $\frac{1}{\tau_{P}}=\frac{\left[ R \right]^{n_{H}}}{K_{H}^{n_{H}}+\left[ R \right]^{n_{H}}}\frac{1}{\tau_{\mathrm{PB}}}+\frac{K_{H}^{n_{H}}}{K_{H}^{n_{H}}+\left[ R \right]^{n_{H}}}\frac{1}{\tau_{\mathrm{PU}}}$ |
| *τ*_S1_([*R*]) | $\frac{1}{\tau_{S1}}=\frac{\left[ R \right]}{\left[ R \right]+K_{d}}\frac{1}{\tau_{S1B}}+\frac{K_{d}}{\left[ R \right]+K_{d}}\frac{1}{\tau_{S1U}}$ | $\frac{1}{\tau_{S1}}=\frac{\left[ R \right]^{n_{H}}}{K_{H}^{n_{H}}+\left[ R \right]^{n_{H}}}\frac{1}{\tau_{S1B}}+\frac{K_{H}^{n_{H}}}{K_{H}^{n_{H}}+\left[ R \right]^{n_{H}}}\frac{1}{\tau_{S1U}}$ |
| *τ*_S2_ ([*R*]) | $\frac{1}{\tau_{S2}}=\frac{\left[ R \right]}{\left[ R \right]+K_{d}}\frac{1}{\tau_{S2B}} +\frac{K_{d}}{\left[ R \right]+K_{d}}\frac{1}{\tau_{S2U}}$ | $\frac{1}{\tau_{S2}}=\frac{\left[ R \right]^{n_{H}}}{K_{H}^{n_{H}}+\left[ R \right]^{n_{H}}}\frac{1}{\tau_{S2B}} +\frac{K_{H}^{n_{H}}}{K_{H}^{n_{H}}+\left[ R \right]^{n_{H}}}\frac{1}{\tau_{S2U}}$ |
| *τ*_S3_ ([*R*]) | $\frac{1}{\tau_{S3}}=\frac{\left[ R \right]}{\left[ R \right]+K_{d}}\frac{1}{\tau_{S3B}} +\frac{K_{d}}{\left[ R \right]+K_{d}}\frac{1}{\tau_{S3U}}$ | $\frac{1}{\tau_{S3}}=\frac{\left[ R \right]^{n_{H}}}{K_{H}^{n_{H}}+\left[ R \right]^{n_{H}}}\frac{1}{\tau_{S3B}} +\frac{K_{H}^{n_{H}}}{K_{H}^{n_{H}}+\left[ R \right]^{n_{H}}}\frac{1}{\tau_{S3U}}$ |
| *p*_1_ ([*R*]) via *J*_1_^+^ | $\frac{1}{1-p_{1}}=\frac{\left[ R \right]}{\left[ R \right]+K_{d}}\frac{1}{1-p_{1B}} +\frac{K_{d}}{\left[ R \right]+K_{d}}\frac{1}{1-p_{1U}}$ | $\frac{1}{1-p_{1}}=\frac{\left[ R \right]^{n_{H}}}{K_{H}^{n_{H}}+\left[ R \right]^{n_{H}}}\frac{1}{1-p_{1B}} +\frac{K_{H}^{n_{H}}}{K_{H}^{n_{H}}+\left[ R \right]^{n_{H}}}\frac{1}{1-p_{1U}}$ |
| *p*_1_ ([*R*]) via *J*_1_^-^ | $\frac{1}{p_{1}}=\frac{\left[ R \right]}{\left[ R \right]+K_{d}}\frac{1}{p_{1B}}+\frac{K_{d}}{\left[ R \right]+K_{d}}\frac{1}{p_{1U}}$ | $\frac{1}{p_{1}}=\frac{\left[ R \right]^{n_{H}}}{K_{H}^{n_{H}}+\left[ R \right]^{n_{H}}}\frac{1}{p_{1B}}+\frac{K_{H}^{n_{H}}}{K_{H}^{n_{H}}+\left[ R \right]^{n_{H}}}\frac{1}{p_{1U}}$ |
| *p*_2_ ([*R*]) via *J*_2_^+^ | $\frac{1}{p_{2}}=\frac{\left[ R \right]}{\left[ R \right]+K_{d}}\frac{1}{p_{2B}}+\frac{K_{d}}{\left[ R \right]+K_{d}}\frac{1}{p_{2U}}$ | $\frac{1}{p_{2}}=\frac{\left[ R \right]^{n_{H}}}{K_{H}^{n_{H}}+\left[ R \right]^{n_{H}}}\frac{1}{p_{2B}}+\frac{K_{H}^{n_{H}}}{K_{H}^{n_{H}}+\left[ R \right]^{n_{H}}}\frac{1}{p_{2U}}$ |
| *p*_2_ ([*R*]) via *J*_2_^-^ | $\frac{1}{1-p_{2}}=\frac{\left[ R \right]}{\left[ R \right]+K_{d}}\frac{1}{1-p_{2B}} +\frac{K_{d}}{\left[ R \right]+K_{d}}\frac{1}{1-p_{2U}}$ | $\frac{1}{1-p_{2}}=\frac{\left[ R \right]^{n_{H}}}{K_{H}^{n_{H}}+\left[ R \right]^{n_{H}}}\frac{1}{1-p_{2B}} +\frac{K_{H}^{n_{H}}}{K_{H}^{n_{H}}+\left[ R \right]^{n_{H}}}\frac{1}{1-p_{2U}}$ |
| *υ* | $\upsilon_{0}\frac{\beta_{X}^{n_{0}}+\left[ R \right]^{n_{0}}}{\Omega_{X}^{n_{0}}+\left[ R \right]^{n_{0}}}$ | $\upsilon_{0}\frac{\beta_{X}^{n_{0}n_{H}}+\left[ R \right]^{n_{0}n_{H}}}{\Omega_{X}^{n_{0}n_{H}}+\left[ R \right]^{n_{0}n_{H}}}$ |
| *n* | *n*_0_ | *n*_0_*n*_H_ |
| *υ*_0_ | Unchanged | |
| $\beta_{\mathrm{EE}}$ | $\varepsilon_{\mathrm{EE}}K_{d}$ | $\varepsilon_{\mathrm{EE}}^{\frac{1}{n_{H}}}K_{d}$ |
| $\beta_{\mathrm{EA}}$ | $\varepsilon_{\mathrm{EA}}K_{d}$ | $\varepsilon_{\mathrm{EA}}^{\frac{1}{n_{H}}}K_{d}$ |
| $\beta_{\mathrm{FI}}$ | $\varepsilon_{\mathrm{FI}}K_{d}$ | $\varepsilon_{\mathrm{FI}}^{\frac{1}{n_{H}}}K_{d}$ |
| $\beta_{\mathrm{CC}}$ | $\varepsilon_{\mathrm{CC}}K_{d}$ | $\varepsilon_{\mathrm{CC}}^{\frac{1}{n_{H}}}K_{d}$ |
| $\beta_{\mathrm{IE}}$ | $\frac{1-\varepsilon_{\mathrm{IE}}p_{1U}}{\varepsilon_{\mathrm{IE}}\left( 1-p_{1U} \right)}K_{d}$ | $\left[ \frac{1-\varepsilon_{\mathrm{IE}}p_{1U}}{\varepsilon_{\mathrm{IE}}\left( 1-p_{1U} \right)} \right]^{\frac{1}{n_{H}}}K_{d}$ |
| $\beta_{\mathrm{IA}}$ | $K_{d}$ | $K_{d}$ |
| $\beta_{\mathrm{FC}}$ | $K_{d}$ | $K_{d}$ |
| $\beta_{\mathrm{FA}}$ | $\frac{\varepsilon_{\mathrm{FA}}\left( 1-p_{2U} \right)}{1-\varepsilon_{\mathrm{FA}}p_{2U}}K_{d}$ | $\left[ \frac{\varepsilon_{\mathrm{FA}}\left( 1-p_{2U} \right)}{1-\varepsilon_{\mathrm{FA}}p_{2U}} \right]^{\frac{1}{n_{H}}}K_{d}$ |
| $\Omega_{\mathrm{EE}}$ | $\frac{\tau_{\mathrm{AU}}+\tau_{I}}{\tau_{\mathrm{AU}}+\frac{\tau_{I}}{\varepsilon_{\mathrm{EE}}}}K_{d}<K_{d}$ | $\left( \frac{\tau_{\mathrm{AU}}+\tau_{I}}{\tau_{\mathrm{AU}}+\frac{\tau_{I}}{\varepsilon_{\mathrm{EE}}}} \right)^{\frac{1}{n_{H}}}K_{d}<K_{d}$ |
| $\Omega_{\mathrm{EA}}$ | $\frac{\varepsilon_{\mathrm{EA}}\tau_{I1U}+\varepsilon_{\mathrm{EA}}\left( \tau_{A}+\tau_{I2} \right)}{\varepsilon_{\mathrm{EA}}\tau_{I1U}+\left( \tau_{A}+\tau_{I2} \right)}K_{d}<K_{d}$ | $\left[ \frac{\varepsilon_{\mathrm{EA}}\tau_{I1U}+\varepsilon_{\mathrm{EA}}\left( \tau_{A}+\tau_{I2} \right)}{\varepsilon_{\mathrm{EA}}\tau_{I1U}+\left( \tau_{A}+\tau_{I2} \right)} \right]^{\frac{1}{n_{H}}}K_{d}<K_{d}$ |
| $\Omega_{\mathrm{FI}}$ | $\frac{\varepsilon_{\mathrm{FI}}\tau_{I1U}+\varepsilon_{\mathrm{FI}}\left( \tau_{A}+\tau_{I2} \right)}{\varepsilon_{\mathrm{FI}}\tau_{I1U}+\left( \tau_{A}+\tau_{I2} \right)}K_{d}<K_{d}$ | $\left[ \frac{\varepsilon_{\mathrm{FI}}\tau_{I1U}+\varepsilon_{\mathrm{FI}}\left( \tau_{A}+\tau_{I2} \right)}{\varepsilon_{\mathrm{FI}}\tau_{I1U}+\left( \tau_{A}+\tau_{I2} \right)} \right]^{\frac{1}{n_{H}}}K_{d}<K_{d}$ |
| $\Omega_{\mathrm{CC}}$ | $\frac{\varepsilon_{\mathrm{CC}}\left( \tau_{A}+\tau_{I1} \right)+\varepsilon_{\mathrm{CC}}\tau_{I2U}}{\tau_{A}+\tau_{I1}+\varepsilon_{\mathrm{CC}}\tau_{I2U}}K_{d}<K_{d}$ | $\left[ \frac{\varepsilon_{\mathrm{CC}}\left( \tau_{A}+\tau_{I1} \right)+\varepsilon_{\mathrm{CC}}\tau_{I2U}}{\tau_{A}+\tau_{I1}+\varepsilon_{\mathrm{CC}}\tau_{I2U}} \right]^{\frac{1}{n_{H}}}K_{d}<K_{d}$ |
| $\Omega_{\mathrm{IE}}$ | $\frac{\tau_{\mathrm{AU}}+\tau_{\mathrm{IU}}}{\frac{\varepsilon_{\mathrm{IE}}\left( 1-p_{1U} \right)}{1-\varepsilon_{\mathrm{IE}}p_{1U}}\tau_{\mathrm{AU}}+\tau_{\mathrm{IU}}}K_{d}<K_{d}$ | $\left[ \frac{\tau_{\mathrm{AU}}+\tau_{\mathrm{IU}}}{\frac{\varepsilon_{\mathrm{IE}}\left( 1-p_{1U} \right)}{1-\varepsilon_{\mathrm{IE}}p_{1U}}\tau_{\mathrm{AU}}+\tau_{\mathrm{IU}}} \right]^{\frac{1}{n_{H}}}K_{d}<K_{d}$ |
| $\Omega_{\mathrm{IA}}$ | $\frac{\tau_{\mathrm{AU}}+\tau_{\mathrm{IU}}}{\tau_{\mathrm{AU}}+\frac{1-\varepsilon_{\mathrm{IA}}p_{1U}}{\varepsilon_{\mathrm{IA}}\left( 1-p_{1U} \right)}\tau_{\mathrm{IU}}}K_{d}>K_{d}$ | $\left[ \frac{\tau_{\mathrm{AU}}+\tau_{\mathrm{IU}}}{\tau_{\mathrm{AU}}+\frac{1-\varepsilon_{\mathrm{IA}}p_{1U}}{\varepsilon_{\mathrm{IA}}\left( 1-p_{1U} \right)}\tau_{\mathrm{IU}}} \right]^{\frac{1}{n_{H}}}K_{d}>K_{d}$ |
| $\Omega_{\mathrm{FC}}$ | $\frac{\tau_{A}+\tau_{I1}+\tau_{I2U}}{\tau_{A}+\tau_{I1}+\frac{\varepsilon_{\mathrm{FC}}\left( 1-p_{2U} \right)}{1-\varepsilon_{\mathrm{FC}}p_{2U}}\tau_{I2U}}K_{d}>K_{d}$ | $\left[ \frac{\tau_{A}+\tau_{I1}+\tau_{I2U}}{\tau_{A}+\tau_{I1}+\frac{\varepsilon_{\mathrm{FC}}\left( 1-p_{2U} \right)}{1-\varepsilon_{\mathrm{FC}}p_{2U}}\tau_{I2U}} \right]^{\frac{1}{n_{H}}}K_{d}>K_{d}$ |
| $\Omega_{\mathrm{FA}}$ | $\frac{\tau_{A}+\tau_{I1}+\tau_{I2U}}{\left( \tau_{A}+\tau_{I1} \right)\frac{1-\varepsilon_{\mathrm{FA}}p_{2U}}{\varepsilon_{\mathrm{FA}}\left( 1-p_{2U} \right)}+\tau_{I2U}}K_{d}<K_{d}$ | $\left[ \frac{\tau_{A}+\tau_{I1}+\tau_{I2U}}{\left( \tau_{A}+\tau_{I1} \right)\frac{1-\varepsilon_{\mathrm{FA}}p_{2U}}{\varepsilon_{\mathrm{FA}}\left( 1-p_{2U} \right)}+\tau_{I2U}} \right]^{\frac{1}{n_{H}}}K_{d}<K_{d}$ |
| *b* ([*R*]) | $b_{B}\frac{\left[ R \right]+K_{d}\frac{b_{U}}{b_{B}}}{\left[ R \right]+K_{d}}$ | $b_{B}\frac{\left[ R \right]^{n_{H}}+K_{d}^{n_{H}}\frac{b_{U}}{b_{B}}}{\left[ R \right]^{n_{H}}+K_{d}^{n_{H}}}$ |
| *Ω*_b_ | *K*_d_ | *K*_d_ |
| *f* ([*R*]) | $\frac{\upsilon_{0}}{b_{B}}\frac{\beta_{X}^{n_{0}}+\left[ R \right]^{n_{0}}}{\Omega_{X}^{n_{0}}+\left[ R \right]^{n_{0}}}\frac{\left[ R \right]+K_{d}}{\left[ R \right]+K_{d}\frac{b_{U}}{b_{B}}}$ | $\frac{\upsilon_{0}}{b_{B}}\frac{\beta_{X}^{n_{0}n_{H}}+\left[ R \right]^{n_{0}n_{H}}}{\Omega_{X}^{n_{0}n_{H}}+\left[ R \right]^{n_{0}n_{H}}}\frac{\left[ R \right]^{n_{H}}+K_{d}^{n_{H}}}{\left[ R \right]^{n_{H}}+K_{d}^{n_{H}}\frac{b_{U}}{b_{B}}}$ |
| *S* ([*R*]) | $\frac{\upsilon_{max1}}{\upsilon_{max0}}\frac{\beta_{1}^{n_{0}}+\left[ R \right]^{n_{0}}}{\beta_{0}^{n_{0}}+\left[ R \right]^{n_{0}}}\frac{\Omega_{0}^{n_{0}}+\left[ R \right]^{n_{0}}}{\Omega_{1}^{n_{0}}+\left[ R \right]^{n_{0}}}$ | $\frac{\upsilon_{max1}}{\upsilon_{max0}}\frac{\beta_{1}^{n_{0}n_{H}}+\left[ R \right]^{n_{0}n_{H}}}{\beta_{0}^{n_{0}n_{H}}+\left[ R \right]^{n_{0}n_{H}}}\frac{\Omega_{0}^{n_{0}n_{H}}+\left[ R \right]^{n_{0}n_{H}}}{\Omega_{1}^{n_{0}n_{H}}+\left[ R \right]^{n_{0}n_{H}}}$ |
| *S*_m_ | Unchanged | |
| *S*_m,EE_ (*Ω*_EE_/*K*_d_) | $1+\frac{\tau_{\mathrm{AU}}}{\tau_{\mathrm{IU}}}\left( 1-\frac{\Omega_{\mathrm{EE}}}{K_{d}} \right)$ | $1+\frac{\tau_{\mathrm{AU}}}{\tau_{\mathrm{IU}}}\left[ 1-\left( \frac{\Omega_{\mathrm{EE}}}{K_{d}} \right)^{n_{H}} \right]$ |
| *S*_m,EA_ (*Ω*_EA_/*K*_d_) | $1+\frac{\frac{1}{p_{1}}\tau_{S1U}}{\tau_{\mathrm{AU}}+\frac{1}{p_{1}}\tau_{S2U}+\tau_{I2U}}\left( 1-\frac{\Omega_{\mathrm{EA}}}{K_{d}} \right)$ | $1+\frac{\frac{1}{p_{1}}\tau_{S1U}}{\tau_{\mathrm{AU}}+\frac{1}{p_{1}}\tau_{S2U}+\tau_{I2U}}\left[ 1-\left( \frac{\Omega_{\mathrm{EA}}}{K_{d}} \right)^{n_{H}} \right]$ |
| *S*_m,FI_ (*Ω*_FI_/*K*_d_) | $1+\frac{\frac{1}{p_{1}}\tau_{S2U}}{\tau_{\mathrm{AU}}+\frac{1}{p_{1}}\tau_{S1U}+\tau_{I2U}}\left( 1-\frac{\Omega_{\mathrm{FI}}}{K_{d}} \right)$ | $1+\frac{\frac{1}{p_{1}}\tau_{S2U}}{\tau_{\mathrm{AU}}+\frac{1}{p_{1}}\tau_{S1U}+\tau_{I2U}}\left[ 1-\left( \frac{\Omega_{\mathrm{FI}}}{K_{d}} \right)^{n_{H}} \right]$ |
| *S*_m,CC_ (*Ω*_CC_/*K*_d_) | $1+\frac{\tau_{I2U}}{\tau_{\mathrm{AU}}+\tau_{I1U}}\left( 1-\frac{\Omega_{\mathrm{CC}}}{K_{d}} \right)$ | $1+\frac{\tau_{I2U}}{\tau_{\mathrm{AU}}+\tau_{I1U}}\left[ 1-\left( \frac{\Omega_{\mathrm{CC}}}{K_{d}} \right)^{n_{H}} \right]$ |
| *S*_m,IE_ (*Ω*_IE_/*K*_d_) | $1+\frac{\tau_{\mathrm{IU}}}{\tau_{\mathrm{AU}}}\left( 1-\frac{\Omega_{\mathrm{IE}}}{K_{d}} \right)$ | $1+\frac{\tau_{\mathrm{IU}}}{\tau_{\mathrm{AU}}}\left[ 1-\left( \frac{\Omega_{\mathrm{IE}}}{K_{d}} \right)^{n_{H}} \right]$ |
| *S*_m,IA_ (*Ω*_IA_/*K*_d_) | $\frac{\Omega_{\mathrm{IA}}}{K_{d}}$ | $\left( \frac{\Omega_{\mathrm{IA}}}{K_{d}} \right)^{n_{H}}$ |
| *S*_m,FC_ (*Ω*_FC_/*K*_d_) | $\frac{\Omega_{\mathrm{FC}}}{K_{d}}$ | $\left( \frac{\Omega_{\mathrm{FC}}}{K_{d}} \right)^{n_{H}}$ |
| *S*_m,FA_ (*Ω*_FA_/*K*_d_) | $1+\frac{\tau_{I2U}}{\tau_{\mathrm{AU}}+\tau_{I1U}}\left( 1-\frac{\Omega_{\mathrm{FA}}}{K_{d}} \right)$ | $1+\frac{\tau_{I2U}}{\tau_{\mathrm{AU}}+\tau_{I1U}}\left[ 1-\left( \frac{\Omega_{\mathrm{FA}}}{K_{d}} \right)^{n_{H}} \right]$ |
| *<m>* | $\frac{\tau_{m}}{\tau_{P}+\frac{1-p_{1}}{p_{1}}\left( \tau_{S1}+\tau_{S2}+\frac{p_{2}}{1-p_{2}}\tau_{S3} \right)}$ | |
| *F* | $1+\left\langle m \right\rangle\frac{\left( \frac{\tau_{I}}{\tau_{m}}+\frac{\tau_{A}}{\tau_{m}} \right)\frac{\tau_{S}}{\tau_{m}}-\frac{\tau_{A}}{\tau_{m}}\frac{\tau_{I}}{\tau_{m}}}{\frac{\tau_{I}}{\tau_{m}}+\frac{\tau_{A}}{\tau_{m}}+\frac{\tau_{A}}{\tau_{m}}\frac{\tau_{I}}{\tau_{m}}}$ | |
| *F* ~ *<m>* | Unchanged | |

**Section 6: Gene regulatory function**

We use the average transcription rate in steady state ($\upsilon$) to probe the sensitivity of transcription to changes in$[R]$. Given

$\left\langle m \right\rangle=\upsilon\tau_{m}$, $\left\langle m \right\rangle=\frac{\tau_{m}}{\tau_{P}+\frac{1-p_{1}}{p_{1}}\left( \tau_{S1}+\tau_{S2}+\frac{p_{2}}{1-p_{2}}\tau_{S3} \right)}$, (F1)

*υ* can take the following form:

$\upsilon=\frac{1}{\tau_{P}+\frac{1-p_{1}}{p_{1}}\left( \tau_{S1}+\tau_{S2}+\frac{p_{2}}{1-p_{2}}\tau_{S3} \right)}$. (F2)

Because *τ*_P_, *τ*_S1_, *τ*_S2_, *τ*_S3_, *p*_1_ and *p*_2_ are functions of $\left[ R \right]$, *υ* is also a function of $\left[ R \right]$:

$\upsilon\approx\upsilon_{\max}\frac{\beta^{n}+\left[ R \right]^{n}}{\Omega^{n}+\left[ R \right]^{n}}$. (F3)

*Ω* is the operating point where *υ* is the mid-point between *υ*(0) and *υ*(∞). *β* determines the basal transcription rate, and *n* is a fitting number. The curve of *υ* versus [*R*] takes different forms under different regulation modes. *ε*_X_ (X=EE, EA, FI, CC, IE, IA, FC, FA) is the ratio of the corresponding parameter with regulators bound to that without regulators bound.

In MEE, *τ*_PB_ = *ε*_EE_*τ*_PU_ with *ε*_EE_ < 1 and the other parameters remain unchanged. *υ*_EE_ takes the following form:

$\upsilon_{\mathrm{EE}}=\frac{1}{1-p_{1}}\frac{1}{\varepsilon_{\mathrm{EE}}\tau_{\mathrm{AU}}+\tau_{I}}\frac{\varepsilon_{\mathrm{EEE}}K_{d}+[R]}{\frac{\varepsilon_{\mathrm{EE}}\tau_{\mathrm{AU}}+\varepsilon_{\mathrm{EE}}\tau_{I}}{\varepsilon_{\mathrm{EE}}\tau_{\mathrm{AU}}+\tau_{I}}K_{d}+[R]},$ (F4)

as regulators affect *τ*_P_ with *τ*_AU_ =$\frac{\tau_{\mathrm{PU}}}{1-p_{1}}$ and $\tau_{I}=\frac{1}{p_{1}}\left( \tau_{S1}+\tau_{S2}+\frac{p_{2}}{1-p_{2}}\tau_{S3} \right)$. The operating point is

$\Omega_{\mathrm{EE}}=\frac{\tau_{\mathrm{AU}}+\tau_{I}}{\tau_{\mathrm{AU}}+\tau_{I}/\varepsilon_{\mathrm{EE}}}K_{d}<K_{d}.$ (F5)

In MIE and MIA, *p*_1B_ = *ε*_i_*p*_1U_ (i = IE or IA) with *ε*_i_ > 1 and the other parameters remain fixed. *υ*_IE_ takes the following form:

$\upsilon_{\mathrm{IE}}=\frac{1}{1-p_{1U}}\frac{1}{\tau_{\mathrm{AU}}+\frac{1-\varepsilon_{\mathrm{IE}}p_{1U}}{\varepsilon_{\mathrm{IE}}\left( 1-p_{1U} \right)}\tau_{\mathrm{IU}}}\frac{K_{d}\frac{1-\varepsilon_{\mathrm{IE}}p_{1U}}{\varepsilon_{\mathrm{IE}}\left( 1-p_{1U} \right)}+\left[ R \right]}{\frac{\tau_{\mathrm{AU}}+\tau_{\mathrm{IU}}}{\frac{\varepsilon_{\mathrm{IE}}\left( 1-p_{1U} \right)}{1-\varepsilon_{\mathrm{IE}}p_{1U}}\tau_{\mathrm{AU}}+\tau_{\mathrm{IU}}}K_{d}+\left[ R \right]}$, (F6)

as regulators affect *p*_1_ via *J*_1_^+^. The operating point is

$\Omega_{\mathrm{IE}}=\frac{\tau_{\mathrm{AU}}+\tau_{\mathrm{IU}}}{\frac{\varepsilon_{\mathrm{IE}}\left( 1-p_{1U} \right)}{1-\varepsilon_{\mathrm{IE}}p_{1U}}\tau_{\mathrm{AU}}+\tau_{\mathrm{IU}}}K_{d}<K_{d}$. (F7)

When regulatory factors affect *p*_1_ via *J*_1_^-^, *υ*_IA_ and the operating point *Ω*_IA_ take the following forms:

$\upsilon_{\mathrm{IA}}=\frac{1}{1-p_{1U}}\frac{1}{\tau_{\mathrm{AU}}+\frac{1-\varepsilon_{\mathrm{IA}}p_{1U}}{\varepsilon_{\mathrm{IA}}\left( 1-p_{1U} \right)}\tau_{\mathrm{IU}}}\frac{K_{d}+\left[ R \right]}{\frac{\tau_{\mathrm{AU}}+\tau_{\mathrm{IU}}}{\tau_{\mathrm{AU}}+\frac{1-\varepsilon_{\mathrm{IA}}p_{1U}}{\varepsilon_{\mathrm{IA}}\left( 1-p_{1U} \right)}\tau_{\mathrm{IU}}}K_{d}+\left[ R \right]}$ (F8)

$\Omega_{\mathrm{IA}}=\frac{\tau_{\mathrm{AU}}+\tau_{\mathrm{IU}}}{\tau_{\mathrm{AU}}+\frac{1-\varepsilon_{\mathrm{IA}}p_{1U}}{\varepsilon_{\mathrm{IA}}\left( 1-p_{1U} \right)}\tau_{\mathrm{IU}}}K_{d}>K_{d}$. (F9)

In MEA, *τ*_S1B_ = *ε*_EA_*τ*_S1U_ with *ε*_EA_ < 1 and the other parameters remain constant. *υ*_EA_ takes the following form:

$\upsilon_{\mathrm{EA}}=\frac{1}{1-p_{1}}\frac{1}{\tau_{A}+\varepsilon_{\mathrm{EA}}\frac{1}{p_{1}}\tau_{S1U}+\left( \frac{1}{p_{1}}\tau_{S2}+\tau_{I2} \right)}\frac{\varepsilon_{\mathrm{EA}}K_{d}+[R]}{\frac{\varepsilon_{\mathrm{EA}}\frac{1}{p_{1}}\tau_{S1U}+\varepsilon_{\mathrm{EA}}\left( \tau_{A}+\frac{1}{p_{1}}\tau_{S2}+\tau_{I2} \right)}{\varepsilon_{\mathrm{EA}}\frac{1}{p_{1}}\tau_{S1U}+\left( \tau_{A}+\frac{1}{p_{1}}\tau_{S2}+\tau_{I2} \right)}K_{d}+[R]}$. (F10)

The operating point is

$\Omega_{\mathrm{EA}}=\frac{\frac{1}{p_{1}}\tau_{S1U}+\left( \tau_{A}+\frac{1}{p_{1}}\tau_{S2}+\tau_{I2} \right)}{\frac{1}{p_{1}}\tau_{S1U}+\left( \tau_{A}+\frac{1}{p_{1}}\tau_{S2}+\tau_{I2} \right)/\varepsilon_{\mathrm{EA}}}K_{d}<K_{d}$. (F11)

In MFI, *τ*_S2B_ = *ε*_FI_*τ*_S2U_ with *ε*_FI_ < 1 and the other parameters remain unchanged. *υ*_FI_ takes the following form:

$\upsilon_{\mathrm{FI}}=\frac{1}{1-p_{1}}\frac{1}{\tau_{A}+\varepsilon_{\mathrm{FI}}\frac{1}{p_{1}}\tau_{S2U}+\left( \frac{1}{p_{1}}\tau_{S1}+\tau_{I2} \right)}\frac{\varepsilon_{\mathrm{FI}}K_{d}+[R]}{\frac{\varepsilon_{\mathrm{FI}}\frac{1}{p_{1}}\tau_{S2U}+\varepsilon_{\mathrm{FI}}\left( \tau_{A}+\frac{1}{p_{1}}\tau_{S1}+\tau_{I2} \right)}{\varepsilon_{\mathrm{FI}}\frac{1}{p_{1}}\tau_{S2U}+\left( \tau_{A}+\frac{1}{p_{1}}\tau_{S1}+\tau_{I2} \right)}K_{d}+[R]}.$ (F12)

The operating point is

$\Omega_{\mathrm{FI}}=\frac{\frac{1}{p_{1}}\tau_{S2U}+\left( \tau_{A}+\frac{1}{p_{1}}\tau_{S1}+\tau_{I2} \right)}{\frac{1}{p_{1}}\tau_{S2U}+\left( \tau_{A}+\frac{1}{p_{1}}\tau_{S1}+\tau_{I2} \right)/\varepsilon_{\mathrm{FI}}}K_{d}<K_{d}.$ (F13)

In MFA and MFC, *p*_2B_ = *ε*_i_*p*_2U_ (i = FA or FC) with *ε*_i_ < 1and the other parameters are fixed. *υ*_FC_ takes the following form:

$\upsilon_{\mathrm{FC}}=\frac{1}{1-p_{1}}\frac{1}{\tau_{A}+\tau_{I1}+\frac{\varepsilon_{\mathrm{FC}}\left( 1-p_{2U} \right)}{1-\varepsilon_{\mathrm{FC}}p_{2U}}\tau_{I2U}}\frac{K_{d}+\left[ R \right]}{\frac{\tau_{A}+\tau_{I1}+\tau_{I2U}}{\tau_{A}+\tau_{I1}+\frac{\varepsilon_{\mathrm{FC}}\left( 1-p_{2U} \right)}{1-\varepsilon_{\mathrm{FC}}p_{2U}}\tau_{I2U}}K_{d}+\left[ R \right]}$, (F14)

as regulatory factors affect *p*_2_ via *J*_2_^+^. The operating point is

$\Omega_{\mathrm{FC}}=\frac{\tau_{A}+\tau_{I1}+\tau_{I2U}}{\tau_{A}+\tau_{I1}+\frac{\varepsilon_{\mathrm{FC}}\left( 1-p_{2U} \right)}{1-\varepsilon_{\mathrm{FC}}p_{2U}}\tau_{I2U}}K_{d}>K_{d}$. (F15)

When regulatory factors affect *p*_2_ via *J*_2_^-^, *υ*_FA_ and the operating point *Ω*_FA_ take the following forms:

$\upsilon_{\mathrm{FA}}=\frac{1}{1-p_{1}}\frac{1}{\tau_{A}+\tau_{I1}+\frac{\varepsilon_{\mathrm{FA}}(1-p_{2U})}{1-\varepsilon_{\mathrm{FA}}p_{2U}}\tau_{I2U}}\frac{\frac{\varepsilon_{\mathrm{FA}}\left( 1-p_{2U} \right)}{1-\varepsilon_{\mathrm{FA}}p_{2U}}K_{d}+\left[ R \right]}{\frac{\tau_{A}+\tau_{I1}+\tau_{I2U}}{\left( \tau_{A}+\tau_{I1} \right)\frac{1-\varepsilon_{\mathrm{FA}}p_{2U}}{\varepsilon_{\mathrm{FA}}\left( 1-p_{2U} \right)}+\tau_{I2U}}K_{d}+\left[ R \right]}$ (F16)

$\Omega_{\mathrm{FA}}=\frac{\tau_{A}+\tau_{I1}+\tau_{I2U}}{\left( \tau_{A}+\tau_{I1} \right)\frac{1-\varepsilon_{\mathrm{FA}}p_{2U}}{\varepsilon_{\mathrm{FA}}\left( 1-p_{2U} \right)}+\tau_{I2U}}K_{d}<K_{d}.$ (F17)

In MCC, *τ*_S3B_ = *ε*_CC_*τ*_S3U_ with *ε*_CC_ < 1 and the other parameters remain constant. *υ*_CC_ takes the following form:

$\upsilon_{\mathrm{CC}}=\frac{1}{1-p_{1}}\frac{1}{\tau_{A}+\tau_{I1}+\varepsilon_{\mathrm{CC}}\tau_{I2U}}\frac{\varepsilon_{\mathrm{CC}}K_{d}+[R]}{\frac{\varepsilon_{\mathrm{CC}}\left( \tau_{A}+\tau_{I1} \right)+\varepsilon_{\mathrm{CC}}\tau_{I2U}}{\tau_{A}+\tau_{I1}+\varepsilon_{\mathrm{CC}}\tau_{I2U}}K_{d}+[R]}$. (F18)

The operating point is

$\Omega_{\mathrm{CC}}=\frac{\tau_{A}+\tau_{I1}+\tau_{I2U}}{\left( \tau_{A}+\tau_{I1} \right)/\varepsilon_{\mathrm{CC}}+\tau_{I2U}}K_{d}<K_{d}.$ (F19)

The results above indicate that *Ω*_X_ (X= EE, IE, EA, FI, FA, CC) is less than *K*_d_ when the binding of regulators promotes transcription by facilitating the regulated reactions, whereas *Ω*_X_ (X = IA, FC) is greater than *K*_d_ with regulator binding inhibiting reactions. In the case where *υ* is sensitive to changes in [*R*] (*Ω*_X_ < *K*_d_) with the same basal transcription and fold change, the relationships between *Ω*_X_ take the following forms [Fig. S12]:

$\tau_{A}>\tau_{I1}>\tau_{I2} \mathrm{and}$ $\tau_{A}>\tau_{I1}+\tau_{I2}$: $\Omega_{\mathrm{EE}}>\Omega_{\mathrm{IE}}>\Omega_{\mathrm{EA}},\Omega_{\mathrm{FI}}>\Omega_{\mathrm{FA}},\Omega_{\mathrm{CC}}$

$\tau_{A}>\tau_{I2}>\tau_{I1} \mathrm{and}\tau_{A}>\tau_{I1}+\tau_{I2}$: $\Omega_{\mathrm{EE}}>\Omega_{\mathrm{IE}}>\Omega_{\mathrm{FA}},\Omega_{\mathrm{CC}}>\Omega_{\mathrm{EA}},\Omega_{\mathrm{FI}}$

$\tau_{A}>\tau_{I1}>\tau_{I2} \mathrm{and}$ $\tau_{A}<\tau_{I1}+\tau_{I2}$: $\Omega_{\mathrm{IE}}>\Omega_{\mathrm{EE}}>\Omega_{\mathrm{EA}},\Omega_{\mathrm{FI}}>\Omega_{\mathrm{FA}},\Omega_{\mathrm{CC}}$

$\tau_{A}>\tau_{I2}>\tau_{I1} \mathrm{and}\tau_{A}<\tau_{I1}+\tau_{I2}$: $\Omega_{\mathrm{IE}}>\Omega_{\mathrm{EE}}>\Omega_{\mathrm{FA}},\Omega_{\mathrm{CC}}>\Omega_{\mathrm{EA}},\Omega_{\mathrm{FI}}$

$\tau_{I1}>\tau_{A}>\tau_{I2}$: $\Omega_{\mathrm{IE}}>\Omega_{\mathrm{EA}},\Omega_{\mathrm{FI}}>\Omega_{\mathrm{EE}}>\Omega_{\mathrm{FA}},\Omega_{\mathrm{CC}}$

$\tau_{I2}>\tau_{A}>\tau_{I1}$: $\Omega_{\mathrm{IE}}>\Omega_{\mathrm{FA}},\Omega_{\mathrm{CC}}>\Omega_{\mathrm{EE}}>\Omega_{\mathrm{EA}},\Omega_{\mathrm{FI}}$

$\tau_{I1}>\tau_{I2}>\tau_{A}$: $\Omega_{\mathrm{IE}}>\Omega_{\mathrm{EA}},\Omega_{\mathrm{FI}}>\Omega_{\mathrm{FA}},\Omega_{\mathrm{CC}}>\Omega_{\mathrm{EE}}$

$\tau_{I2}>\tau_{I1}>\tau_{A}$: $\Omega_{\mathrm{IE}}>\Omega_{\mathrm{FA}},\Omega_{\mathrm{CC}}>\Omega_{\mathrm{EA}},\Omega_{\mathrm{FI}}>\Omega_{\mathrm{EE}}$

with $\tau_{A}=\frac{1}{1-p_{1}}\tau_{P}$, $\tau_{I1}=\frac{1}{p_{1}}\left( \tau_{S1}+\tau_{S2} \right)$, and $\tau_{I2}=\frac{1}{p_{1}}\frac{p_{2}}{1-p_{2}}\tau_{S2}$.

The fold change ($f_{c}=\frac{\upsilon\left( \left[ R \right]=\infty\right)}{\upsilon\left( \left[ R \right]=0 \right)}$) in the basic modes with *Ω* < *K*_d_ takes the following form:

$f_{c,i}=1+k_{i}\left( 1-\frac{\Omega}{K_{d}} \right)$, (F20)

where *i* represents regulation mode (*i* = EE, IE, EA, FA, FI, CC). $k_{\mathrm{EE}}=\frac{\tau_{\mathrm{AU}}}{\tau_{\mathrm{IU}}}$, $k_{\mathrm{IE}}=\frac{\tau_{\mathrm{IU}}}{\tau_{\mathrm{AU}}}$, $k_{\mathrm{EA}}=\frac{\frac{1}{p_{1}}\tau_{S1U}}{\tau_{\mathrm{AU}}+\frac{1}{p_{1}}\tau_{S2U}+\tau_{I2U}}$, $k_{\mathrm{FI}}=\frac{\frac{1}{p_{1}}\tau_{S2U}}{\tau_{\mathrm{AU}}+\frac{1}{p_{1}}\tau_{S1U}+\tau_{I2U}}$, $k_{\mathrm{FA}}=\frac{\tau_{I2U}}{\tau_{\mathrm{AU}}+\tau_{I1U}}$ and $k_{\mathrm{CC}}=\frac{\tau_{I2U}}{\tau_{\mathrm{AU}}+\tau_{I1U}}$. Clearly, the largest fold change is achieved via MEE with the same *Ω* in all modes for *τ*_AU_ *> τ*_IU_, and via MIE otherwise. For *Ω* > *K*_d_,

$f_{c,i}=\frac{\Omega}{K_{d}}$, (F21)

with *i* = IA and FC. In the same regulation mode, a large *Ω* leads to a small (large) fold change for *Ω* < *K*_d_ (*Ω* > *K*_d_).

The specificity *S* is defined as the ratio of average expression resulting from cognate binding (*υ*_1_) to that resulting from non-cognate binding (*υ*_0_):

$S\left( \left[ R \right] \right)=\frac{\upsilon_{1}\left( \left[ R \right] \right)}{\upsilon_{0}\left( \left[ R \right] \right)}=\frac{\upsilon_{max1}\frac{\beta_{1}+\left[ R \right]}{\Omega_{1}+\left[ R \right]}}{\upsilon_{max0}\frac{\beta_{0}+\left[ R \right]}{\Omega_{0}+\left[ R \right]}}$ (F22)

$S\left( \left[ R \right] \right)=\frac{\upsilon_{max1}}{\upsilon_{max0}}\frac{\beta_{1}+\left[ R \right]}{\beta_{0}+\left[ R \right]}\frac{\Omega_{0}+\left[ R \right]}{\Omega_{1}+\left[ R \right]}$, (F23)

where *υ*_max0_ (*υ*_max1_) is the maximum average transcription rate and $\upsilon=\upsilon_{max0}\frac{\beta_{0}}{\Omega_{0}}=\upsilon_{max1}\frac{\beta_{1}}{\Omega_{1}}$ is the basal transcription rate with unbound regulators. In eukaryotes, the dwell time of transcription factors at cognate binding sites is only two to three orders of magnitude longer than that at nonspecific DNA [5,6], leading to $\beta_{0}=\theta\beta_{0}^{'}$ and $\Omega_{0}=\theta\Omega_{0}^{'}$ in eight basic modes ($\theta$ is 100~1000). Therefore, *S* takes the following form:

$S\left( \left[ R \right] \right)=\frac{\upsilon_{max1}}{\upsilon_{max0}}\frac{\beta_{1}+\left[ R \right]}{\beta_{0}^{'}\theta+\left[ R \right]}\frac{\Omega_{0}^{'}\theta+\left[ R \right]}{\Omega_{1}+\left[ R \right]}.$ (F24)

For large $\theta$,

$S_{max}\approx S\left( \frac{\beta_{1}\left( \upsilon_{max1}-\upsilon_{max0} \right)+\beta_{1}\sqrt{\theta\left( \upsilon_{max1}-\upsilon\right)\left( \upsilon_{max0}-\upsilon\right)\frac{\Omega_{0}^{'}}{\beta_{1}}}}{(\upsilon_{max0}-\upsilon)} \right)$ (F25)

$S_{max}\approx f_{c1}$, (F26)

where $f_{c1}=\frac{\Omega_{1}}{\beta_{1}}$ is the fold change of average transcription rate with cognate binding. $S_{max}$ rises with increasing the fold change. According to Eqs. ([F20](#S20), F[26](#S26)), the anti-crosstalk capability of transcription is the strongest in MIE with (*Ω* < *K*_d_) [Fig. 2(D)].

In fact, regulators can regulate multiple parameters simultaneously, leading to a combinatorial regulation mode. The noise is between that in the modes making up the combined mode [Fig. S8]. When the mode is a combination of any two modes in {MEE, MEA, MFI, MCC}, or MIE and MEE, or MFC and MEE/MEA/MFI, the average transcription rate takes the following form:

$\upsilon=\frac{1}{a_{1}x_{1}+a_{2}x_{2}+a_{3}}$, (F27)

where *x*_i_ = *τ_i_* (*i* = P, S1, S2, S3; MEE/MEA/MFI/MCC), $\frac{1-p_{1}}{p_{1}}$ (MIE) or $\frac{p_{2}}{1-p_{2}}$ (MFC), while *a*_1_, *a*_2_ and *a*_3_ are constant coefficients.

$\upsilon=\frac{1}{a_{1}x_{1B}+a_{2}x_{2B}+a_{3}}\frac{\left( \left[ R \right]+b_{1}K_{d} \right)\left( \left[ R \right]+b_{2}K_{d} \right)}{\left[ R \right]^{2}+\left[ R \right]K_{d}\frac{a_{1}x_{1B}\left( 1+b_{2} \right)+a_{2}x_{2B}\left( 1+b_{1} \right)+a_{3}\left( b_{1}+b_{2} \right)}{\left( a_{1}x_{1B}+a_{2}x_{2B}+a_{3} \right)}+K_{d}^{2}b_{1}b_{2}\frac{a_{1}x_{1U}+a_{2}x_{2U}+a_{3}}{a_{1}x_{1B}+a_{2}x_{2B}+a_{3}}}$ (F28)

where *x*_iB_ and *x*_iU_ are the *x*_i_ with regulators bound and unbound, respectively. *b*_i_ is equal to $\frac{x_{iB}}{x_{iU}}(<1)$. Since *υ*([*R*]) is a monotonically increasing function, *υ*(*Ω_υ_*) < *υ*(*K*_d_) and *Ω_υ_* < *K*_d_ [Fig. S8].

When the mode is a combination of MIE and MEA/MFI/MCC/MFC, or MFC and MCC, the average transcription rate takes the following form:

$\upsilon=\frac{1}{a_{1}+a_{2}x_{1}\left( x_{2}+a_{3} \right)},$ (F29)

where $x_{1}=\frac{1-p_{1}}{p_{1}}$ (MIE) and *x*_2_ = *τ_i_* (i = S1, S2, S3; MEA, MFI, MCC) or $x_{2}=\frac{p_{2}}{1-p_{2}}$ (MFC), or $x_{1}=\frac{p_{2}}{1-p_{2}}$ (MFC) and *x*_2_ = *τ*_S3_. *a*_1_, *a*_2_ and *a*_3_ are constant coefficients.

$\upsilon=\frac{1}{a_{1}+a_{2}x_{1B}\left( x_{2B}+a_{3} \right)}\frac{\left( \left[ R \right]+b_{1}K_{d} \right)\left( \left[ R \right]+b_{2}K_{d} \right)}{\left[ R \right]^{2}+\left[ R \right]K_{d}\frac{a_{1}b_{2}+a_{1}b_{1}+a_{2}x_{1B}\left( 2b_{2}x_{2U}+a_{3}b_{2}+a_{3} \right)}{a_{1}+a_{2}x_{1B}\left( x_{2B}+a_{3} \right)}+K_{d}^{2}b_{1}b_{2}\frac{a_{1}+a_{2}x_{1U}\left( x_{2U}+a_{3} \right)}{a_{1}+a_{2}x_{1B}\left( x_{2B}+a_{3} \right)}}$ (F30)

*b*_i_ is equal to $\frac{x_{iB}}{x_{iU}}(<1)$. As *υ*([*R*]) is a monotonically increasing function, *υ*(*Ω_υ_*) < *υ*(*K*_d_) and *Ω_υ_* < *K*_d_ [Fig. [S8](#FIGS11)]. Meanwhile, the fold change of *υ* is large when the mode includes MIE and MEE.

In addition, the burst size in any mode takes the following form:

$b=b_{B}\frac{\left[ R \right]+K_{d}\frac{b_{U}}{b_{B}}}{\left[ R \right]+K_{d}},$ (F31)

where *b*_B_ and *b*_U_ are the burst size with regulators bound and unbound respectively. The operating point of burst size (*Ω*_b_) is *K*_d_. The burst frequency takes the following form:

$f=\frac{\upsilon}{b}$. (F32)

Given $\frac{\upsilon_{B}}{\upsilon_{U}}>1$, $\frac{b_{B}}{b_{U}}\geq1$ and $b\left( \Omega_{f} \right)\leq\frac{b_{U}+b(\Omega_{\mathrm{fmax}})}{2}$,

$\upsilon\left( \Omega_{f} \right)=b\left( \Omega_{f} \right)f\left( \Omega_{f} \right)\leq\frac{b_{U}+b\left( \Omega_{f\max} \right)}{2}\frac{f_{U}+f_{\max}}{2}\leq\frac{b_{U}f_{U}+b\left( \Omega_{f\max} \right)f_{\max}}{2}\leq\frac{b_{U}f_{U}+b_{B}f_{B}}{2}=\upsilon\left( \Omega_{\upsilon} \right)$, (F33)

$\Omega_{f}\leq\Omega_{\upsilon}$, (F34)

where *Ω_υ_*, *Ω*_b_ and *Ω_f_* are the operating points of average transcription rate, burst size and burst frequency. *Ω_f_*_max_ is [*R*] where *f* reaches the maximum. *Ω_f_* < *Ω_υ_* < *Ω*_b_ holds true when $\frac{\upsilon_{B}}{\upsilon_{U}}>1$, $\frac{b_{B}}{b_{U}}\geq1$ and $b\left( \Omega_{f} \right)\leq\frac{b_{U}+b(\Omega_{\mathrm{fmax}})}{2}$ [Fig. S8]. When *f*_max_ = *f*_B_, *Ω_f_* < *Ω_υ_* < *Ω*_b_ always holds true.

**Section 7: Mean and variance of mRNA numbers**

Given we know the changes in event durations, we further explore the mean and variance of mRNA numbers in steady state. The average number of mRNAs in steady state (<*m*>_SS_) can be expressed as

|  | ${\langle m\rangle}_{\mathrm{ss}}=\lim_{T\to\infty} \frac{1}{T}\int_{0}^{T} m(t)dt$, | (G1) |
| --- | --- | --- |
|  | ${\langle m\rangle}_{\mathrm{ss}}=\lim_{T\to\infty} \frac{1}{T}\int_{0}^{T} \left[ \sum_{t_{i}\leq t} \varepsilon\left( t-t_{i} \right)-\sum_{t_{i}+d_{i}\leq t} \varepsilon\left( {t-t}_{i}-d_{i} \right) \right]dt$, | (G2) |

where *ε* is a step function, and the *i*th mRNA is produced at *t_i_* and degrades at *t_i_* + *d_i_*. *d_i_* is a random number from the distribution $f_{d}\left( t \right)=\delta_{m}e^{-\delta_{m}t}$, where *δ*_m_ is the degradation rate constant of mRNA.

|  | $\left\langle m \right\rangle_{\mathrm{ss}}=\lim_{T\to\infty} \frac{1}{T}\left[ \sum_{t_{i}+d_{d,i}\leq T} d_{d,i}+\sum_{\begin{aligned} t_{i}+d_{d,i}>T \\ t_{i}\leq T \end{aligned}} T-t_{i} \right]=\lim_{T\to\infty} \frac{1}{T}\sum_{t_{i}+d_{d,i}\leq T} d_{d,i}$ | (G3) |
| --- | --- | --- |
|  | $\left\langle m \right\rangle_{\mathrm{ss}}=\lim_{n\to\infty} \frac{\sum_{i=1}^{n} \sum_{j=1}^{n_{i}} d_{ij}}{\sum_{i=1}^{n} T_{i}}$ | (G4) |
|  | $\left\langle m \right\rangle_{\mathrm{ss}}=\frac{\tau_{m}}{\tau_{P}+\frac{1-p_{1}}{p_{1}}\left( \tau_{S1}+\tau_{S2}+\frac{p_{2}}{1-p_{2}}\tau_{S3} \right)}$ | (G5) |
|  | $\upsilon=\frac{1}{\tau_{P}+\frac{1-p_{1}}{p_{1}}\left( \tau_{S1}+\tau_{S2}+\frac{p_{2}}{1-p_{2}}\tau_{S3} \right)}$, | (G6) |

where $T_{i}$ is the period of the *i*th burst in transcription and $d_{\mathrm{ij}}$ is the life of the *j*th mRNA in the *i*th burst. $n$ is the number of bursts and $n_{i}$ is the number of mRNAs produced during the *i*th burst. We assume that $\mathbf{G}\left( z;t \right)=\sum_{n=0}^{\infty} z^{n}\mathbf{P}\left( n;t \right)$ and $P\left( z;t \right)=\boldsymbol{u}_{l}\sum_{n=0}^{\infty} z^{n}\mathbf{P}\left( n;t \right)$, leading Eq. (A1) to be rewritten as

|  | $\frac{d\mathbf{G}\left( z;t \right)}{dt}=\left( \mathbf{K}^{\mathbf{S}}\boldsymbol{+}z\mathbf{K}^{P} \right)\mathbf{G}\left( z;t \right)+\delta_{m}\left( 1-z \right)\frac{d\mathbf{G}\left( z;t \right)}{dz}$. | (G7) |
| --- | --- | --- |

In steady state (d**G**(*z*;*t*)/d*t* = **0**) with *s = z*-1,

|  | $\left( \mathbf{K}^{\mathbf{S}}\boldsymbol{+}(s+1)\mathbf{K}^{P} \right)\mathbf{G}\left( s;t \right)-\delta_{m}s\frac{d\mathbf{G}\left( s;t \right)}{ds}=0$. | (G8) |
| --- | --- | --- |

When the process is decided by *τ*_P_*, τ*_S_, and *p*_1_ and *f*_i_ is an exponential distribution,

|  | $s\frac{d^{2}G\left( s \right)}{ds^{2}}+\left[ \frac{\frac{1}{\tau_{P}}\left( 1-p_{1} \right)+\frac{1}{\tau_{S}}p_{1}}{\delta_{m}}-\frac{\frac{1}{\tau_{P}}p_{1}}{\delta_{m}}s \right]\frac{dG\left( s \right)}{ds}-\frac{\frac{1}{\tau_{P}}\frac{1}{\tau_{S}}p_{1}}{\delta_{m}^{2}}G\left( s \right)=0$. | (G9) |
| --- | --- | --- |

Owing to $\left. G\left( z \right) \right|_{z=1}=1$ and $\left. G\left( z \right) \right|_{z=0}=0$,

| $G\left( z \right)=\sum_{n=0}^{\infty} \frac{\Gamma\left( \frac{\tau_{m}}{\tau_{S}}+n \right)\Gamma\left( \frac{\tau_{m}}{\tau_{P}}\left( 1-p_{1} \right)+\frac{\tau_{m}}{\tau_{S}}p_{1} \right)}{\Gamma\left( \frac{\tau_{m}}{\tau_{S}} \right)\Gamma\left( \frac{\tau_{m}}{\tau_{P}}\left( 1-p_{1} \right)+\frac{\tau_{m}}{\tau_{S}}p_{1}+n \right)}\frac{\left( \frac{\tau_{m}}{\tau_{P}}p_{1} \right)^{n}}{n!}{}_{1}{F_{1}}\left( \frac{\tau_{m}}{\tau_{S}}+n,\frac{\tau_{m}}{\tau_{P}}\left( 1-p_{1} \right)+\frac{\tau_{m}}{\tau_{S}}p_{1}+n,-\frac{\tau_{m}}{\tau_{P}}p_{1} \right)z^{n}$. |
| --- |
| (G10) |

Therefore, $P\left( m \right)$ takes the following forms:

|  | $P\left( m \right)=\frac{1}{m!}\left. \frac{d^{m}G(z)}{dz^{m}} \right\vert_{z=0}$ | (G11) |
| --- | --- | --- |
| $P\left( m \right)=\frac{\Gamma\left( \frac{\tau_{m}}{\tau_{S}}+m \right)\Gamma\left( \frac{\tau_{m}}{\tau_{P}}\left( 1-p_{1} \right)+\frac{\tau_{m}}{\tau_{S}}p_{1} \right)}{\Gamma\left( \frac{\tau_{m}}{\tau_{S}} \right)\Gamma\left( \frac{\tau_{m}}{\tau_{P}}\left( 1-p_{1} \right)+\frac{\tau_{m}}{\tau_{S}}p_{1}+m \right)}\frac{\left( \frac{\tau_{m}}{\tau_{P}}p_{1} \right)^{m}}{m!}{}_{1}{F_{1}}\left( \frac{\tau_{m}}{\tau_{S}}+m,\frac{\tau_{m}}{\tau_{P}}\left( 1-p_{1} \right)+\frac{\tau_{m}}{\tau_{S}}p_{1}+m,-\frac{\tau_{m}}{\tau_{P}}p_{1} \right)$. | | |
| (G12) | | |

The mean and variance of mRNA levels are expressed as

|  | $\left\langle m \right\rangle=G^{'}\left( 1 \right)$, | (G13) |
| --- | --- | --- |
|  | $\sigma_{m}^{2}=G^{''}\left( 1 \right)+G^{'}\left( 1 \right)-\left[ G^{'}\left( 1 \right) \right]^{2}$. | (G14) |

We further obtain

|  | $\left\langle m \right\rangle=\frac{1}{\frac{\tau_{P}}{\tau_{m}}+\frac{1-p_{1}}{p_{1}}\frac{\tau_{S}}{\tau_{m}}}$, | (G15) |
| --- | --- | --- |
|  | $\sigma_{m}^{2}=\left\langle m \right\rangle+\left\langle m \right\rangle^{2} \frac{\left( \frac{\tau_{I}}{\tau_{m}}+\frac{\tau_{A}}{\tau_{m}} \right)\frac{\tau_{S}}{\tau_{m}}-\frac{\tau_{A}}{\tau_{m}}\frac{\tau_{I}}{\tau_{m}}}{\frac{\tau_{I}}{\tau_{m}}+\frac{\tau_{A}}{\tau_{m}}+\frac{\tau_{A}}{\tau_{m}}\frac{\tau_{I}}{\tau_{m}}}$, | (G16) |
|  | $\eta_{m}^{2}=\frac{1}{\left\langle m \right\rangle}+\frac{\left( \frac{\tau_{I}}{\tau_{m}}+\frac{\tau_{A}}{\tau_{m}} \right)\frac{\tau_{S}}{\tau_{m}}-\frac{\tau_{A}}{\tau_{m}}\frac{\tau_{I}}{\tau_{m}}}{\frac{\tau_{I}}{\tau_{m}}+\frac{\tau_{A}}{\tau_{m}}+\frac{\tau_{A}}{\tau_{m}}\frac{\tau_{I}}{\tau_{m}}}$. | (G17) |

When *τ*_P_, *τ*_S_ and *p*_1_ are regulated alone, the variances are

|  | $\sigma_{m,\tau_{P}}^{2}=\left\langle m \right\rangle-\left\langle m \right\rangle^{2} \frac{\left\langle m \right\rangle-\frac{\tau_{m}}{\tau_{I}}}{\left\langle m \right\rangle-\frac{1}{1-p_{1}}\left( 1+\frac{\tau_{I}}{\tau_{m}} \right)\frac{\tau_{m}^{2}}{\tau_{I}^{2}}}$, | (G18) |
| --- | --- | --- |
|  | $\sigma_{m,\tau_{S}}^{2}=\left\langle m \right\rangle-\left\langle m \right\rangle\frac{\left( \left\langle m \right\rangle-\frac{\tau_{m}}{\tau_{P}} \right)\left( \left\langle m \right\rangle-\frac{\tau_{m}}{\tau_{P}}p_{1} \right)}{\left\langle m \right\rangle-\left( 1-p_{1} \right)\frac{\tau_{m}^{2}}{\tau_{P}^{2}}-\frac{\tau_{m}}{\tau_{P}}}$, | (G19) |
|  | $\sigma_{m, p_{1}}^{2}=\left\langle m \right\rangle-\left\langle m \right\rangle^{2} \frac{\left\langle m \right\rangle^{2}-\frac{\tau_{m}}{\tau_{P}}\frac{\tau_{S}-2\tau_{P}}{\tau_{S}-\tau_{P}}\left\langle m \right\rangle-\frac{\tau_{m}}{\tau_{S}-\tau_{P}}\frac{\tau_{m}}{\tau_{P}}}{\left\langle m \right\rangle^{2}+\left( \frac{\tau_{m}}{\tau_{P}}+2 \right)\frac{\tau_{m}}{\tau_{S}-\tau_{P}}\left\langle m \right\rangle+\frac{\tau_{m}+\tau_{P}}{\tau_{P}}\left( \frac{\tau_{m}}{\tau_{S}-\tau_{P}} \right)^{2}}$. | (G20) |

The corresponding Fano factor ($\sigma_{m}^{2}/\langle m\rangle$) is separately

|  | $F_{m,\tau_{P}}=1-\left\langle m \right\rangle\frac{\left\langle m \right\rangle-\frac{\tau_{m}}{\tau_{I}}}{\left\langle m \right\rangle-\frac{1}{1-p_{1}}\left( 1+\frac{\tau_{I}}{\tau_{m}} \right)\frac{\tau_{m}^{2}}{\tau_{I}^{2}}}$, | (G21) |
| --- | --- | --- |
|  | $F_{m,\tau_{S}}=1-\frac{\left( \left\langle m \right\rangle-\frac{\tau_{m}}{\tau_{P}} \right)\left( \left\langle m \right\rangle-\frac{\tau_{m}}{\tau_{P}}p_{1} \right)}{\left\langle m \right\rangle-\left( 1-p_{1} \right)\frac{\tau_{m}^{2}}{\tau_{P}^{2}}-\frac{\tau_{m}}{\tau_{P}}}$, | (G22) |
|  | $F_{m, p_{1}}=1-\left\langle m \right\rangle\frac{\left\langle m \right\rangle^{2}-\frac{\tau_{m}}{\tau_{P}}\frac{\tau_{S}-2\tau_{P}}{\tau_{S}-\tau_{P}}\left\langle m \right\rangle-\frac{\tau_{m}}{\tau_{S}-\tau_{P}}\frac{\tau_{m}}{\tau_{P}}}{\left\langle m \right\rangle^{2}+\left( \frac{\tau_{m}}{\tau_{P}}+2 \right)\frac{\tau_{m}}{\tau_{S}-\tau_{P}}\left\langle m \right\rangle+\frac{\tau_{m}+\tau_{P}}{\tau_{P}}\left( \frac{\tau_{m}}{\tau_{S}-\tau_{P}} \right)^{2}}$. | (G23) |

In addition, the mean and variance of mRNAs with more complex distribution $f_{i}$ differ slightly compared with the case of the simplest situation [Figs. S5 and S6]. Thus, we can use the simplest process to simulate the transcription.

If *F* is close to 1, *p*_1_ → ∞ or *τ*_S_ → 0, then <*m*> → *τ*_m_/*τ*_P_. When only *τ*_S_ is regulated with fixed *τ*_P_, the maximum of *F* is close to 1 + *τ*_m_/*τ*_P_ with *τ*_S_ → 0. When *p*_1_ is regulated with fixed *τ*_P_, the maximum of *F* is smaller than 1 + *τ*_m_/*τ*_P_.

**Section 8: Transient response to stimulation**

It is important to know how fast transcription responds to regulator stimulation. The probability that the gene is in the active phase at time *t* is

|  | $P_{A}\left( t \right)=f_{I\_init}\left( t \right)*\delta\left( t \right)\sum_{r=0}^{\infty} \left[ *f_{A}\left( t \right)*f_{I}\left( t \right) \right]^{r}*\left[ 1-\int_{0}^{t} f_{A}\left( \tau\right)d\tau\right]$. | (H1) |
| --- | --- | --- |

*f*_A_ and *f*_I_ are the duration distributions of the active and inactive phase in steady state after stimulation, *f*_I_init_ (*t*) is the distribution of time required for the gene to enter the active phase, and $*$represents convolution. If burst size is large, *f*_A_ is close to an exponential distribution, Eq. (G1) is written as

|  | $P_{A}\left( t \right)\approx f_{I\_init}\left( t \right)*\delta\left( t \right)\sum_{r=0}^{\infty} \left[ *f_{A}\left( t \right)*f_{I}\left( t \right) \right]^{r}*e^{-\frac{t}{\tau_{A}}}$. | (H2) |
| --- | --- | --- |

When *f*_I_init_(*t*) is close to *f*_I­_(*t*), *P*_A_(*t*) does not overshoot. *P*_A_(*t*) may overshoot when *f*_I_init_(*t*) is different from *f*_I­_(*t*) [Fig. S11]. The presence of only one rate-limiting step in transcription cycle often means that *f*_I_init_(*t*) is close to *f*_I_ (*t*). <*m*>(t) is the average number of mRNA copies, satisfying

|  | $\frac{d\langle m\rangle(t)}{\mathrm{dt}}=\frac{1}{\tau_{P}}P_{A}\left( t \right)-\frac{\langle m\rangle\left( t \right)}{\tau_{m}}$. | (H3) |
| --- | --- | --- |

Thus,

|  | $\langle m\rangle\left( t \right)=e^{-\frac{t}{\tau_{m}}}*\left[ \frac{1}{\tau_{P}}P_{A}\left( t \right)-\frac{\langle m\rangle\left( 0 \right)}{\tau_{m}} \right]+\langle m\rangle\left( 0 \right)$, | (H4) |
| --- | --- | --- |

where 1/*τ*_P_ is the transcription rate constant and *τ*_m_ is the degradation rate constant. Considering a limiting case with a completely silent initial state,

|  | $\langle m\rangle\left( t \right)=\frac{1}{\tau_{P}}e^{-\frac{t}{\tau_{m}}}*f_{I\_init}\left( t \right)*\delta\left( t \right)\sum_{r=0}^{\infty} \left[ *f_{A}\left( t \right)*f_{I}\left( t \right) \right]^{r}*e^{-\frac{t}{\tau_{A}}}$. | (H5) |
| --- | --- | --- |

The mRNA response curve,$\langle m\rangle(t)$, is

$\langle m\rangle\left( t \right)=f_{I\_\mathrm{init}}\left( t \right)*F\left( t \right), F\left( t \right)=\frac{1}{\tau_{P}}e^{-\frac{t}{\tau_{m}}}*\delta\left( t \right)\sum_{r=0}^{\infty} \left[ *f_{A}\left( t \right)*f_{I}\left( t \right) \right]^{r}*e^{-\frac{t}{\tau_{A}}}.$(H6)

When the model parameters are the same as in steady state, $F\left( t \right)$ is identical for all regulatory modes and$f_{I\_\mathrm{init}}\left( t \right)$decides the difference in$\langle m\rangle\left( t \right)$ because of the delay. In most cases, the smaller the mean ($\tau_{I\_init}$) and CV ($\tau_{I\_\mathrm{init}}$) of $f_{I\_\mathrm{init}}\left( t \right)$ are, the more easy it is for mRNA levels to exceed the steady-state value [Fig. S11]. When the initial state is completely silent and the steady states of different modes after stimulation are the same, a long average duration ($\tau_{I\_\mathrm{init}}$) of $f_{I\_\mathrm{init}}\left( t \right)$ leads to a large response time ($t_{\mathrm{re}}$, denoting the time when half the mRNA level in steady state is reached).

In MEE,

$f_{I\_\mathrm{init}\_\mathrm{MEE}}\left( t \right)=\delta\left( t \right)$, (H7)

$\tau_{I\_\mathrm{init}\_\mathrm{MEE}}=0$. (H8)

In$\mathrm{MEA}$,

$f_{I\_\mathrm{init}\_\mathrm{MEA}}\left( t \right)=f_{I}\left( t \right)$, (H9)

$\tau_{I\_\mathrm{init}\_\mathrm{MEA}}=\tau_{I}$. (H10)

In$\mathrm{MFI}$,

$f_{I\_\mathrm{init}\_\mathrm{MFI}}\left( t \right)=p_{1}f_{S2}\left( t \right)\sum_{i=0}^{\infty} \left( 1-p_{2} \right)p_{2}^{i}\left[ {*f}_{S3}\left( t \right) \right]^{i}+\left( 1-p_{1} \right)f_{I}\left( t \right)$, (H11)

$\tau_{I\_\mathrm{init}\_\mathrm{FI}}=\tau_{I}-\tau_{S1}.$ (H12)

In $\mathrm{MFA}$ and$\mathrm{MCC}$,

$f_{I\_\mathrm{init}\_\mathrm{MFA}}\left( t \right)\approx f_{I\_\mathrm{init}\_\mathrm{MCC}}\left( t \right)=f_{S3}\left( t \right)*f_{I\_\mathrm{init}\_\mathrm{MFI}}\left( t \right)$ , (H13)

$\tau_{I\_\mathrm{init}\_\mathrm{MFA}}\approx\tau_{I\_\mathrm{init}\_\mathrm{MCC}}=\tau_{I}-\tau_{S1}+\tau_{S3}.$ (H14)

In MIE,

| $f_{I\_\mathrm{init}\_\mathrm{MIE}}\left( t \right)=\frac{\tau_{S1}+\tau_{S2}}{\tau_{S1}+\tau_{S2}+\frac{p_{2}}{1-p_{2}}\tau_{S3}}f_{I\_\mathrm{init}\_\mathrm{MEA}}\left( t \right)+\frac{\tau_{S2}}{\tau_{S1}+\tau_{S2}+\frac{p_{2}}{1-p_{2}}\tau_{S3}}f_{I\_\mathrm{init}\_\mathrm{MFI}}\left( t \right)+\frac{\frac{p_{2}}{1-p_{2}}\tau_{S3}}{\tau_{S1}+\tau_{S2}+\frac{p_{2}}{1-p_{2}}\tau_{S3}}f_{I\_\mathrm{init}\_\mathrm{MCC}}\left( t \right)$, |
| --- |
| (H15) |

$\tau_{I\_\mathrm{init}\_\mathrm{MIE}}=\tau_{I}-\frac{\tau_{S1}+\frac{p_{2}}{1-p_{2}}\tau_{S3}}{\tau_{S1}+\tau_{S2}+\frac{p_{2}}{1-p_{2}}\tau_{S3}}\tau_{S1}+\frac{\frac{p_{2}}{1-p_{2}}\tau_{S3}}{\tau_{S1}+\tau_{S2}+\frac{p_{2}}{1-p_{2}}\tau_{S3}}\tau_{S3}.$ (H16)

The first and second fastest responses are realized via MEE and MFI respectively. Moreover, $\tau_{I\_\mathrm{init}\_MIE+MEA}$ = $\tau_{I\_\mathrm{init}\_\mathrm{MEA}}$, $\tau_{I\_\mathrm{init}\_MIE+MFI}$ = $\tau_{I\_\mathrm{init}\_\mathrm{MFI}}$, $\tau_{I\_\mathrm{init}\_MIE+MFA}$ = $\tau_{I\_\mathrm{init}\_\mathrm{MFA}}$ and $\tau_{I\_\mathrm{init}\_MIE+MCC}$ = $\tau_{I\_\mathrm{init}\_\mathrm{MCC}}$, meaning that adding MIE has no effect on the response time for MEA, MFI, MFA and MCC.


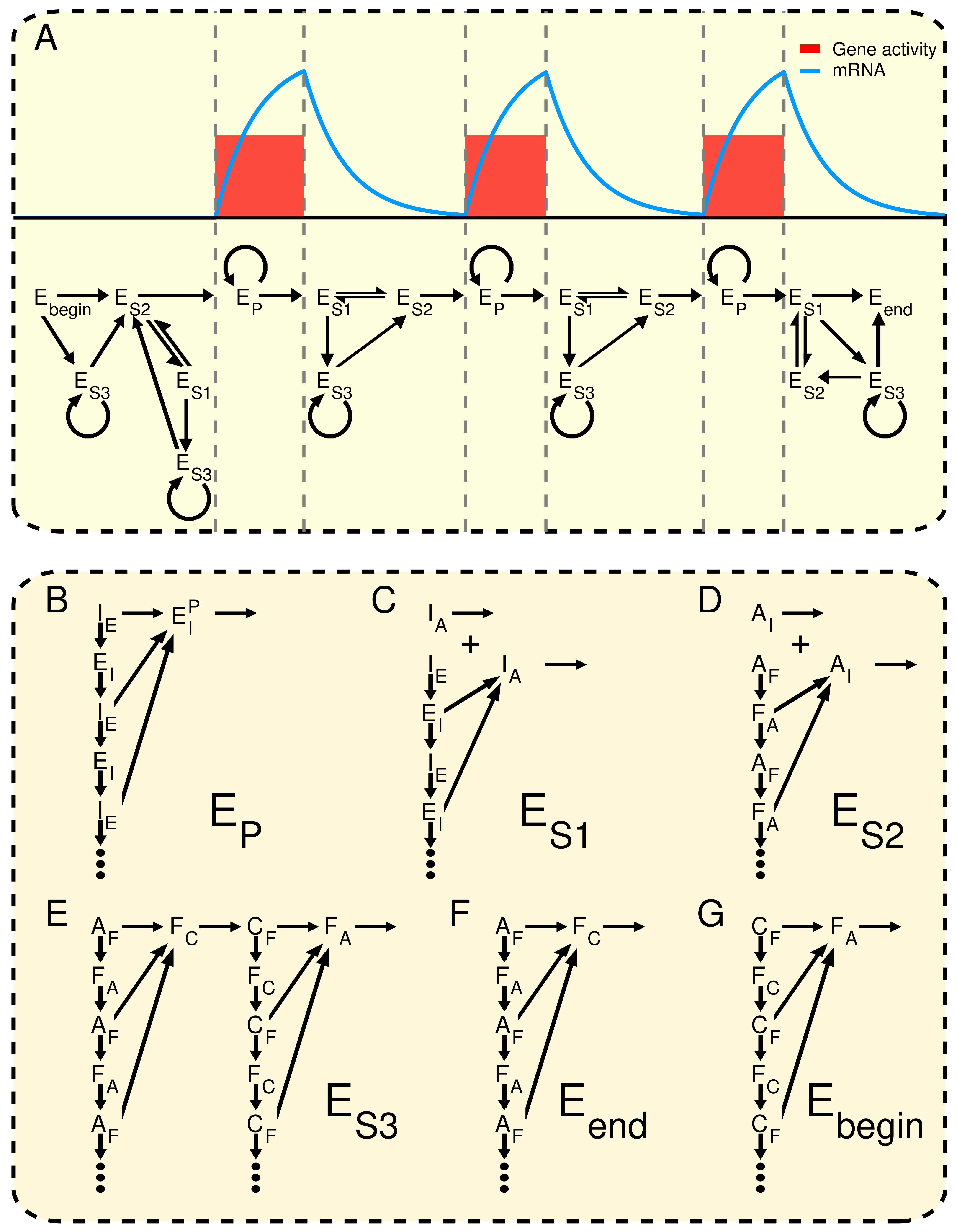


**Figure** **S1**. Events in transcriptional bursting. (**A**) Schematic of three bursts and the corresponding events. The transcription begins with $E_{\mathrm{begin}}$ and ends with $E_{\mathrm{end}}$. The adjacent active and inactive phases, which are separated by grey dotted lines, constitute a complete burst. (**B-G**) State transitions underlying events $E_{P},E_{S1},E_{S2},E_{S3},E_{\mathrm{end}}$ and $E_{\mathrm{begin}}$. Each event contains many paths, involving different combinations of $I_{E},E_{I},E_{I}^{P},I_{A},A_{I},A_{F},F_{A},F_{C}$ and $C_{F}$.


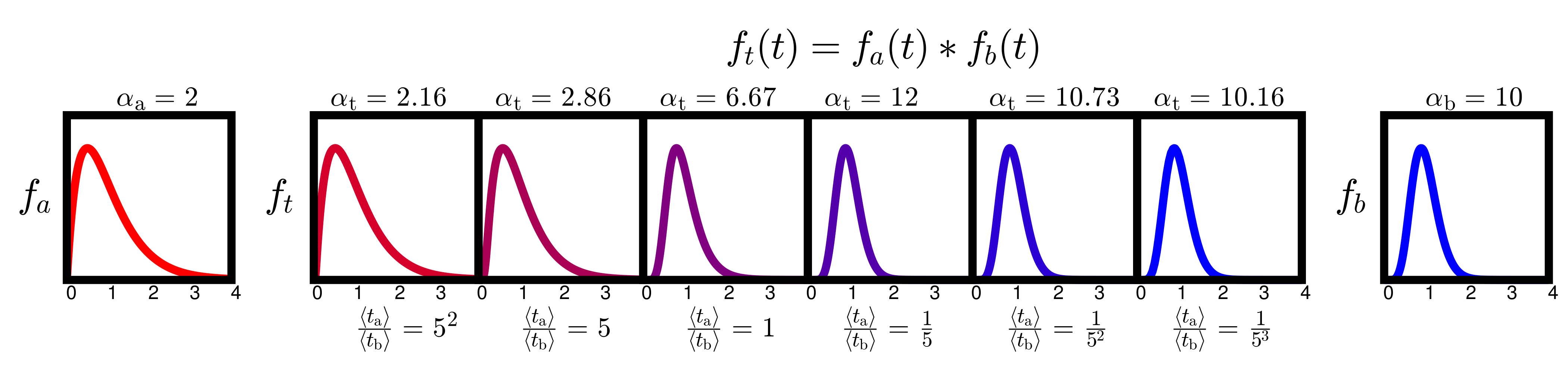


**Figure** **S2.** Duration distribution of the process that consists of two sequential processes. $f_{t}\left( t \right)=f_{a}\left( t \right)*f_{b}\left( t \right)$. $f_{a}\left( t \right)=\Gamma\left( t,\alpha_{a},\beta_{a} \right)$ (red; $\Gamma\left( t,\alpha,\theta\right)=\frac{\beta^{\alpha}}{\Gamma\left( \alpha\right)}t^{\alpha-1}e^{-\beta t}$) with $\alpha_{a}=2$, $\beta_{a}=\frac{\alpha_{a}}{\langle t_{a}\rangle}$ and $f_{b}\left( t \right)=\Gamma\left( t,\alpha_{b},\theta_{b} \right)$ (blue) with *α*_b_ = 10 and $\beta_{b}=\frac{\alpha_{b}}{\langle t_{a}\rangle}$, where $\langle t_{a}\rangle$ and $\langle t_{b}\rangle$ are separately the means of $f_{a}\left( t \right)$ and $f_{b}\left( t \right)$. The time is normalized by their respective average time in all curves. When $\frac{\left\langle t_{a} \right\rangle}{\langle t_{b}\rangle}$ tends toward 0, $\alpha_{t}$ tends toward $\alpha_{a}$. On the contrary, if $\frac{\left\langle t_{a} \right\rangle}{\left\langle t_{b} \right\rangle}$ tends toward ∞, *α*_t_ tends to be *α*_b_. Only when $\frac{\left\langle t_{a} \right\rangle}{\langle t_{b}\rangle}=\frac{\alpha_{a}}{\alpha_{b}}$, *α*_t_ takes the maximum *α_a_*+*α*_b_.


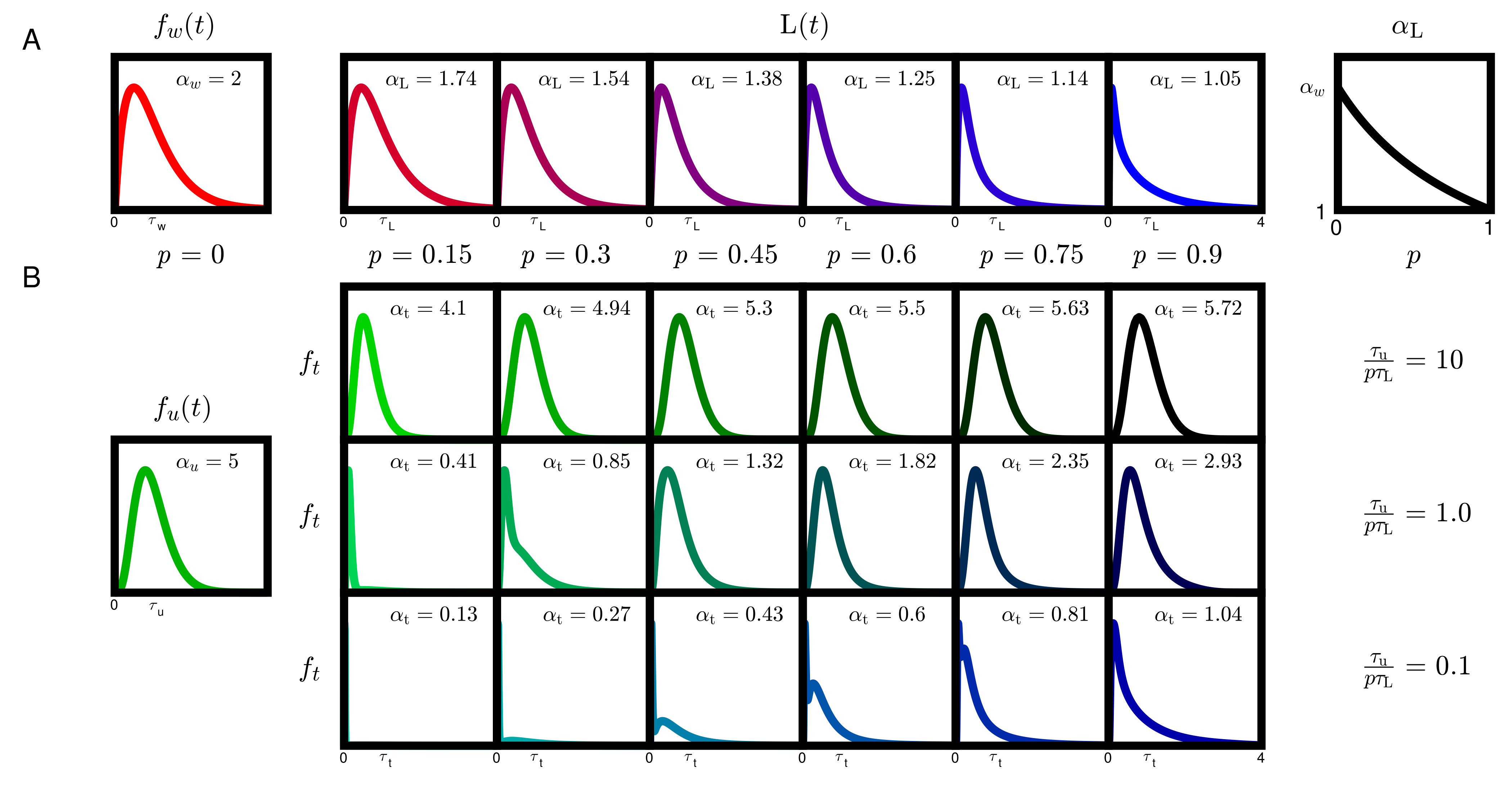


**Figure** **S3.** Duration distribution of a mixed process. (**A**) Duration distribution $L\left( t \right)=\frac{1-p}{p}\sum_{i=1}^{\infty} p^{i}\delta\left( t \right)\left[ *w\left( t \right) \right]^{i}$ ($*$ represents convolution), such as the distribution of total durations of *E*_S3_ in a burst. *w*(*t*) is the duration distribution of an event with repetition probability *p* (e.g., *E*_S3_ in the event model), equaling $\frac{1}{\tau_{w}}e^{-\frac{t}{\tau_{w}}}$ with *τ*_w_ = 1. *τ*_i_ is the mean duration and *α*_i_ = 1/CV_i_^2^ (*i* = t, L, w, u), where CV_i_ is the CV of *f*_i_(*t*). With increasing the probability$p$, *α*_L_ decreases from *α*_w_ to 1 and *L*(*t*) tends to be an exponential distribution. (**B**) Duration distribution $f_{t}\left( t \right)=\left( 1-p \right)u\left( t \right)+pu\left( t \right)*L\left( t \right)$, such as the duration distribution of the inactive phase in the event model. When $p$ is close to 1, *α*_t_ tends toward *α*_u_+1 for large $\frac{\tau_{u}}{p\tau_{L}}$, whereas *α*_t_ is close to 1 and *f*(*t*) tends toward an exponential function for small $\frac{\tau_{u}}{{p\tau}_{L}}$.

**
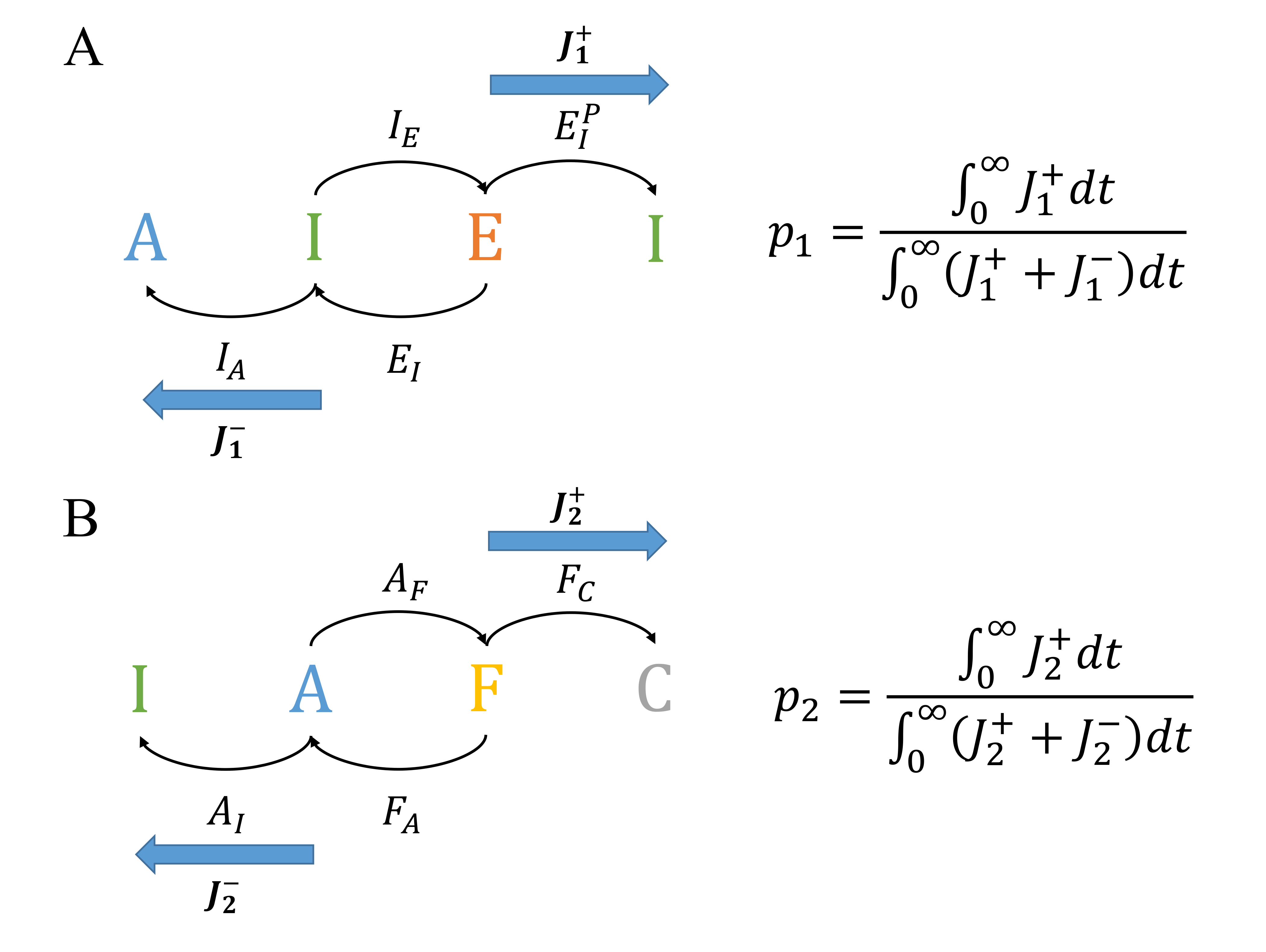
**

**Figure** **S4.** Probability of repeating events $E_{P}$ and $E_{S3}$. $p_{1}$ and $p_{2}$separately denote the probability of repeating events $E_{P}$ and $E_{S3}$. (**A**) $p_{1}$ is the probability decided by the probability flow $J_{1}^{+}$ and $J_{1}^{-}$ ($p_{1}=\frac{\int_{0}^{\infty} J_{1}^{+}dt}{\int_{0}^{\infty} \left( J_{1}^{+}+J_{1}^{-} \right)dt}$), where $J_{1}^{+}$ is the probability flow that the gene state transitions from set *I* to *E* and back to *I* with a Pol II entering productive elongation, and $J_{1}^{-}$ is the probability flow that the state converts from set *E* to *A* without Pol II entering productive elongation. (**B**) $p_{2}$ is the probability decided by the probability flow $J_{2}^{+}$ and $J_{2}^{-}$ ($p_{2}=\frac{\int_{0}^{\infty} J_{2}^{+}dt}{\int_{0}^{\infty} \left( J_{2}^{+}+J_{2}^{-} \right)dt}$), where $J_{2}^{+}$ is the probability flow that the gene state transitions from set *A* to *C*, and $J_{2}^{-}$ is the probability flow that the state converts from set *F* to *I*.

**
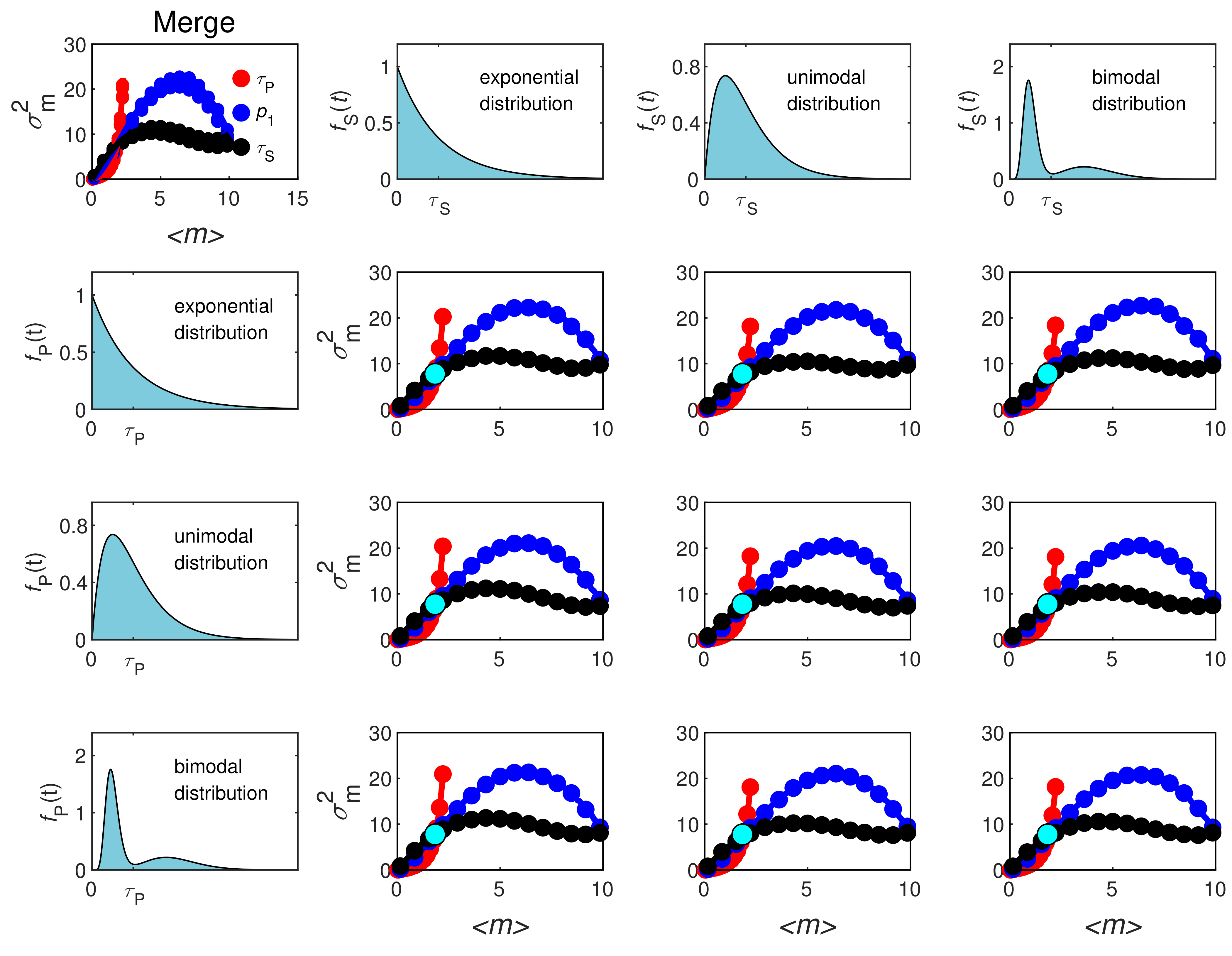
Figure** **S5.** Relationships between the mean (<*m>*) and variance (*σ*_m_^2^) of mRNA numbers for different distributions of *f*_P_ and *f*_S_. Three regulatory modes are simulated here; only *τ*_P_ (red), *p*_1_(blue) or *τ*_S_ (black) is regulated alone, with the other parameters fixed at default values (*p*_1_ = 0.9, *τ*_P_ = 0.5 min, *τ*_S_ = 20 min and *τ*_m_ = 5 min; shy blue point). There exist three distribution forms (exponential, unimodal and bimodal) for *f*_P_ (three panels in the leftmost column) and *f*_P_ (three panels on the top row). The lines are fitting curves to simulation data. Nine combinations are merged in the top left panel, where the lines are from analytical expressions [Eqs. (E18-E20)] with exponential distributions of *f*_P_ and *f*_S_. The concrete forms of *f*_P_ and *f*_S_ have little effect on the curve of *σ*_m_^2^ versus <*m>*.


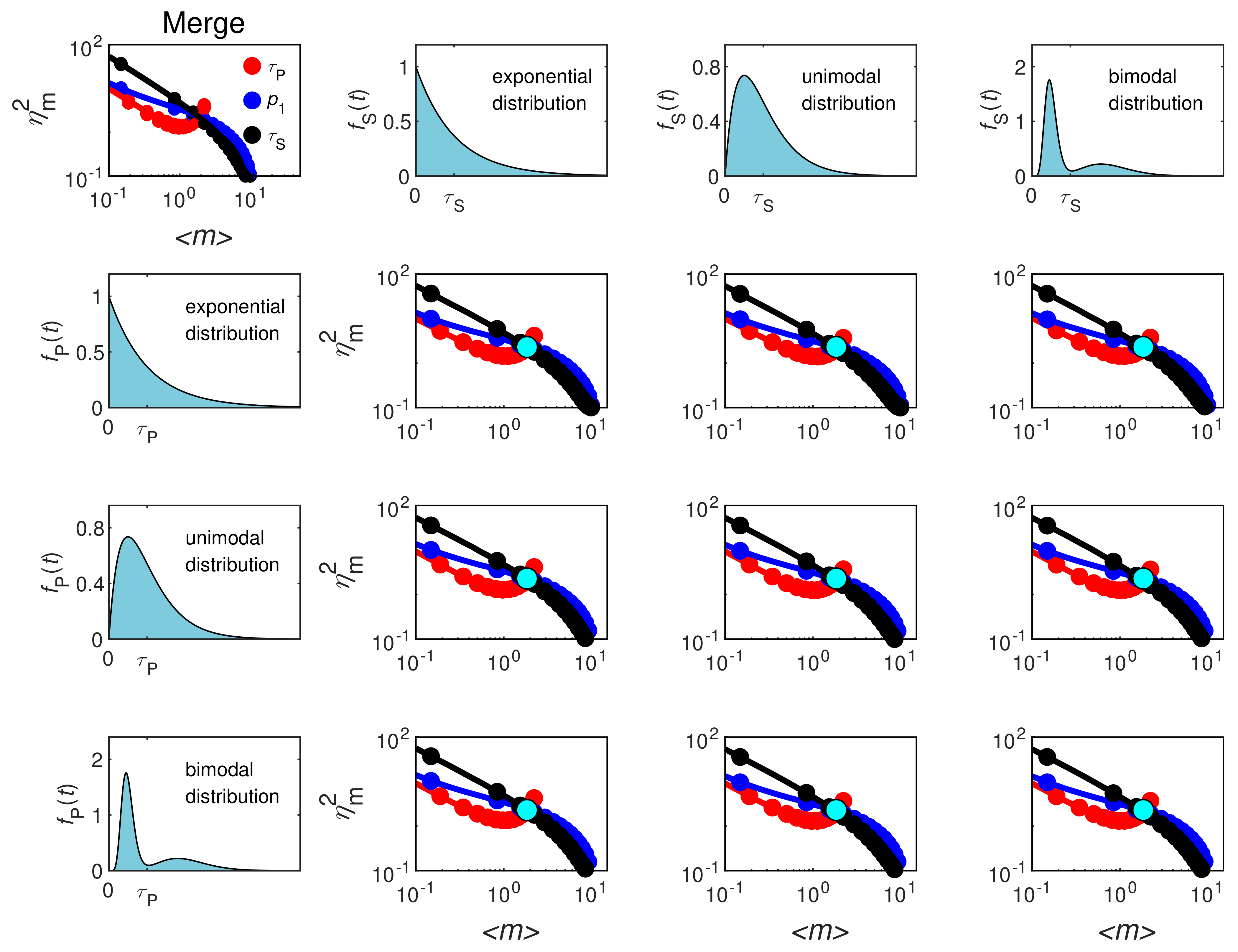


**Figure** **S6.** Relationships between the mean (<*m>*) and relative noise strength ($\eta_{m}^{2}=\sigma_{m}^{2}/{\langle m\rangle}^{2}$) of mRNA numbers for different *f*_P_ and *f*_S_. The data are from the same simulations as in Fig. S5, and the same notations are used as in Fig. S5.


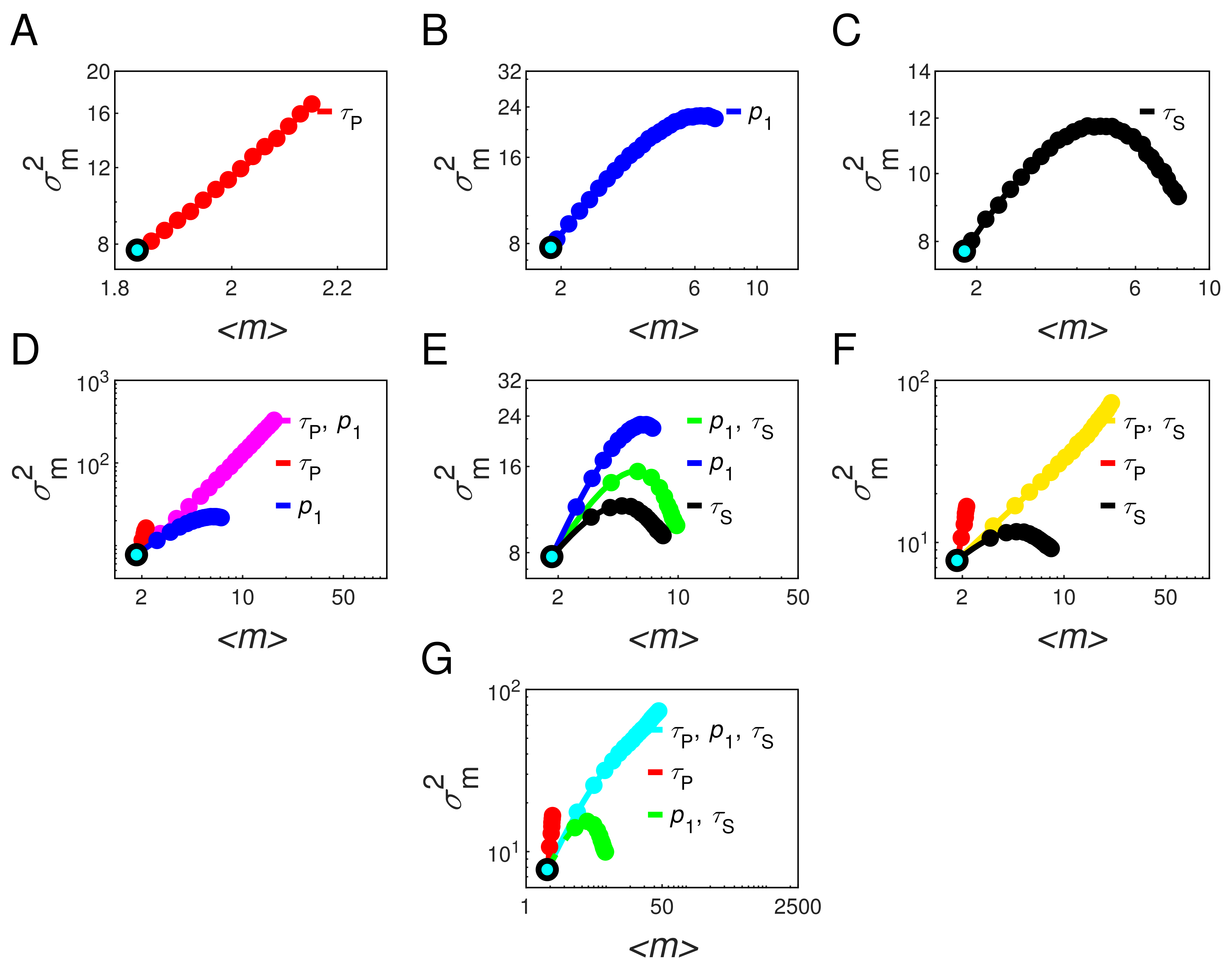


**Figure** **S7.** Relationships between the mean (<*m>*) and variance (*σ*_m_^2^) of mRNA numbers under different regulatory modes. The binding of regulatory factors promotes mRNA production. Only the following parameter(s) are modulated for each curve: (**A**) *τ*_P_ (red), (**B**) *p*_1_ (blue), (**C**) *τ*_S_ (black), (**D**) *τ*_P_ and *p*_1_ (pink), (**E**) *p*_1_ and *τ*_S_ (green), (**F**) *τ*_P_ and *τ*_S_ (yellow), and (**G**) *τ*_P_, *τ*_S_ and *p*_1_ (sky blue). The open sky-blue circle represents the case without DNA-bound regulators. The default parameters are *p*_1_ = 0.9, *τ*_P_ = 0.5 min, *τ*_S_ = 20 min, and *τ*_m_ = 5 min (without DNA-bound regulators). When *τ*_P_ (*τ*_S_ or *p*_1_) is regulated, *τ*_PB_ = 0.1 min (*τ*_SB_ = 1 min or *p*_1B_ = 0.99), and the others are fixed.


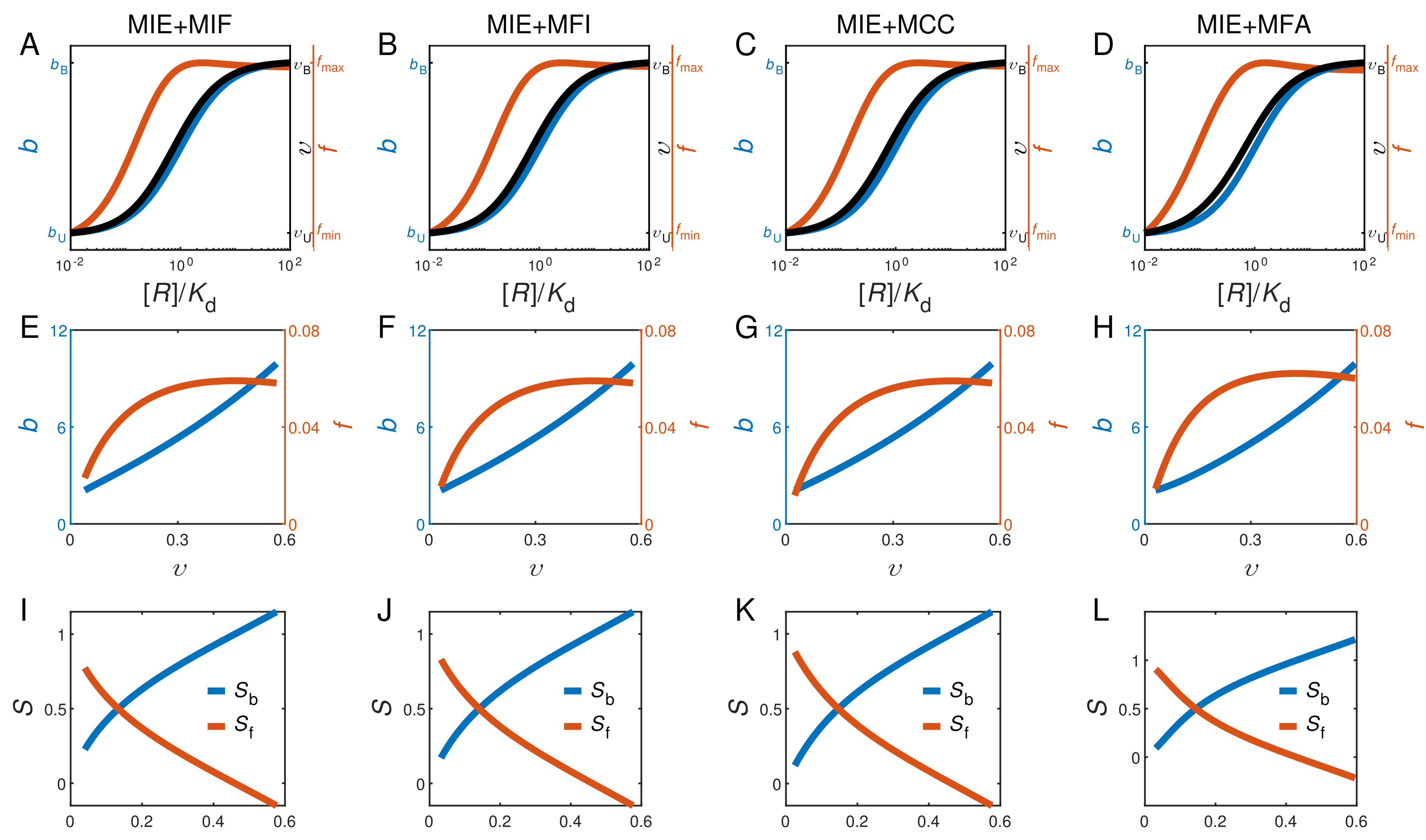


**Figure** **S8.** Transcriptional kinetics under different combination modes. (**A, E, I**) MIE+MIF with τ_S1U_ = 20 min, τ_S1B_ = 1 min, τ_S2_ = 5 min, τ_S3_ = 5 min, and *p*_2_ = 0.5. (**B, F, J**) MIE+MFI with τ_S1_ = 5 min, τ_S2U_ = 30 min, τ_S2B_ = 1 min, τ_S3_ = 5 min, and *p*_2_ = 0.5. (**C, G, K**) MIE+MCC with τ_S1_ = 5 min, τ_S2_ = 5 min, τ_S3U_ = 50 min, τ_S3B_ = 1 min, and *p*_2_ = 0.5. (**D, H, L**) MIE+MFA with τ_S1_ = 5 min, τ_S2_ = 5 min, τ_S3_ = 5 min, *p*_2U_ = 0.5, and *p*_2B_ = 0.01. Meanwhile, τ_P_ = 0.5 min, *p*_1U_ = 0.5, and *p*_1B_ = 0.9 for all modes. (**A-D**) Burst size (*b*, blue), burst frequency (*f*, red), and average transcription rate (*υ*, black) versus the regulator concentration. (**E-H**) Burst size and burst frequency versus the average transcription rate. (**I-L**) $S_{f}=\frac{d\ln f}{d\ln\upsilon}\mathrm{and} S_{b}=\frac{db}{d\ln\upsilon}$ ($S_{f}+S_{b}=1$) versus the average transcription rate.


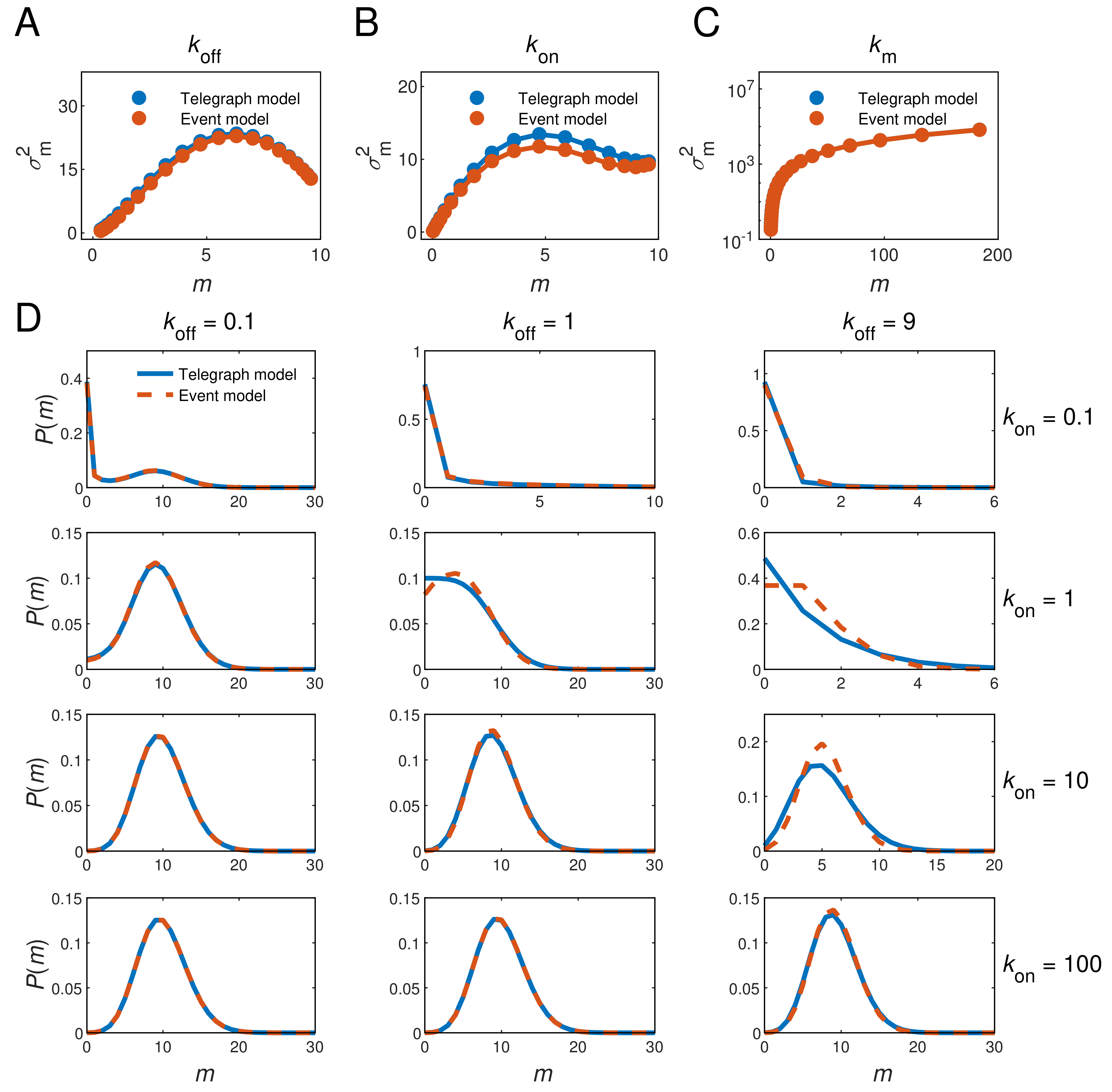


**Figure** **S9.** Difference between the telegraph and event models under different regulatory modes. The mean transcription rate and duration of the active (inactive) phase are identical in both models. Default parameters are *k*_on_ = 0.045 min^-1^, *k*_off_ = 0.2 min^-1^, *k*_m_ = 2 min^-1^, and *δ*_m_ = 0.2 min^-1^. *p*_1_ = 1-*k*_off_/*k*_m_, *τ*_P_ = 1/*k*_m_, and *τ*_S_ = *p*_1_/*k*_on_ (*f*_P_ and *f*_S_ are exponentially distributed). (**A**) Frequency modulation with $k_{\mathrm{on}}$ regulated (multiplied by 21 logarithmically spaced values between 10^-2^ and 10^2^) and other parameters unchanged. (**B**) Duration modulation with $k_{\mathrm{off}}$ regulated (multiplied by 21 logarithmically spaced values between 10^-2^ and 10^0.2^), 0 < *p*_1_ < 1 and other parameters unchanged. (**C**) Amplitude modulation with *k*_m_ regulated (multiplied by 21 logarithmically spaced values between 10^-0.8^ and 10^2^), 0 < *p*_1_ < 1 and other parameters unchanged. The difference between the two models is obvious when *k*_off_ and *k*_on_ are regulated with intermediate *m*, or when *k*_m_ is regulated with small *m*. (**D**) Distribution of mRNA numbers in the telegraph model and event model. The transcription has the same average dynamics in both models. The parameters for the telegraph model are *k*_m_ = 10, *k*_off_ = 0.1, 1 and 9, and *k*_on_ = 0.1, 1, 10 or 100, which are normalized by $\delta_{m}$. When $k_{\mathrm{off}}$ is close to $k_{m}$ (with small burst size) or the duration of the active phase is close to that of the inactive phase, the distributions in the two models are obviously different.


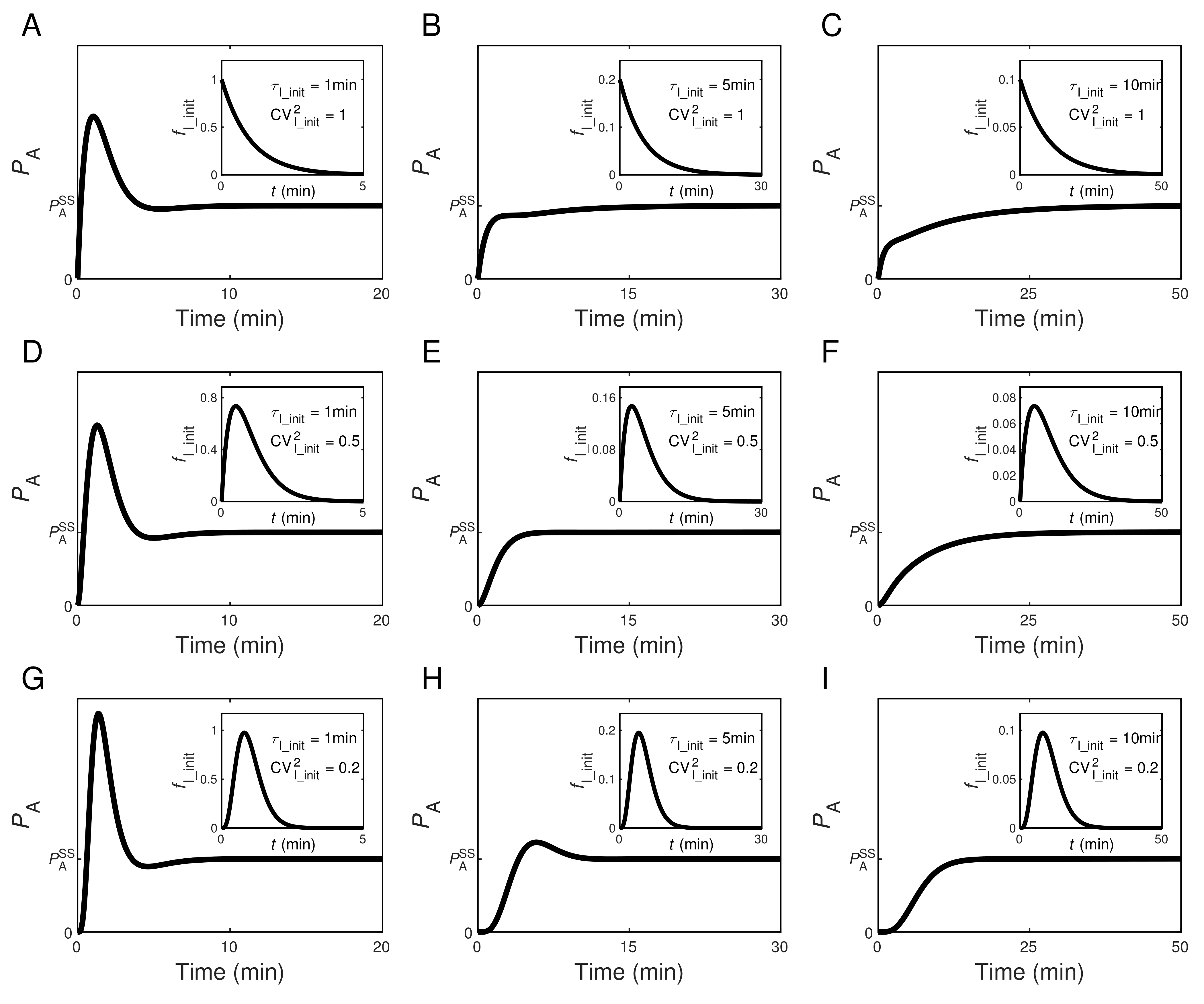


**Figure** **S10.** Response curves for different distribution functions of the time taken to activate the gene from the initial state. The durations of the active phase (*f*_A_) and inactive phase (*f*_I_) are gamma distributed ($f_{i}=\frac{\beta^{\alpha_{i}}}{\Gamma(\alpha_{i})}t^{\alpha_{i}-1}e^{-\beta_{i}t};\alpha_{i}=\frac{1}{\mathrm{CV}_{i}^{2}}, \beta_{i}=\frac{\alpha_{i}}{\tau_{i}}$) with *τ*_A_ = 1 min, CV_A_^2^ = 1, *τ*_I_ = 5 min, and CV_I_^2^ = 0.5. *P*_A_^SS^is the gene activity at steady state after stimulation, equaling 1/6. The distribution of the time required to first enter the active phase after stimulation is *f*_I_init_ (inset). The CV^2^ of *f*_I_init_ (CV_I_init_^2^) is 1 (**A-C**), 0.5 (**D-F**), or 0.2 (**G-I**). The mean of *f*_I_init_ (*τ*_I_init_) is 1 min (**A, D, G**), 5 min (**B, E, H**), or 10 min (**C, F, I**). When the CV_I_init_^2^ or *τ*_I_init_ is small, the gene is easy to overshoot (**A, D, G, H**).


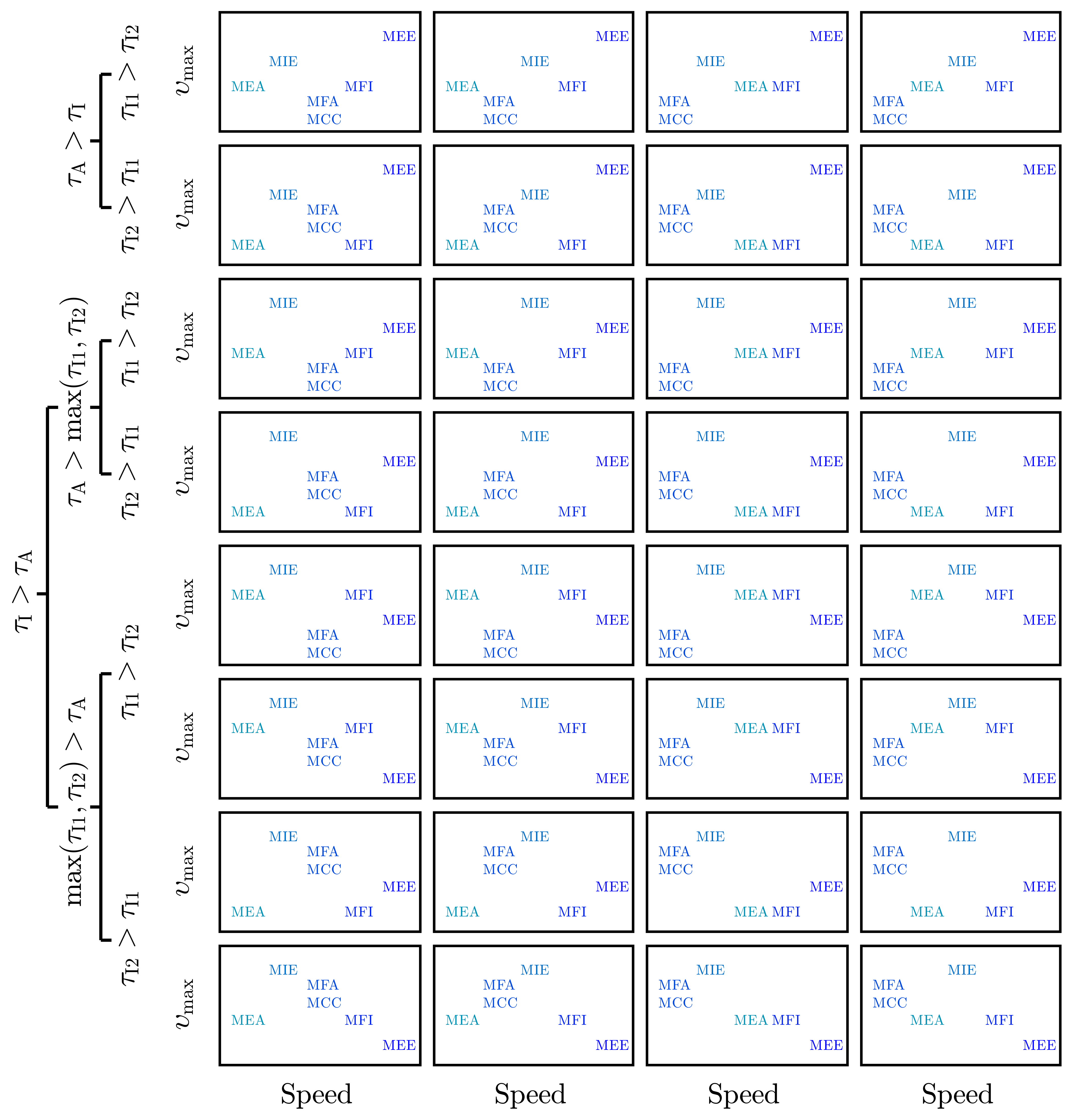


**Figure** **S11.** Regulatory range (*υ*_max_) and response speed for basic regulatory modes (MIE, MEE, MFA, MEA, MFI and MCC). Initially, the gene is completely silent without DNA-bound regulators. MEE elicits the fastest response and maximum regulation range for *τ*_A_ > *τ*_I_. For *τ*_A_ < *τ*_I_, $\mathrm{MIE}$ evokes the maximum regulation range.


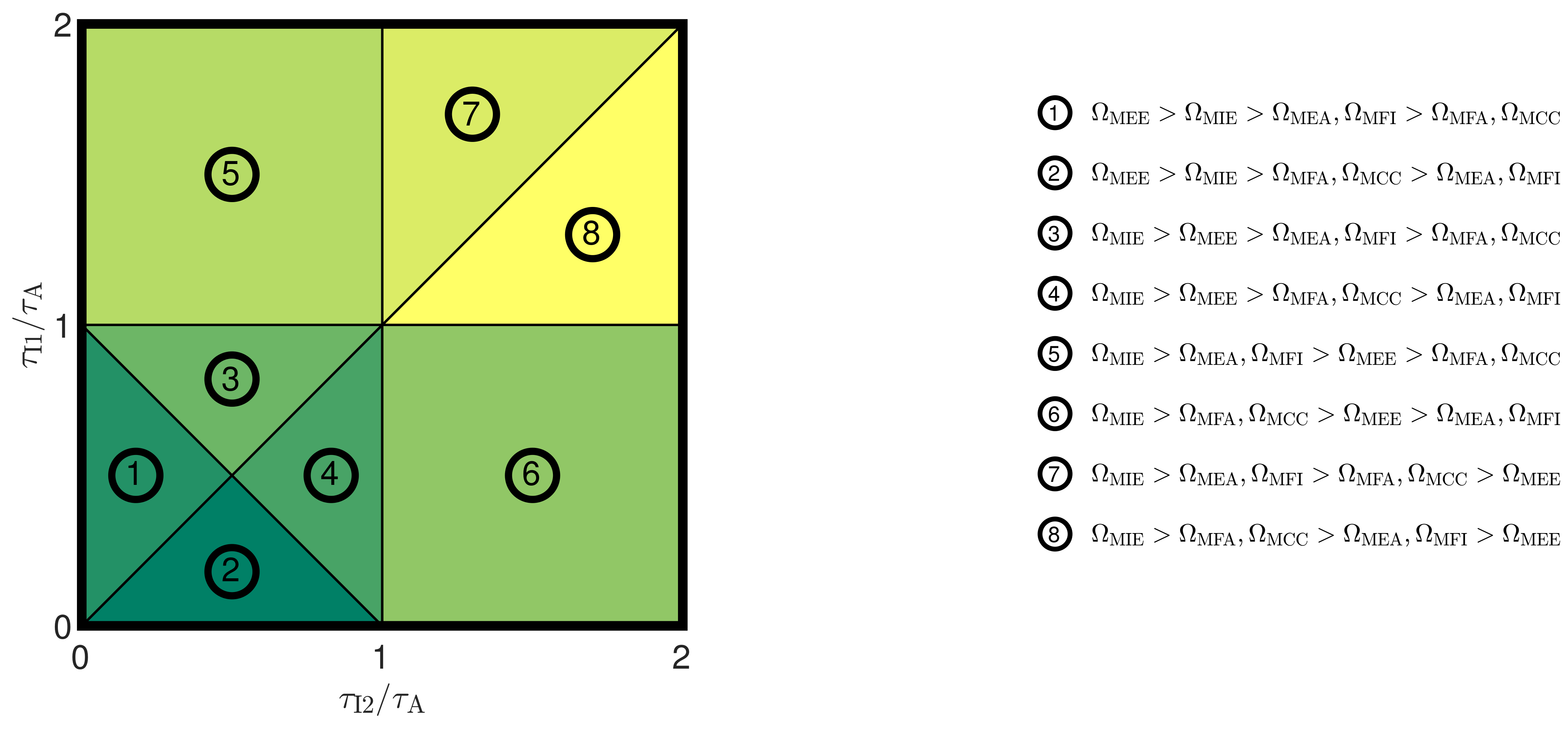


**Figure S12.** Transcriptional sensitivity under different regulatory modes. There exist eight scenarios in terms of the operating point $\Omega$(smaller than $K_{d}$); the same basal transcription and fold change are kept in each mode. All the following variables describe the case without regulator binding. $\tau_{A}$ is the average duration of the active phase, equaling *τ*_P_/(1-*p*_1_). *τ*_I1_ is the average total duration of *E*_S1_ and *E*_S2_ in a burst, equaling (*τ*_S1_+*τ*_S2_)/*p*_1_. *τ*_I2_ is the average total duration of *E*_S3_ in a burst, equaling $\frac{1}{p_{1}}\frac{p_{2}}{1-p_{2}}\tau_{S3}$. When the average duration of the active phase is longer than that of the inactive phase, the largest Ω is acquired via MEE or via MIE when *τ*_A_ < *τ*_I1_+*τ*_I2_.

**Reference**

[1] Suter,D.M., Molina,N., Gatfield,D., Schneider,K., Schibler,U. and Naef,F. (2011) Mammalian genes are transcribed with widely different bursting kinetics. *Science* **332**, 472.

[2] Gillespie,D.T. (2001) Approximate accelerated stochastic simulation of chemically reacting systems. *J. Chem. Phys*. **115**, 1716.

[3] Gillespie,D.T. (1997) Exact stochastic simulation of coupled chemical-reactions. *J. Phys. Chem*. **81**, 2340.

[4] Toni,T., Welch,D., Strelkowa,N., Ipsen,A. and StumpF,M.P.H. (2009) Approximate Bayesian computation scheme for parameter inference and model selection in dynamical systems. *J. R. Soc. Interface* **6**, 187.

[5] Gebhardt,J.C.M., Suter,D.M., Roy,R., Zhao,Z.W., Chapman,A.R., Basu,S., Maniatis,T. and Xie,X.S. (2013) Single-molecule imaging of transcription factor binding to DNA in live mammalian cells. *Nat. Methods* **10**, 421.

[6] Chen,J. *et al.* (2014) Single-molecule dynamics of enhanceosome assembly in embryonic stem cells. *Cell* **156**, 1274.
